# Molecular docking simulation studies on potent butyrylcholinesterase inhibitors obtained from microbial transformation of dihydrotestosterone

**DOI:** 10.1186/1752-153X-7-164

**Published:** 2013-10-08

**Authors:** Salman Zafar, M Iqbal Choudhary, Kourosh Dalvandi, Uzma Mahmood, Zaheer Ul-Haq

**Affiliations:** 1 H. E. J. Research Institute of Chemistry, International Center for Chemical and Biological Sciences, University of Karachi, Karachi- 75270, Pakistan; 2 Department of Chemistry, Sarhad University of Science and Information Technology, Peshawar 25000, Pakistan; 3 Dr. Panjwani Center for Molecular Medicine and Drug Research, International Center for Chemical and Biological Sciences, University of Karachi, Karachi 75270, Pakistan; 4 Department of Biochemistry, Faculty of Science, King Abdulaziz University, Jeddah- 21412, Saudi Arabia

**Keywords:** Microbial transformation, Dihydrotestosterone (DHT), *Macrophomina phaseolina*, *Gibberella fujikuroi*, Butyrylcholinesterase (BChE) inhibition, Alzheimer’s disease, Molecular docking simulation

## Abstract

**Background:**

Biotransformation is an effective technique for the synthesis of libraries of bioactive compounds. Current study on microbial transformation of dihydrotestosterone (DHT) (**1**) was carried out to produce various functionalized metabolites.

**Results:**

Microbial transformation of DHT (**1**) by using two fungal cultures resulted in potent butyrylcholinesterase (BChE) inhibitors. Biotransformation with *Macrophomina phaseolina* led to the formation of two known products, 5α-androstan-3β,17β-diol (**2**), and 5β-androstan-3α,17β-diol (**3**), while biotransformation with *Gibberella fujikuroi* yielded six known metabolites, 11α,17β-dihydroxyandrost-4-en-3-one (**4**), androst-1,4-dien-3,17-dione (**5**), 11α-hydroxyandrost-4-en-3,17-dione (**6**), 11α-hydroxyandrost-1,4-dien-3,17-dione (**7**), 12β-hydroxyandrost-1,4-dien-3,17-dione (**8**), and 16α-hydroxyandrost-1,4-dien-3,17-dione (**9**). Metabolites **2** and **3** were found to be inactive, while metabolite **4** only weakly inhibited the enzyme. Metabolites **5**–**7** were identified as significant inhibitors of BChE. Furthermore, predicted results from docking simulation studies were in complete agreement with experimental data. Theoretical results were found to be helpful in explaining the possible mode of action of these newly discovered potent BChE inhibitors. Compounds **8** and **9** were not evaluated for enzyme inhibition activity both *in vitro* and *in silico*, due to lack of sufficient quantities*.*

**Conclusion:**

Biotransformation of DHT (**1**) with two fungal cultures produced eight known metabolites. Metabolites **5**–**7** effectively inhibited the BChE activity. Cholinesterase inhibition is among the key strategies in the management of Alzheimer’s disease (AD). The experimental findings were further validated by *in silico* inhibition studies and possible modes of action were deduced.

## Background

Microbial transformation of steroids is being studied for decades but the need to develop new structural analogues remain strong due to multiple reasons, including quest for medicinally important novel steroids [1]. In continuation of our recent work on the microbial transformation of important steroids [2], we investigated the microbial biotransformation of DHT. The rationale was to produce the DHT analogues for studying the structure-activity-relationship (SAR) and to synthesize medicinally important compounds with novel activities.

**Figure 1:**
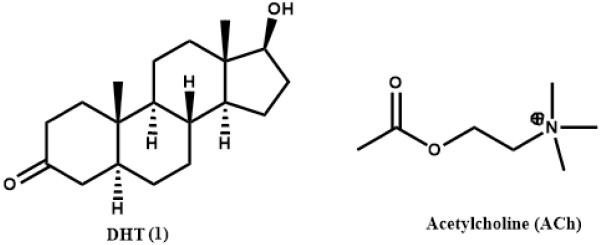
Structures of compounds used in the current study.

DHT (**1**) Figure 1, plays a vital role in the growth and differentiation of ventral prostate. DHT is selectively retained by an androgen receptor, found in the nuclear chromatin of prostate [3]. Due to its weaker interaction with the androgen receptor as compared to testosterone, DHT has a stronger androgenic potency [4]. It also plays a vital role in human hair loss [5].

Butyrylcholine (BCh) is an acetylcholine-like compound (Figure 1), hydrolyzed by acetylcholinesterase (AChE) and BChE (pseudocholinesterase), the latter being more efficient. BCh is a synthetic compound, used as a tool to distinguish between acetyl- and BChEs. BChEs is essential for the catalysis of the rapid breakdown of suxamethonium (succinylcholine), a muscle relaxant, frequently used in surgery and electroshock therapy. Prolonged muscle relaxation may follow the administration of drug if pseudocholinesterase activity is defective or markedly decreased [6,7]. BChE inactivates the neurotransmitter, acetylcholine (ACh). ACh is an important therapeutic target for the treatment of Alzheimer’s disease, characterized by a cholinergic deficit [8].

DHT was subjected to microbial transformation with two fungal cultures, *Macrophomina phaseolina* and *Gibberella fujikuroi* and eight known metabolites **2**–**9** were obtained. Substrate **1** and its metabolites **2**–**7** were subjected to AChE and BChE inhibitory activity evaluation. All compounds were found to be inactive against AChE, while metabolite **5**–**7** have significantly inhibited the BChE. Compounds **8** and **9** were not subjected to the above mentioned activity due to lack of sufficient quantities.

Protein-ligand docking programs are used for the placement of small molecules within the binding pocket of target proteins (receptors) and to rank them according to their binding affinity [9,10]. In current study, biotransformed products **2**–**7** were also evaluated *in silico* to understand their mode of interaction with the BChE. Resolved crystal structure of BChE was used in molecular docking simulation studies. All biotransformed metabolites were docked within the binding pocket of the crystal structure of human BChE (PDB ID 1P0P: 2.30 Å), revealing structural features, responsible of observed enzyme inhibitory activities [11]. MOE docking software was utilized to perform the molecular docking experiment. The outcome of the docking study helped to understand the binding mechanism of compounds with BChE.

## Results and discussion

**Figure 2:**
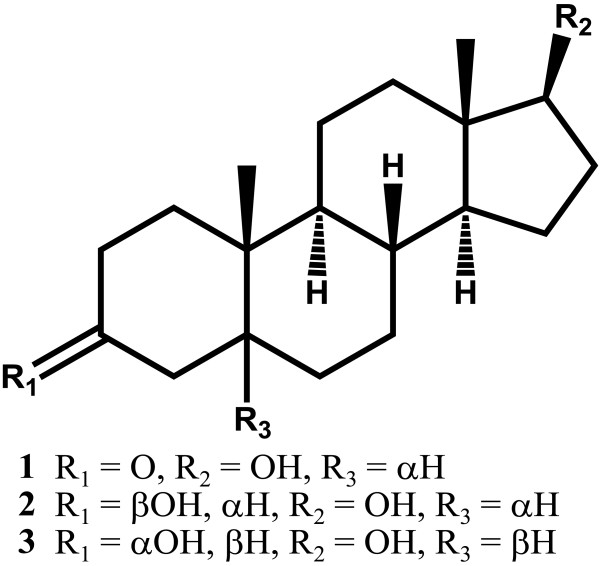
**Biotransformation of dihydrotestosterone (1) with *Macrophomina phaseolina*.**

**Figure 3:**
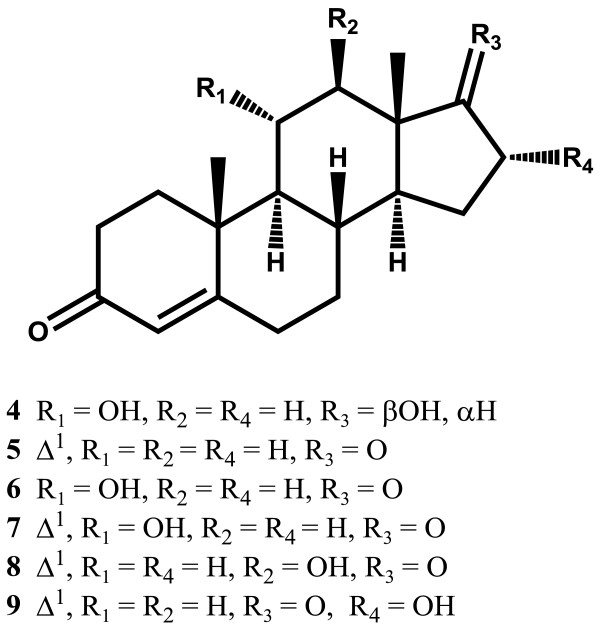
**Metabolites of biotransformation of DHT (1) with *Gibberella fujikuroi*.**

This is the first report of microbial transformation of DHT (**1**) (Figure 1), (C_19_H_30_O_2_) with *M. phaseolina* and *G. fujikuroi*. Fermentation of compound **1** with *M. phaseolina* for 6 days led to the formation of two known metabolites **2** and **3** (Figure 2), while 7 days fermentation of **1** with *G. fujikuroi* yielded six known metabolites **4**–**9** (Figure 3). Structure elucidation of all metabolites is presented below.

### Metabolites identification

Metabolite **2** (C_19_H_32_O_2_) (*M*^*+*^*m/z* 292.2434, calcd 292.2402) showed no florescence under UV light. The IR spectrum exhibited an absorption at 3350 cm^-1^ (OH), but no absorption for the ketone group was observed. This suggested that the 2 atomic mass units (a.m.u.) increase in the molecular weight might be due to the reduction of the ketone group of substrate **1** to a hydroxyl group in **2**.

The ^1^H-NMR spectrum of **2** showed two hydroxyl-bearing methine signals at δ 3.49 (m, H-3), and 3.54 (t, *J*_17a,16a,e_ = 8.4 Hz, H-17). The second hydroxyl signal was resulted from the reduction of the ketone at C-3. The ^13^C-NMR spectrum had two hydroxyl-bearing carbon signals at δ 71.8 (C-3) and 82.5 (C-17). H-3 (δ 3.49) showed HMBC correlation with C-5 (δ 46.3). H-5 (δ 1.10, m) also showed HMBC cross peaks with C-3 (δ 71.8) and C-10 (δ 36.7). The NOESY interactions between H-3 (δ 3.49) and H-5 (δ 1.10), as well as between H-5 and H-9 (δ 0.64, ddd, *J*_9a,8a_ = 14.8 Hz, *J*_9a,11a_ = 10.8 Hz, *J*_9a,11e_ = 4.4 Hz), indicated the α-orientation (*axial*) of H-3. H-17 (δ 3.54) showed NOESY interactions with H-14 (δ 0.92), suggesting its α-orientation (Additional file 1). The metabolite **2** was thus characterized as 5α-androstan-3β,17β-diol. The compound **2** has been earlier derived from DHEA (dehydroepiandrosterone) by the action of 17β-hydroxysteroid dehydrogenase type 7 enzyme [12]. 5α-Androstan-3β,17β-diol (**2**) has been reported to inhibit lipopolysaccharide induced inflammatory response and tumor necrosis factor in human endothelial cells [13].

The metabolite **3**, C_19_H_32_O_2_ [*M*^*+*^ = *m/z* 292.2434 (calcd 292.2402)], was UV inactive, thus lacked α,β-unsaturated carbonyl system. The IR spectrum indicated the presence of -OH (3349 cm^-1^), but no ketonic absorption. This could be due to the reduction of the carbonyl group at C-3.

The ^1^H-NMR spectral analysis indicated two hydroxyl-bearing methine-proton triplets at δ 3.94 (*J*_*3a,2/4*_ = 2.4 Hz, H-3), and 3.55 (*J*_17a,16a,e_ = 8.8 Hz, H-17), with their respective carbons resonated at δ 67.2 and 82.6, respectively, in the ^13^C-NMR spectrum (HMQC). HMBC of H-5 (δ 1.48) with carbon at δ 67.2 indicated the reduction of C-3 carbonyl into an OH. The H-3 (δ 3.94) showed NOESY correlation with H-5 (δ 1.48). Interestingly H-5 did not show any NOESY cross peak with H-9 (δ 0.74), indicating that H-5 is not α-oriented. Comparison of the ^1^H- and ^13^C-NMR chemical shift data of C-3 and C–5 of metabolites **2** and **3** showed marked differences which may be due to differences in the stereochemistry at these positions (Additional file 2). The metabolite was finally identified as 5β-androstan–3α,17β-diol, reported earlier as a biotransformation product of testosterone by fungus *Exophiala jeanselmei*[14]*.*

The molecular formula C_19_H_28_O_3_ [*M*^*+*^, *m/z* 304.2058] of metabolite **4** was deduced from the HREI-MS (calcd 304.2038). The presence of hydroxyl (3437 cm^-1^) and carbonyl (1667 cm^-1^) groups was inferred from the IR spectrum, while UV spectrum also indicated a conjugated ketone (λ_max_ = 233 nm).

The ^1^H-NMR analysis of **4** displayed some new signals as compared to the starting material DHT (**1**). A downfield methine signal at δ 4.02 (br. s, *W*_1/2_ = 22.0 Hz) and its respective carbon at δ 68.9, indicated the hydroxylation of one of the methylene carbon atoms. A downfield olefinic proton singlet at δ 5.71 (s) and the corresponding carbon at δ 124.6 indicated the introduction of a C = C bond. The HMBC spectrum displayed long-range couplings of the hydroxyl-bearing methine proton (δ 4.02) with C-9 (δ 59.2), C-10 (δ 39.9), and C-13 (δ 43.7), which suggested the position of the -OH at C-11. The stereochemical assignments were based on NOESY interactions between H-11 (δ 4.02), H-8 (δ 1.56), Me-19 (δ 1.31) and Me-18 (δ 0.81). H-11 was thus deduced as β-oriented. The olefinic proton (δ 5.71) showed HMBC with C-10 (δ 39.9), and C-2 (δ 34.2). The enone system was thus deduced at C-3/C-4-C-5, with the olefinic proton at C-4 (Additional file 3). Metabolite **4** was identified as 11α,17β-dihydroxyandrost-4-en-3-one. Hunter *et al*. in 2009 reported metabolite **4** from biotransformation of testosterone with fungus *Myceliophthora thermophila*[15], in an organotypic culture which represents *in vivo* situation. The cultures consisted of primary rat, porcine, and human hepatocytes [16].

Metabolite **5** (C_19_H_24_O_2_*M*^*+*^ at *m/z* 284.1726, calcd 284.1776) showed the presence of ketone (1730 cm^-1^) and a conjugated enone (1657 cm^-1^) in IR spectrum. UV spectrum showed a strong absorption for conjugated ketone (λ_max_ = 243 nm).

The ^1^H-NMR analysis of **5** showed three olefinic signals at δ 7.03 (d, *J*_1,2_ = 10.4 Hz), 6.22 (d, *J*_2,1_ = 10.4 Hz) and 6.07 (s). Their corresponding carbons were resonated at δ 155.3, 127.7, and 124.2, respectively. The ^13^C-NMR spectrum was devoid of any OH-bearing carbon signal which suggested the oxidation of the hydroxyl at C-17 into a ketone carbonyl. The conjugated ketonic group, inferred from UV analysis, was placed at C-3. The ^13^C-NMR spectra (Table 1) showed two ketonic carbonyl signals at δ 186.3 and 220.1 (Additional file 4). Metabolite **5** was thus characterized as androst-1,4-dien-3,17-dione. It was earlier reported as a urinary metabolite of testosterone [17]. Compound **5** was also previously obtained from the transformation of testosterone with *Steroidobacter denitrificans* strain FS^T^ under denitrifying conditions [18]. Soyabean phytosterols also yielded the same compound upon biotransformation with *Mycobacterium neoaurum*[19]. A mutant strain of *Mycobacterium*sp SH5 was utilized to convert phytosterols to androst-1,4-dien-3,17-dione [20]. Cholesterol was converted to compound **5** by biotransformation with bacteria *Chryseobacterium gleum*[21].

**Table 1:** ^**13**^**C-NMR Chemical shift data of compounds 1–9, δ in ppm**

**Compounds**									
**C**	**1**	**2**	**3**	**4**	**5**	**6**	**7**	**8**	**9**
1	38.7	38.3^a^	32.9	37.5	155.3	37.4	158.6	155.1	155.0
2	38.2	32.1	30.6	34.2	127.7	34.1	125.1	127.8	127.8
3	211.8	71.8	67.2	200.3	186.3	199.9	186.7	186.2	186.2
4	44.5	38.9	36.7	124.6	124.2	124.8	124.8	124.3	124.2
5	46.8	46.3	40.4	170.9	168.3	170.1	167.4	167.9	167.9
6	28.9	29.9	29.6	33.6	32.5	33.3	32.8	32.5	32.4
7	31.3	32.9	33.5	31.1	32.3	30.3	32.1	33.4	31.9
8	35.6	36.9	36.9	35.3	35.1	34.6	33.9	34.4	35.0
9	53.8	56.0	56.1	59.2	52.2	59.2	60.4	52.1	51.9
10	35.8	36.7	37.2	39.9	43.4	40.0	43.9	43.4	43.3
11	21.0	21.9	21.5	68.9	22.1	68.7	67.7	36.1	21.7
12	36.8	38.1^a^	38.1	48.5	31.1	42.9	42.3	86.0	30.9
13	43.1	44.1	44.1	43.7	47.7	47.9	47.8	42.7	38.7
14	50.9	52.4	52.5	49.8	50.4	50.0	49.6	44.1	47.3
15	23.5	24.3	24.3	23.3	21.9	21.7	21.8	22.2	30.6
16	30.6	30.6	29.7	30.6	35.6	35.7	35.8	35.5	71.1
17	81.9	82.5	82.6	81.0	220.1	218.4	218.3	216.2	218.3
18	11.1	11.7	11.7	12.3	13.8	14.6	14.6	11.5	14.1
19	11.5	12.8	11.6	18.4	18.7	18.3	18.7	18.7	18.7

Note:

^a^ interchangeable δ values.

CDCl_3_ at 125 MHz (**1**, **6**).

CDCl_3_ at 150 MHz (**4**, **5**, **7**, **8**, **9**).

CD_3_OD at 150 MHz (**2**).

CD_3_OD at 100 MHz (**3**).

Metabolite **6** (C_19_H_24_O_3_, *M*^*+*^ at *m/z* 302.1852, calcd 302.1882) showed the UV absorption at 234 nm for a conjugated ketone. The IR spectrum of **6** showed absorptions at 1668 (C = C-C = O), 1730 (C = O), and 3451 cm^-1^ (OH).

A methine proton at δ 4.04 (m, *W*_1/2_ = 21.5 Hz) was observed in the ^1^H-NMR spectrum of **6**, while its corresponding methine carbon resonated at δ 68.7 (C-11) in the ^13^C-NMR spectrum (Table 1). The COSY spectrum analysis indicated interactions between H-11 (δ 4.04) and H-9 (δ 1.15). The H-11 (δ 4.04), also showed HMBC cross peaks with C-13 (δ 47.9), C-10 (δ 40.0), and C-8 (δ 34.6), while NOESY cross peaks were observed between H-11 (δ 4.04), H-8 (δ 1.68), H-18 (δ 0.92), and H-19 (δ 1.31). Based on the above NOESY interactions, H-11 was assigned a β-orientation (Additional file 5). Complete spectral analysis indicated that the metabolite **6** was 11α-hydroxyandrost-4-en-3,17-dione. Koshimura and coworkers obtain compound **6**, by hydroxylation of androstenedione with *Gelasinospora retispora*[22]. Biotransformation of testosterone with *Fusarium lini* also afforded the same compound [23].

Molecular formula C_19_H_24_O_3_ (*M*^*+*^*m/z* 300.1749) was deduced from the HREI-MS of metabolite **7** (calcd 300.1725). The UV analysis suggested a conjugated enone system (λ_max_ 244 nm), while IR spectrum showed absorptions at 3386 (OH), 1731 (C = O) and 1658 cm^-1^ (C = C-C = O).

The ^1^H-NMR spectrum of metabolite **7** showed a downfield hydroxyl-bearing methine proton signal at δ 4.08, which was assigned to H/C-11. C-11 resonated at δ 67.7 in the ^13^C-NMR spectrum (Table 1) of **7**. Three olefinic protons appeared at δ 7.73 (d, *J*_1,2_ = 10.2 Hz), 6.14 (d, *J*_2,1_ = 10.2 Hz) and 6.08 (s), with respective carbons resonated at δ 158.6, 125.1, and 124.8 (Additional file 6). Based on the above spectral data and comparison with the literature [17], metabolite **7** was identified as 11α-hydroxy-androst-1,4-dien-3,17-dione, earlier obtained from the biotransformation of testosterone with *Fusarium lini*[23]. A strain of *Trichoderma hamatum* was utilized for the production of **7** from testosterone, testosterone propionate, androstenedione, 1-dehydrotestosterone, dianabol and progesterone [24].

Metabolite **8** (C_19_H_24_O_3_, *M*^*+*^*m/z* 300.1737, calcd 300.1725) showed UV absorption at 245 nm. The IR spectrum showed absorptions at 3389 (OH), 1730 (C = O) and 1658 cm^-1^ (C = C-C = O).

Metabolites **7** (Table 1) and **8** (Table 1) have structural similarities as evident from their NMR spectra. A methine proton at δ 3.73 (br. s, *W*_1/2_ = 13.0 Hz) was observed in the ^1^H-NMR of **8** (C at δ 86.0). The enone system in ring A of compound **8** showed the same HMBC interactions as in **7**. The C-17 ketone carbon (δ 216.2) showed HMBC correlation with the downfield C-12 methine proton (δ 3.73). The OH-bearing methine C-12 (δ 86.0) showed HMBC correlations with H-9 (δ 1.26), H-14 (δ 1.48), and CH_3_-18 (δ 0.78). The H-12 (δ 3.73) showed NOESY cross peaks with H-14 (δ 1.48), and H-9 (δ 1.26), and thus supported an α-orientation of H-12 (Additional file 7). The metabolite **8** was finally identified as 12β-hydroxyandrost-1,4-dien-3,17-dione, earlier isolated from *Halorrhena wulfsbergii* leaves [25]. Deoxycholate and β-sitosterol were previously converted to compound **8** by using immobilized *Pseudomonas* sp. and a thermophilic bacterium, respectively [26].

Metabolite **9** (C_19_H_24_O_3_, *M*^*+*^ = *m/z* 300.1749, calcd 300.1725), showed UV of 245 nm, while the IR spectrum displayed the same pattern as **8** [(3388 (OH), 1730, 1657 cm^-1^ (C = O)) for hydroxyl, ketone and enone functionalities, respectively].

The comparison of ^1^H-NMR spectra of **9** and **8** showed similarities, except for a methine signal at δ 4.36 (br s, *W*_1/2_ = 22.3 Hz) in compound **9** which appeared at δ 3.73 in metabolite **8**. The ^13^C-NMR spectrum (Table 1) of **9** showed this new methine carbon at δ 71.1. The proton, *geminal* to the -OH group (δ 4.36), was correlated with C-13 (δ 38.7), C-14 (δ 47.3) and C-17 (δ 218.3) in HMBC spectrum. The methine C-15 (δ 71.1) showed HMBC correlations with H-14 (δ 1.50) and H_2_-15 (δ 1.87, 1.98). Based on the above observations, the hydroxyl-bearing methine carbon was assigned to C-16. The H-16 (δ 4.36) showed NOESY cross peaks with H-18 (δ 1.01), but no interaction with H-14 (δ 1.50). Therefore the C-16 proton was deduced to be β-oriented (Additional file 8). The metabolite **9** was thus identified as 16α-hydroxyandrost-1,4-dien-3,17-dione. Numazwama *et. al.*, prepared 16α-hydroxyandrost-1,4-dien-3,17-dione (**9**) from androst-1,4-dien-3,17-dione upon reaction with hypervalent iodine [27].

### Enzyme inhibition assay

**Figure 4:**
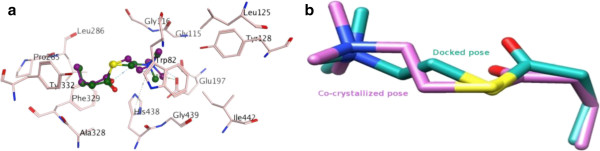
Three Dimensional (3D) conformational differences of co-crystallized ligand (BCH) and its docked pose within binding site of 1p0p.

Enzyme inhibition assay was carried out by using spectrophotometric method in 96-well plate. The experiments were conducted in triplicate. Substrate **1** showed a weak inhibition of the AChE. Therefore the substrate and its metabolites **2**–**7** were tested for AchE and BChE inhibitory activity. Interestingly all metabolites were found inactive against AChE. Metabolite **2** and **3** were also found to be inactive against the BChE, while metabolites **4**–**7** showed significant selective inhibition of BChE. Metabolites **5**–**7** were found to be the most active members (Table 2). Metabolites **2** and **3** lack a conjugated ketone system, while rest of the metabolites (i.e. **4**–**7**) have a α,β-unsaturated ketone system. The activity might be attributed to the presence of a conjugated system which may help the molecule attain a suitable configuration for binding to the active site of BChE. Metabolites **4**–**7** have approximately the same structural features, with conjugated ketone functionalities and hydroxyl groups, except **5**. Galanthamine was used as a standard inhibitor in the assay. Galanthamine is a potent cholinesterase inhibiting (approved by FDA in 2001) drug, which is used in clinical practices for the management of AD [28], Galanthamine is extensively used as standard to compare the potency of test compounds (IC_50_) [29] in biochemical assays. In docking study (Figure 4), galanthamine can bind to Trp 82 (anionic site) like active compounds **4-7**, it means binding site for compounds and galanthamine is similar, therefore all compounds can be compared with standard in both potency and function.

**Table 2:** **BChE Inhibitory activities (IC**_**50**_**) of compounds 1-7**

**Compounds**	**Mean ± SEM**^**a **^**[ *μ *** **M]**
**1**	NA^b^
**2**	NA^b^
**3**	NA^b^
**4**	109.4 ± 1
**5**	11.8 ±0.5
**6**	20.5 ±0.2
**7**	12.9 ±0.7
**Galanthamine (positive control)**	4.9 ± 0.3

^a^Data are expressed as mean ± standard error of mean.

^**b**^Not active.

### Molecular docking simulation

Initially, selection of suitable docking program for our target of interest was possible by re-docking with GOLD, MOE, and Surflex programs. In this exercise, re-docking protocol was applied on co-crystallized structure of human BChE (PDB ID 1P0P). The competency assessment of each re-docked pose was evaluated by considering the Root-mean-square deviation (RMSD) values, binding energy and refinement based re-scoring function, as shown in Table 3. MOE-Dock is able to produce the most convincing re-docking results for cognate ligand within the binding pocket of BChE (Figure 4, Table 4). Co-crystallized ligand and its re-docked pose are surrounded by the same active site residues displaying conserved interactions within 0.655 Å RMSD value. On the basis of satisfactory re-docking results, MOE was utilized to investigate the behavior of all bio-transformed DHT derivatives **2**–**7** inside the binding pocket of BChE.

**Table 3:** Re-docking score of co-crystallized ligand butyrylthiocholine (BCh) inside the binding pocket of BChE (PDB ID 1P0P)

**Serial number**	**Conformations**	**Binding free energy (Kcal/mol)**	**RMSD (Ǻ)**
1	BCh	-13.11	1.96
2	BCh	-11.32	1.51
**3**	**BCh**	**-13.91**	**0.65**

**Table 4:** Molecular docking studies of biotransformed DHT derivatives inside the binding cavity of BChE (PDB ID 1P0P)

**Compounds**	**Inhibition potency**	**IC**_**50**_ **± SEM [μM]**	**Binding free energy (Kcal/mol)**
**1**	Inactive	NA^c^	-6.64
**2**	Inactive	NA^c^	-6.73
**3**	Inactive	NA^c^	-6.43
**4**	Active	109.4 ± 1	-6.70
**5**	Active	11.8 ±0.5	-6.74
**6**	Active	20.5 ±0.2	-6.57
**7**	Active	12.9 ±0.7	-6.82
Galanthamine (Standard)	Active	4.9 ± 0.3	-6.26

**Figure 5:**
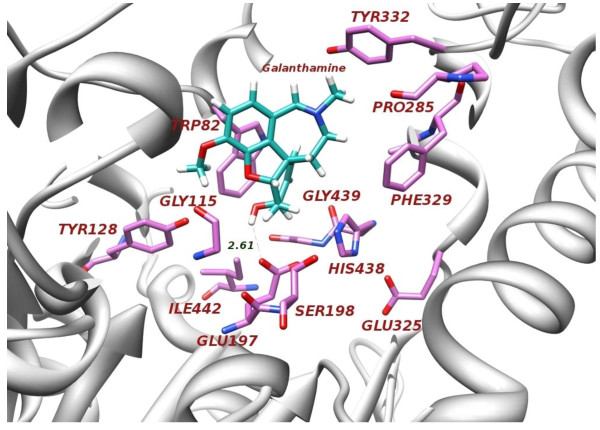
Docking conformation of galanthamine (generated by MOE docking software) properly accommodated into the binding cavity of BChE enzyme and developed hydrogen bond interaction with catalytic residue GLU197 at 2.61 Å.

**Figure 6:**
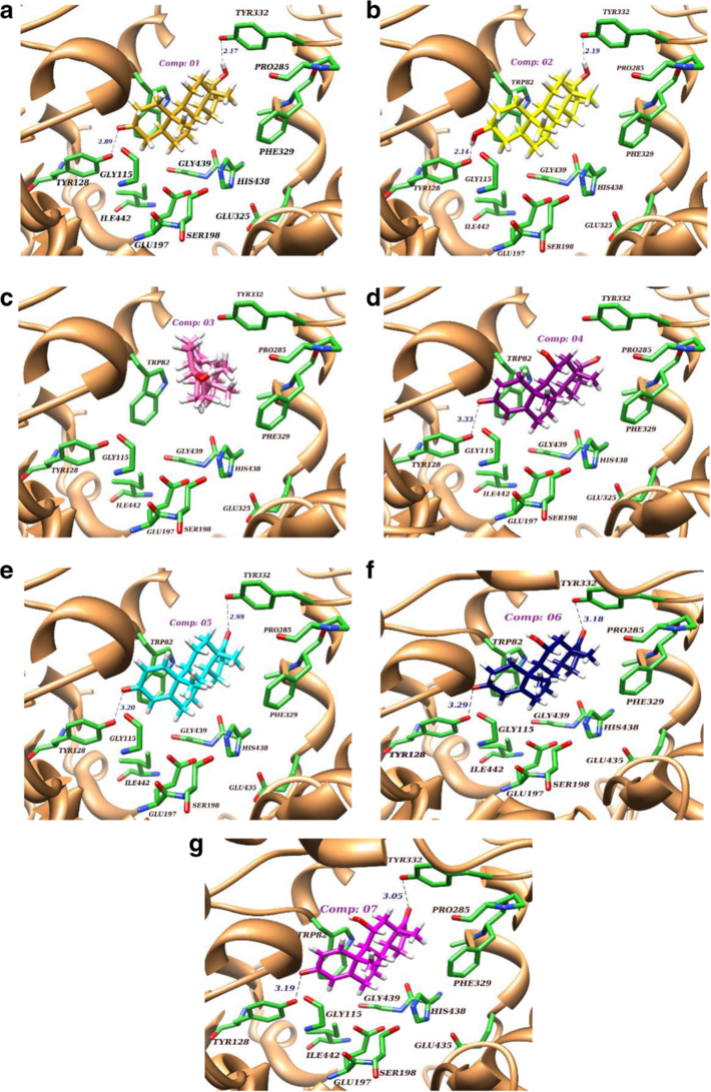
**Best selected molecular docking interactions pose of all compounds (1–7) within binding pocket of BChE.** Compounds **1**–**3** exhibited weak interaction due to the absence of π-π interaction **(a-c)**, while compounds **4**–**7** exhibited strong interactions **(d-g)** inside the BChE.

Molecular docking studies demonstrated that all compounds were well accommodated inside the binding pocket of BChE. Due to the larger binding pocket of BChE, bulkier compounds like DHT derivatives are easily placed themselves inside the binding gorge. The best selected dock pose of galanthamine, used as standard inhibitor, exhibited hydrogen bond interaction with catalytic triad residue Glu197 at 2.16 Å with all possible conserved interactions within 5.0 Å (Figure 5). From the analysis of various inhibitors (**1**–**7**), we conducted that due to lack of carbonyl moiety and a double bond in ring “A”, compounds **1**–**3** were not able to productively engage with the enzyme, the outcome was well correlated with experimental results. Both functional groups actively participated in the inhibition of BChE activity and are involved in the interactions with key residues, as shown in the Figure 6. The presence of both the functional groups in compounds seems to be prerequisite to inhibit the BChE activity. From this postulation, compounds **5**–**7** were identified as active inhibitors. Depth analysis exhibited the role of double bond in assisting the compound to attain a favorable orientation towards the binding residue TRP82 which is involved in the inhibition and thus participate in π-π interaction between the DHT derivatives and BChE. In active site, two most important residues of BChE (TYR128 and TYR332) are frequently involved in hydrogen bonding and play an important inhibitory role. By docking experiment, SER198, GLU197, HIS438, TYR128, TYR332, PRO285, PHE329, GLY115, GLY439 and TRP82 were identified as key residues, located within the binding pocket of BChE.

The Figure 6c clearly reflects that no interactions were found between compound**3** and binding residues of BChE, which might be due to the inverted orientation. Compound **3** exhibited no inhibition of the enzyme during the experimental studies. Similarly, compounds **1** and **2** also exhibited weak potency against BChE. By comparison of docked pose orientation of all compounds, it was deduced that compound **2** (Figure 6b) has a better orientation than compounds **1**, and **4**–**7**. With this orientation, two hydrogen bonds formation were also possible with the TYR128 (2.89 Å) and TYR332 (2.17 Å) residues of BChE, but the molecule would be disorientated for inhibition. This disturbance could be due to the presence of hydroxyl group (OH), instead of carbonyl moiety, and the absence of a double bond in ring “A” that was required for π-π interactions. Compound **4** was found to be more active as compared to **1**–**3**, while less active than compounds **5**–**7**. It would be due to the absence of hydrogen bonding with TYR332 (Figure 6d) even though it is involved in π-π interaction with TRP82. Compound **7** exhibited the highest inhibitory potency, experimentally and theoretically (-6.82 Kcal/mol), due to the double bond in ring “A”, as well as hydrogen bond interactions with TYR128 (3.19 Å) and TYR332 (3.05 Å) and hydrophobic interactions with key residues.

Docking results of compounds **2**–**7** with BChE provided valuable information about the nature of the binding interactions that were satisfactorily correlated with the experimental studies. This information could be utilized to design new leads against the BChE.

### Experimental

#### General

DHT was purchased from Acros-organics (Belgium). Thin layer chromatography was performed on precoated silica gel plates (PF_254_), purchased from Merck (Germany). Flash silica (E. Merck, Germany) was used for conducting column chromatography (CC). GS-320 (size exclusion) column was used for purification of compounds on preparative HPLC. ^1^H- and ^13^C-NMR experiments were conducted in CDCl_3_ and CD_3_OD on BrukerAvance-NMR spectrophotometers. The chemical shifts (δ values) are presented in ppm and the coupling constants (*J* values) in Hertz. JEOL JMS-600H mass spectrometer (Japan) was used for recording EI-MS in *m/z* (rel. %).

#### Microorganisms and culture medium

*Macrophomina phaseolina* (KUCC 730), obtained from Karachi University Culture Collection (KUCC), was grown on Sabouraud dextrose-agar (SDA) at 25°C and stored at 4°C. The medium for *M. phaseolina* was prepared by dissolving glycerol (10.0 mL), KH_2_PO_4_ (5.0 g), glucose (10.0 g), yeast extract (5.0 g), NaCl (5.0 g), and peptone (5.0 g) per litre of distilled H_2_O.

*Gibberella fujikuroi* (ATCC 10704) was purchased from the American Type Culture Collection. One litre of fermentation medium for *G. fujikuroi* was composed of glucose (20.0 g), NH_4_NO_3_ (1.0 g), KH_2_PO_4_ (5.0 g), MgSO_4_.7H_2_O (1.0 g) and trace element solution (2.0 mL). The trace element solution comprised of Co(NO_3_)_3_ (0.01 g), FeSO_4_.7H_2_O (0.1 g), CuSO_4_.5H_2_O (0.1 g), ZnSO_4_.7H_2_O (0.16 g), MnSO_4_. 7H_2_O (0.01 g), Mo(NH_4_)_3_ (0.01 g) in 100 mL distilled water.

#### Fermentation of DHT (1)

##### Fermentation with *Macrophomina phaseolina*

The fermentation medium for *M. phaseolina* was prepared by following the procedures presented above. 4.0 Litres of the medium was prepared and distributed (100 mL each) among 40 conical flasks (250 mL). The flasks were plugged with cotton and autoclaved at 121°C. The sterilized medium was innoculated with spores of *M. phaseolina*, obtained from already prepared slants on SDA. The flasks were placed on rotary shaker, and when enough growth was achieved, the flasks were fed uniformly with 1 g of DHT (**1**), dissolved in 20 mL acetone under aseptic conditions. The flasks were left over rotary shaker for 6 days. The fungal mass was filtered off. The filtrate was extracted with 12 L of dichloromethane (DCM). The DCM extract was dried over anhydrous Na_2_SO_4_, and concentrated on a rotary evaporator to obtain a brown gummy material (1.4 g). This gum was fractionated on a silica gel column with gradient petroleum ether- acetone solvent system. Three main fractions were obtained, which on further column chromatography led to the purification of metabolites **2** and **3**.

##### Microbial transformation of DHT (1) with *M. phaseolina*

The ^13^C-NMR chemical shifts of compounds **2** and **3** are shown in Table 1. Other data is presented below:

**5α-Androstan-3β,17β-diol (2)**. Colorless crystalline solid. **M.p.**: 153–155°C (154–157°C [30]). **[α]**^**25**^_**D**_ : -72.0, *c* = 0.034, MeOH (+6.20, c = 0.55, CHCl_3_[31]). ^**1**^**H-NMR** (CD_3_OD, 400 MHz): δ 0.97, m, 1.72^a^, m (H-1); 1.38, m, 1.73^a^, m (H-2); 3.49, m (H-3); 1.26, m, 1.50, m (H-4); 1.10, m (H-5); 1.28^a^, m, 1.31^a^, m (H-6); 0.89, m, 1.68, m (H-7); 1.40, m (H-8); 0.64, ddd (*J*_9a,8a_ = 14.8 Hz, *J*_9a,11a_ = 10.8 Hz, *J*_9a,11e_ = 4.4 Hz) (H-9); 1.30^a^, m, 1.56^a^, m (H-11); 1.02, m, 1.79, m (H-12); 0.92, m (H-14); 1.24, m, 1.57^a^, m (H-15); 1.45, m, 1.95, m (H-16); 3.54, t (*J*_17a,16a/16e_ = 8.4 Hz) (H-17); 0.71, s (H-18); 0.83, s (H-19), (a = exchangeable assignments). ^**13**^**C-NMR** (CD_3_OD, 150 MHz): See Table 1, **HREI-MS***m/z* (mol. formula, calcd value): 292.2434 (C_19_H_32_O_2_, 292.2402). **EI-MS***m/z* (rel. int., %): 292 [*M*^+^] (100), 277 (49), 259 (29), 248 (25), 233 (88), 215 (82), 201 (14), 175 (9), 165 (52), 121 (31), 107 (41), 55 (12).

**5β-Androstan-3α,17β-diol (3)**. Colorless crystalline solid. **M.p.**: 229–231°C (230–231°C [32]). **[α]**^**25**^_**D**_ : + 23.0, *c* = 0.028, MeOH (+ 26.0, ethanol [33]). ^**1**^**H-NMR** (CD_3_OD, 400 MHz): δ 1.66, m, 1.82, dt (*J*_1a,1e_ = 12 Hz, *J*_1a,2a/2e_ = 3.6 Hz) (H-1); 1.35, m; 1.69, m (H-2); 3.94, t (*J* = 2.4 Hz) (H-3); 1.45^a^, m; 1.59, m (H-4); 1.48^a^, m (H-5); 1.28^a^, m; 1.31, m (H-6); 1.27^a^, m; 1.39, m (H-7); 1.21, m (H-8); 0.74, m (H-9); 1.41, m; 1.47^a^, m (H-11); 0.99, m; 1.93, m (H-12); 0.89, m (H-14); 1.27^a^, m; 1.43, m (H-15); 1.46^a^, m; 1.56, m (H-16); 3.55, t (*J*_17a,16a/16e_ = 8.8 Hz) (H-17); 0.71, s (H-18); 0.82, s (H-19), (a = exchangeable assignments). ^**13**^**C-NMR** (CD_3_OD, 100 MHz): See Table 1. **HREI-MS***m/z* (mol. formula, calcd value): 292.2434 (C_19_H_32_O_2_, 292.2402). **EI-MS***m/z* (rel. int., %): 292 [*M*^+^] (66), 277 (51), 259 (33), 241 (20), 217 (54), 215 (100), 201 (14), 173 (11), 165 (45), 147 (33), 107 (47), 93 (48), 55 (37).

##### Fermentation with *Gibberella fujikuroi*

Two stage fermentation protocol (discussed above) was used for the fermentation of **1** (1.0 g) with *G*. *fujikuroi*. Fermentation medium was prepared as discussed earlier. Fermentation continued for 7 days. All the flasks were filtered, extracted with 12 litres of DCM and evaporated *in vacuo*. A brown extract (2.5 g) was obtained which was fractionated over a silica gel column with petroleum ether- acetone (P.E.- acetone) gradient solvent system. The fractions were compiled based on thin layer chromatography (TLC) profile to obtain five main fractions, which were again subjected to column chromatography (C.C.) (P.E.-acetone) and size exclusion high performance liquid chromatography (HPLC) by using a GS-320 column, and methanol as solvent system to afford pure metabolites **4**–**9**.

##### Microbial transformation of DHT (1) with *G. fujikuroi*

The ^13^C-NMR chemical shifts of compounds **4**–**9** are presented in Table 1. Other data is presented below:

**11α,17β-Dihydroxyandrost-4-en-3-one (4)**. Colorless crystalline compound. **M.p.**: 179–181°C (178–182°C [34]). **[α]**^**25**^_**D**_ : + 95.0, *c* = 0.096, MeOH (+94, *c* = 0.1, CHCl_3_[35]). ^**1**^**H-NMR** (CDCl_3_, 400 MHz): δ 2.62, dt (*J*_1a,1e_ = 14.0 Hz, *J*_1a,2a/2e_ = 4.0 Hz), 1.98, td (*J*_1e,2a/2e_ = 13.6 Hz, *J*_1e,1a_ = 4.0 Hz); 2.41, m; 2.31, m (H-2); 5.71, s (H-4); 2.37, m; 2.27, m (H-6); 1.81, m; 1.02, m (H-7); 1.56^a^, m (H-8); 1.05^a^, m (H-9); 4.02, br. s (*W*_1/2_ = 22.0 Hz) (H-11); 1.17, m; 2.13, m (H-12); 1.07^a^, m (H-14); 1.58^a^, m; 1.27, m (H-15); 1.45, m; 2.08, m (H-16); 3.77, t (*J*_17a,16a/16e_ = 8.0 Hz) (H-17); 0.81, s (H-18); 1.31, s (H-19), (a = exchangeable assignments). ^**13**^**C-NMR** (CDCl_3_, 150 MHz): See Table 1. **HREI-MS***m/z* (mol. formula, calcd value): 304.2058 (C_19_H_28_O_3_, 304.2038). **EI-MS***m/z* (rel. int., %): 304 [*M*^+^] (74), 286 (61), 253 (15), 180 (43), 163 (100), 147 (29), 137 (34), 124 (98), 109 (41), 91 (44), 79 (34), 55 (20).

**Androst-1,4-dien-3,17-dione (5)**. Colorless crystals. **M.p.**: 140–142°C (141–142°C [36]). **[α]**^**25**^_**D**_ : + 115.5 (*c* = 0.028, MeOH) (+117, CHCl_3_[37]). ^**1**^**H-NMR** (CDCl_3_, 400 MHz): δ 7.03, d (*J*_1,2_ = 10.4 Hz) (H-1); 6.22, d (*J*_2,1_ = 10.4 Hz) (H-2); 6.07, s (H-4); 2.41^a^, m, 2.48, m (H-6); 1.12, m, 2.06, m (H-7); 1.80, m (H-8); 1.09, m (H-9); 1.67, m, 1.84, m (H-11); 1.27^a^, m, 1.87, m (H-12); 1.26^a^, m (H-14); 1.57, m, 1.95, m (H-15); 2.10, m, 2.43^a^, m (H-16); 0.92, s (H-18); 1.24, s (H-19), (a = exchangeable assignments). ^**13**^**C-NMR** (CDCl_3_, 150 MHz): See Table 1. **HREI-MS***m/z* (mol. formula, calcd value): 284.1726 (C_19_H_24_O_2_, 284.1776). **EI-MS***m/z* (rel. int., %): 284 [*M*^+^] (45), 266 (8), 227 (6), 171 (8), 159 (66), 150 (26), 135 (31), 122 (100), 107 (36), 91 (46), 79 (19), 67 (10), 55 (10).

**11α-Hydroxyandrost-4-en-3,17-dione (6)**. Colorless crystalline compound. **M.p.**: 238–239°C (240–241°C [36]). **[α]**^**25**^_**D**_ : + 162.0, *c* = 0.034, MeOH (+ 165, CHCl_3_[38]). ^**1**^**H-NMR** (CDCl_3_, 400 MHz): δ 2.05, m; 2.65, dt (*J*_1a,1e_ = 13.6 Hz, *J*_1a,2a/2e_ = 4.4 Hz) (H-1); 2.31^a^, m; 2.39, m (H-2); 5.72, s (H-4); 2.29^a^, m; 2.37, m (H-6); 1.12, m; 1.97^a^, m (H-7); 1.68, m (H-8); 1.15, m (H-9); 4.04, m (*W*_1/2_ = 21.5 Hz) (H-11); 1.30, m; 2.11^a^, m (H-12); 1.37, m (H-14); 1.53, m; 1.95^a^, m (H-15); 2.14^a^, m; 2.48, m (H-16); 0.92, s (H-18); 1.31, s (H-19), (a = exchangeable assignments). ^**13**^**C-NMR** (CDCl_3_, 125 MHz): See Table 1. **HREI-MS***m/z* (mol. formula, calcd value): 302.1852 (C_19_H_26_O_3_, 302.1882). **EI-MS***m/z* (rel. int., %): 302 [*M*^+^] (24), 290 (35), 280 (21), 248 (51), 230 (95), 215 (21), 198 (19), 186 (28), 159 (31), 139 (100), 138 (88), 136 (52), 105 (29), 87 (65), 55 (18).

**11α-Hydroxyandrost-1,4-dien-3,17-dione (7)**. Colorless crystalline compound. **M.p.**: 208–210°C (212–214°C [34]). **[α]**^**25**^_**D**_ : + 85.1, *c* = 0.037, MeOH (+ 86.5 [34]). ^**1**^**H-NMR** (CDCl_3_, 300 MHz): δ 7.73, d (*J*_1,2_ = 10.2 Hz) (H-1); 6.14, d (*J*_2,1_ = 10.2 Hz) (H-2); 6.08, s (H-4); 2.41, m, 2.48, m (H-6); 1.15, m, 2.07, m (H-7); 1.82, ddd (*J*_8a,9a/14a_ = 22.8 Hz, *J*_8a,7a_ = 11.1 Hz, *J*_8a,7e_ = 3.9 Hz) (H-8); 1.12, m (H-9); 4.08, m (*W*_*1/2*_ = 19.6 Hz) (H-11); 1.25, m, 2.22, m (H-12); 1.35, m (H-14); 1.56, m, 1.95, m (H-15); 2.15, m, 2.50, m (H-16); 0.93, s (H-18); 1.31, s (H-19). ^**13**^**C-NMR** (CDCl_3_, 150 MHz): See Table 1. **HREI-MS***m/z* (mol. formula, calcd value): 300.1749 (C_19_H_24_O_3_, 300.1725). **EI-MS***m/z* (rel. int., %): 300 [*M*^+^] (36), 282 (18), 231 (80), 161 (20), 124 (37), 109 (32), 84 (58), 55 (23).

**12β-Hydroxyandrost-1,4-dien-3,17-dione (8)**. Colorless crystalline compound. **M.p.**: 163–165°C (164–166°C [39]). **[α]**^**25**^_**D**_ : + 84.0, *c* = 0.085, MeOH (+ 83.0, CHCl_3_[40]). ^**1**^**H-NMR** (CDCl_3_, 400 MHz): δ 7.03, d (*J*_1,2_ = 10.0 Hz) (H-1); 6.23, dd (*J*_2,1_ = 10.0 Hz, *J*_2,4_ = 1.2 Hz) (H-2); 6.03, br s (H-4); 2.39, m, 2.48, td (13.6, 4.4) (H-6); 1.15, m, 1.92, m (H-7); 1.81, m (H-8); 1.26, m (H-9); 1.42, m, 2.03, m (H-11); 3.73, br s (*W*_*1/2*_ = 13.0 Hz) (H-12); 1.48, m (H-14); 1.75, m, 1.86, m (H-15); 1.90, m, 2.33, m (H-16); 0.78, s (H-18); 1.25, s (H-19). ^**13**^**C-NMR** (CDCl_3_, 150 MHz): See Table 1. **HREI-MS***m/z* (mol. formula, calcd value): 300.1737 (C_19_H_24_O_3_, 300.1725). **EI-MS***m/z* (rel. int., %): 300 [*M*^+^] (66), 282 (8), 231 (100), 161 (20), 124 (37), 109 (32), 84 (58), 55 (43).

**16α-Hydroxyandrost-1,4-dien-3,17-dione (9).** Colorless crystals. **M.p.**: 152–153°C. **[α]**^**25**^_**D**_ : + 89.3 (*c* = 0.043, MeOH). ^**1**^**H-NMR** (CDCl_3_, 400 MHz): δ 7.00, d (*J*_1,2_ = 10.0 Hz) (H-1); 6.22, d (*J*_2,1_ = 10.0 Hz) (H-2); 6.04, s (H-4); 2.35, m, 2.45, m (H-6); 1.32, m, 2.03, m (H-7); 1.77, m (H-8); 1.10, m (H-9); 1.67, m, 1.85, m (H-11); 1.37, m, 1.65, m (H-12); 1.50, m (H-14); 1.87, m, 1.98, m (H-15); 4.36, br s (*W*_*1/2*_ = 20.8 Hz) (H-16); 1.01, s (H-18); 1.24, s (H-19). ^**13**^**C-NMR** (CDCl_3_, 150 MHz): See Table 1. **HREI-MS***m/z* (mol. formula, calcd value): 300.1749 (C_19_H_24_O_3_, 300.1725). **EI-MS***m/z* (rel. int., %): 300 [*M*^+^] (100), 272 (36), 246 (69), 239 (43), 216 (39), 200 (38), 190 (24), 165 (33), 147 (55), 108 (63), 92 (31), 81 (17).

### BChE inhibitory assay

All experiments were performed in a 96-well microplate in triplicate on SpectraMax340 (Molecular Devices, CA, U. S. A.).

Equine serum BChE, butyrylcholine chloride, and 5,5′-dithio-*bis* (2-nitrobenzoic) acid were purchased from Sigma. Analytical grade buffers and other chemicals were used. The BChE inhibition was measured by a modified spectrophotometric method [40]. Butyrylcholine chloride was used as substrate and the BChE inhibitory activity was measured by using 5-5′-dithio *bis* (2-nitrobenzoic) acid (DTNB).

150 μL of 100 mM sodium phosphate buffer [PBS] (pH 8), 10 μL of test compound solution (0.2 mM), and 20 μL BChE solution were mixed together and incubated at 25°C for 15 minutes. The reaction was initiated by the addition of 10 μL DTNB and 10 μL of BCh. A yellow 5-thio-2-nitrobenzoate anion was formed by hydrolysis of the substrate as a result of the reaction of DTNB with thiocholine which was monitored spectrophotometrically at 412 nm. MeOH was used as a solvent to dissolve the test compounds and the controls.

#### Determination of IC_50_

IC_50_ values were calculated by measuring the effect of varying concentrations of test compounds by using the EZ-Fit Enzyme Kinetic program. Galanthamine was used as a standard inhibitor of BChE.

### Computational methodology

Molecular modeling studies were conducted on a dual processor Intel® Xeon™ CPU 3.00 GHz LINUX work station running under SUSE 11.4. For the selection of best docking program with respect to the current biological system, GOLD [41], MOE [42] and Surflex [43,44] docking programs were employed. Molecular Operating Environment (MOE) docking software was finally selected to study the assembly pattern of biotransformed DHT derivatives in complex with human BChE system. Among thirty-one X-ray crystal structures of human BChE in the protein data bank (PDB) [11,45], PDB ID 1P0P: 2.30 Å was selected as the target protein based on suitable resolution and co-crystallized ligand, BCh. The entire system was energy minimized by MMFF94 force field [46], after adding the missing hydrogen atoms and keeping heavy atoms fixed until a Root-mean-square deviation (RMSD) gradient of 0.05 Kcalmol^-1^ Å^-1^ was reached. 3D structures of all metabolites **2**–**7** were drawn by molecule builder which is incorporated in MOE modeling package and the structures were subjected to MMFF94 for energy minimization. Subsequently for the evaluation of potential energy, partial charges were calculated by MMFF94 force field [47]. Both prepared systems (protein and ligand) were introduced for molecular docking simulation. Docking simulations were performed by using two methods (a) Alpha Triangle placement method, and (b) Triangle matcher placement method. All compounds were ranked with London dG scoring function and re-scored by GBVI/WSA dG with the force field refinement strategy [48]. A total of 30 docking poses were generated for each ligand and the pose with the lowest energy was selected for further studies.

## Conclusion

In summary, we report here the microbial transformation of dihydrotestosterone (DHT, **1**) for the first time by using suspension cultures of *M. phaseolina* and *G. fujikuroi*. Compounds **5**–**7** were found to be significant and specific inhibitors of the BChE, in comparison to standard drug, galanthamine. Current experimental studies revealed that the DHT derivatives actively participated in the inhibition of BChE. Additionally, experimental studies correlated well with theoretical studies thus indicating the behavior of different metabolites inside the binding pocket of BChE, and supported inhibition activity trend, followed by least active to highly active compounds. From the docking analysis, experimental inhibitory potency relationship of compounds **1**–**7** exhibited the fundamental role of α,β-unsaturated carbonyl moiety in ring “A”. In the bound state of metabolites with BChE, compounds **5**–**7** exhibited favorable hydrophilic, hydrophobic and hydrogen bond interactions with active site residues of the receptor protein. These compounds also participated in π-π interaction towards the TRP82.

## Competing interest

The authors declare that they have no competing interests.

## Author’s contributions

MIC envisioned the concept of the current study, supervised the practical work, helped in solving spectroscopic data and finalized the manuscript. SZ carried out the microbial transformation experiments, purified all the metabolites, interpreted the spectroscopic data and prepared the preliminary draft of the manuscript. KD conducted the biochemical assays for inhibition of BChE. UM conducted the *in silico* studies and wrote the simulation part of the manuscript. ZH supervised the computational studies. All authors read and approved the final manuscript.

## Supplementary Material

Additional file 1 ^1^H-, ^13^C- and 2D-NMR spectra of compound **2**. Click here for file

Additional file 2 ^1^H-, ^13^C- and 2D-NMR spectra of compound **3**. Click here for file

Additional file 3 ^1^H-, ^13^C- and 2D-NMR spectra of compound **4**. Click here for file

Additional file 4 ^1^H-, ^13^C- and 2D-NMR spectra of compound **5**. Click here for file

Additional file 5 ^1^H-, ^13^C- and 2D-NMR spectra of compound **6**. Click here for file

Additional file 6 ^1^H-, ^13^C- and 2D-NMR spectra of compound **7**. Click here for file

Additional file 7 ^1^H-, ^13^C- and 2D-NMR spectra of compound **8**. Click here for file

Additional file 8 ^1^H-, ^13^C- and 2D-NMR spectra of compound **9**. Click here for file
