# Supplementary material for: Molecular docking simulation studies on potent butyrylcholinesterase inhibitors obtained from microbial transformation of dihydrotestosterone
Source: Chem Cent J. 2013 Oct 8;7:164. doi: 10.1186/1752-153X-7-164 (PMC4126177; doi:10.1186/1752-153X-7-164)
Supplement: Additional file 1 — 1H-, 13C- and 2D-NMR spectra of compound 2. [file 1752-153X-7-164-S1.pdf]

AVANCE 400-B
LAB. No. 109

SALMAN/DR.IQBAL/DHT-1/CD3OD/

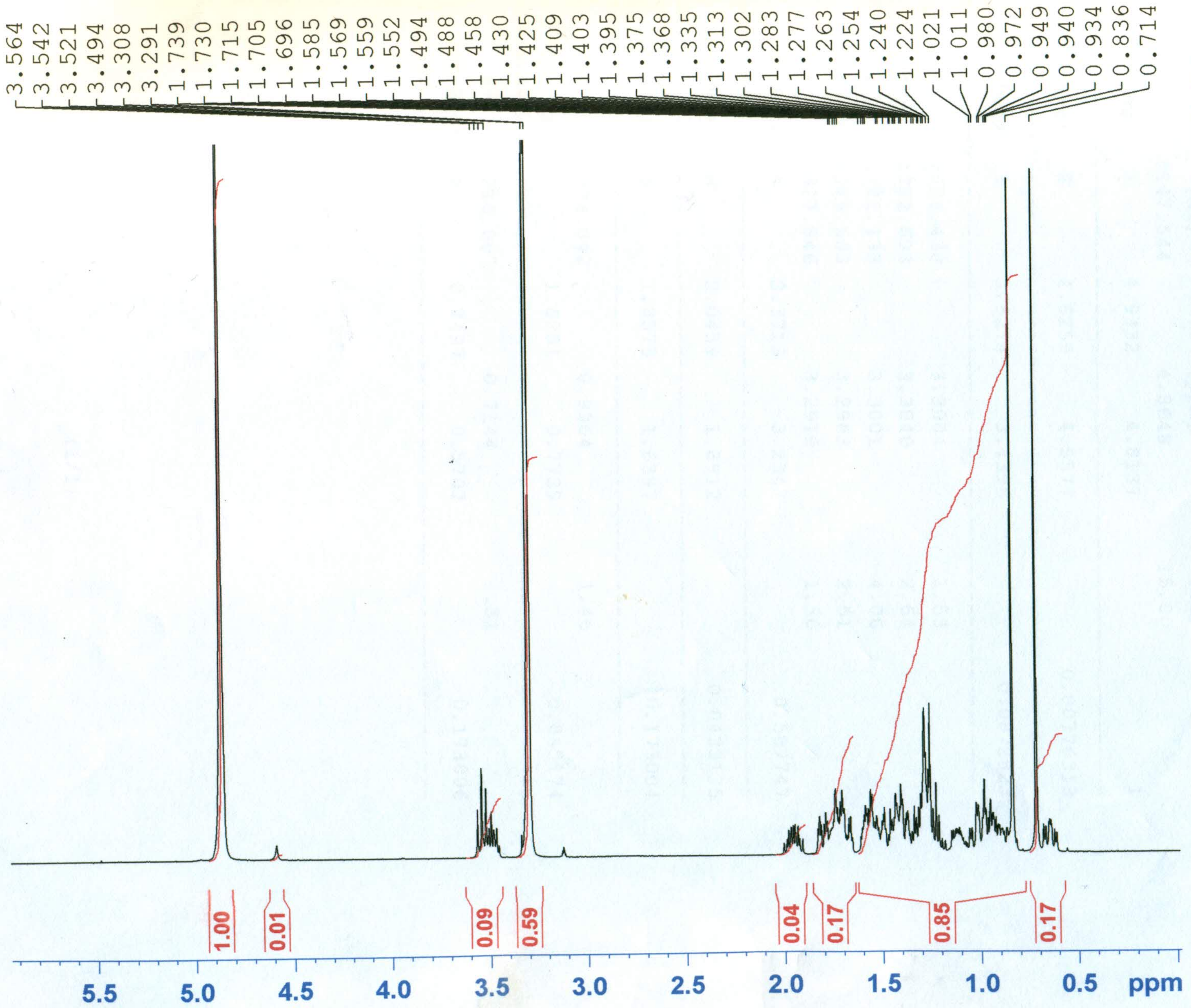

NAME	jan01
EXPNO	5
PROCNO	1
Date_	20090101
Time	12.11
INSTRUM	spect
PROBHD	5 mm DUL 13C-1
PULPROG	zg30
TD	32768
SOLVENT	MeOD
NS	128
DS	0
SWH	8012.820 Hz
FIDRES	0.244532 Hz
AQ	2.0447731 sec
RG	512
DW	62.400 usec
DE	6.50 usec
TE	300.0 K
D1	1.00000000 sec
TD0	1

======== CHANNEL f1 ========

NUC1	1H
P1	11.90 usec
PL1	-2.00 dB
SFO1	400.3332026 MHz
SI	16384
SF	400.3300099 MHz
WDW	EM
SSB	0
LB	0.30 Hz
GB	0
PC	1.00

Salman/Prof.Iqbal
Sample: DHT=1

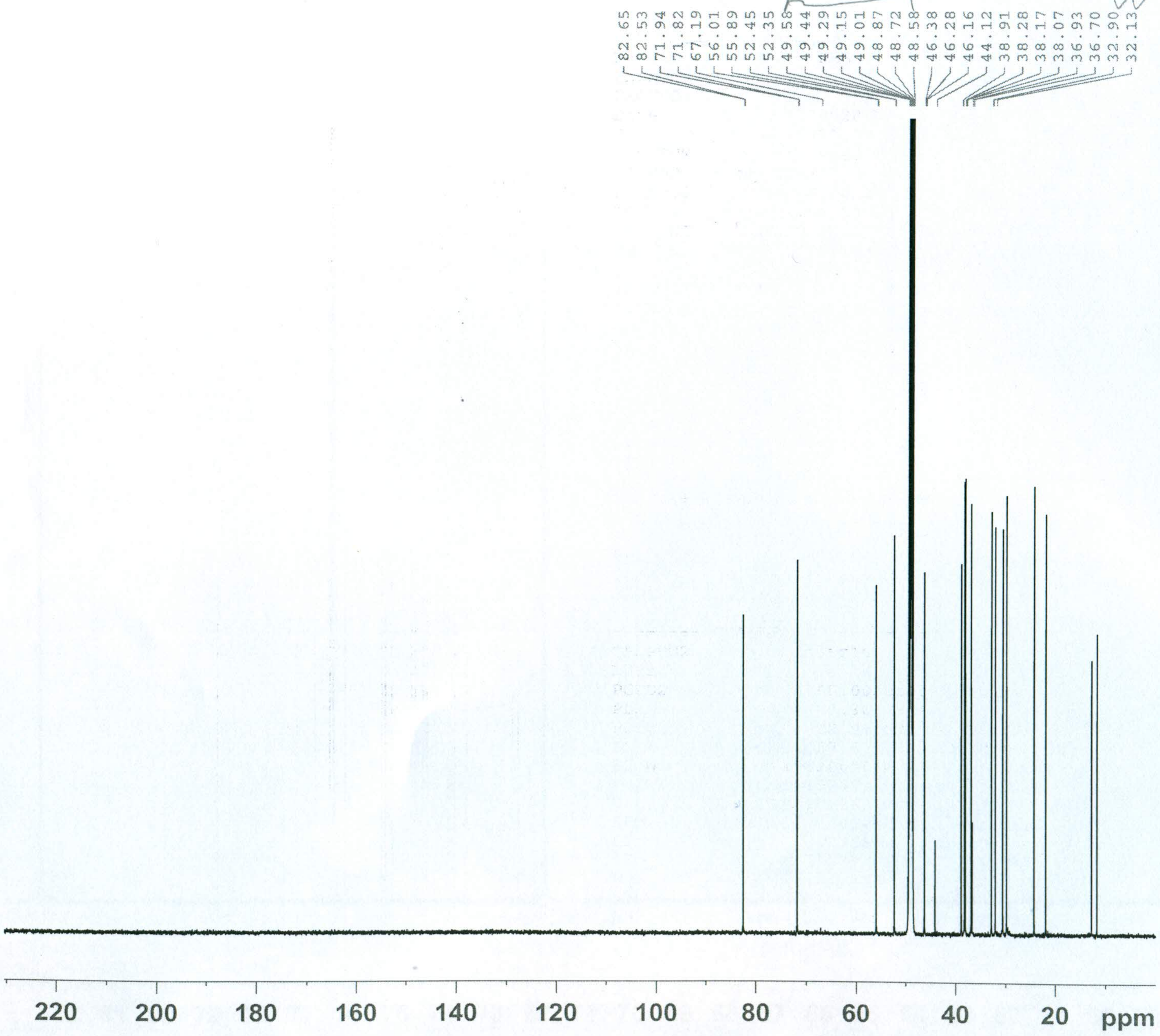

BRUKER

NAME	FEB06
EXPNO	6
PROCNO	1
Date_	20090206
Time	21.37
INSTRUM	spect
PROBHD	5 mm CPTCI 1H-
PULPROG	zgpg
TD	65536
SOLVENT	MeOD
NS	12288
DS	2
SWH	35971.223 Hz
FIDRES	0.548877 Hz
AQ	0.9110143 sec
RG	32768
DW	13.900 usec
DE	6.50 usec
TE	291.1 K
D1	1.50000000 sec
D11	0.03000000 sec
TD0	12

======== CHANNEL f1 ========

NUC1	13C
P1	16.00 usec
PL1	2.00 dB
PL1W	66.40702820 W
SFO1	150.9453107 MHz

======== CHANNEL f2 ========

CPDPRG2	waltz16
NUC2	1H
PCPD2	65.00 usec
PL2	3.30 dB
PL12	22.06 dB
PL13	27.00 dB
PL2W	9.16420078 W
PL12W	0.12192553 W
PL13W	0.03909260 W
SFO2	600.2336014 MHz
SI	32768
SF	150.9277397 MHz
WDW	EM
SSB	0
LB	1.00 Hz
GB	0
PC	1.40

Salman/Prof.Iqbal
Sample: DHT=1

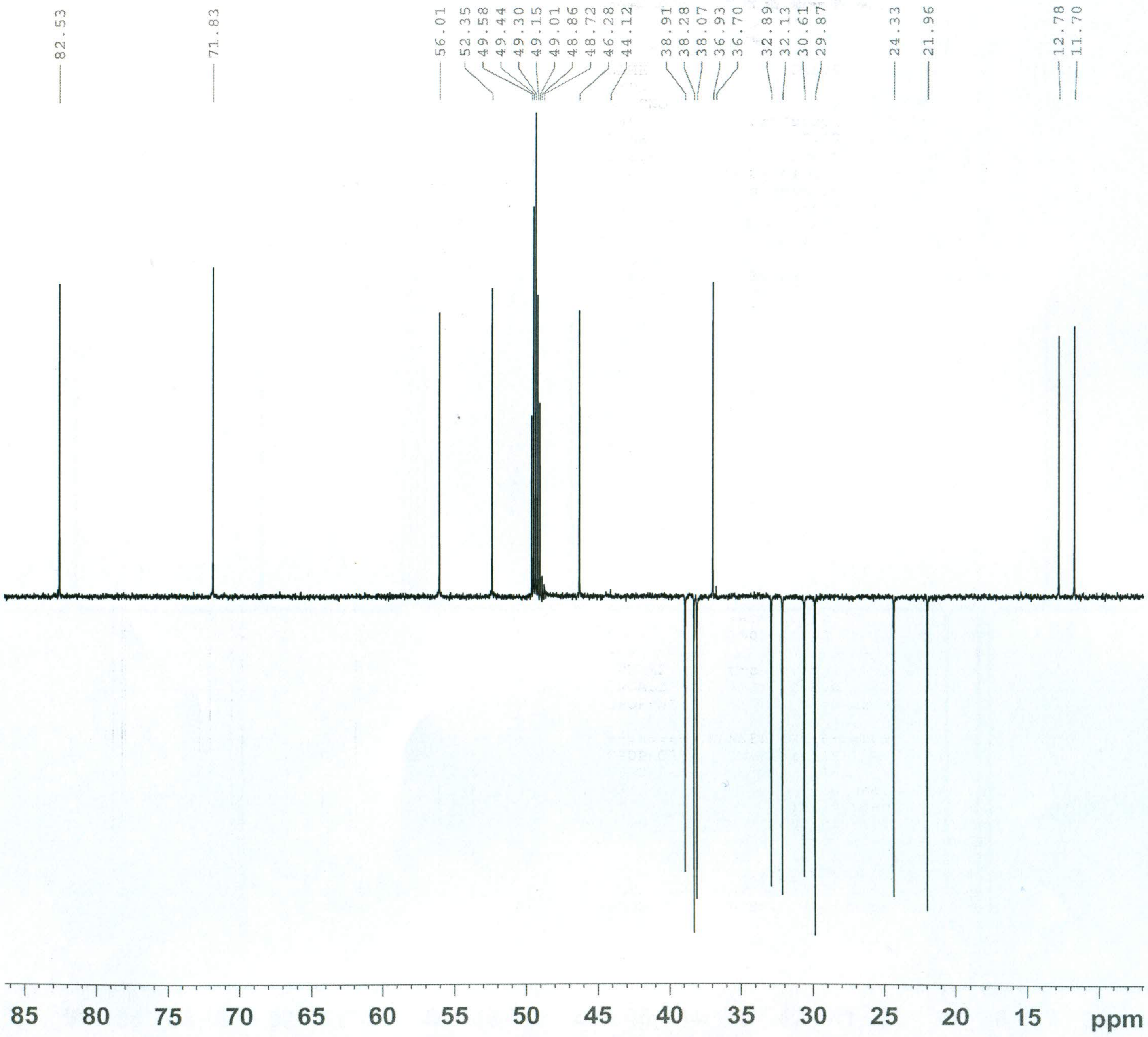

BRUKER

NAME	FEB06	
EXPNO	7	
PROCNO	1	
Date_	20090207	
Time	6.07	
INSTRUM	spect	
PROBHD	5 mm CPTCI 1H-	
PULPROG	deptsp135	
TD	65536	
SOLVENT	MeOD	
NS	4708	
DS	2	
SWH	30303.031	Hz
FIDRES	0.462388	Hz
AQ	1.0814105	sec
RG	32768	
DW	16.500	usec
DE	6.50	usec
TE	290.7	K
CNST2	145.0000000	
D1	1.50000000	sec
D2	0.00344828	sec
D12	0.00002000	sec
TD0	6	
======== CHANNEL f1 ========		
NUC1	13C	
P1	16.00	usec
P12	2000.00	usec
PL0	120.00	dB
PL1	2.00	dB
PL0W	0.00000000	W
PL1W	66.40702820	W
SFO1	150.9430463	MHz
SP2	1.99	dB
SPNAM2	Crp60comp.4	
SPOAL2	0.500	
SPOFFS2	0.00	Hz
======== CHANNEL f2 ========		
CPDPRG2	waltz16	
NUC2	1H	
P3	7.50	usec
P4	15.00	usec
PCPD2	65.00	usec
PL2	3.30	dB
PL12	22.06	dB
PL2W	9.16420078	W
PL12W	0.12192553	W
SFO2	600.2324009	MHz
SI	32768	
SF	150.9277397	MHz
WDW	EM	
SSB	0	
LB	1.00	Hz
GB	0	
PC	1.40	

[illegible]NCE AV 600
[illegible] No. 108

Salman/Prof.Iqbal
Sample: DHT=1

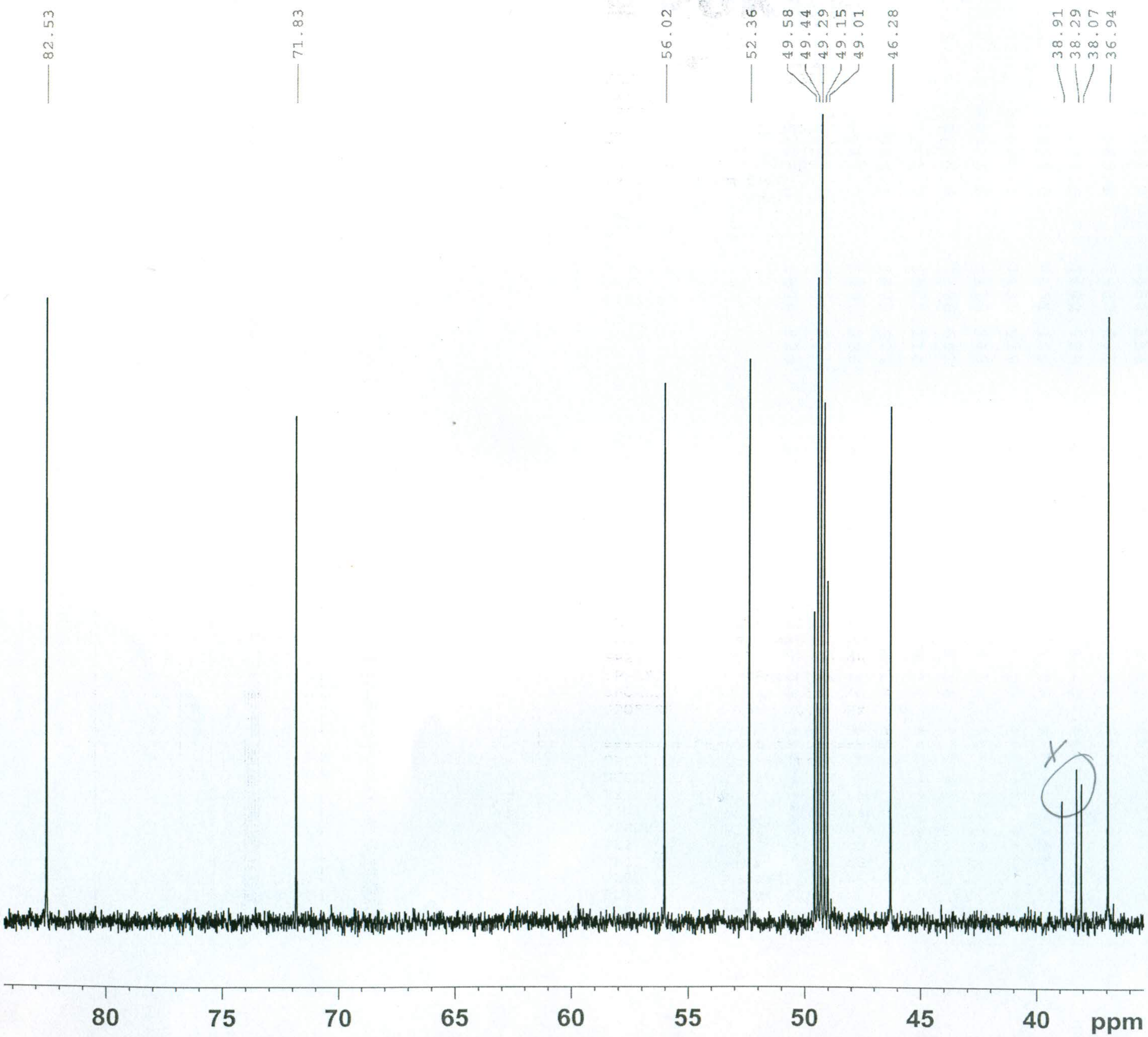

BRUKER

NAME	FEB06
EXPNO	8
PROCNO	1
Date_	20090207
Time	9.16
INSTRUM	spect
PROBHD	5 mm CPTCI 1H-
PULPROG	deptsp90
TD	65536
SOLVENT	MeOD
NS	634
DS	2
SWH	30303.031 Hz
FIDRES	0.462388 Hz
AQ	1.0814105 sec
RG	32768
DW	16.500 usec
DE	6.50 usec
TE	292.6 K
CNST2	145.0000000
D1	1.50000000 sec
D2	0.00344828 sec
D12	0.00002000 sec
TD0	6

======== CHANNEL f1 ========

NUC1	13C
P1	16.00 usec
P12	2000.00 usec
PL0	120.00 dB
PL1	2.00 dB
PL0W	0.00000000 W
PL1W	66.40702820 W
SFO1	150.9430463 MHz
SP2	1.99 dB
SPNAM2	Crp60comp.4
SPOAL2	0.500
SPOFFS2	0.00 Hz

======== CHANNEL f2 ========

CPDPRG2	waltz16
NUC2	1H
P3	7.50 usec
P4	15.00 usec
PCPD2	65.00 usec
PL2	3.30 dB
PL12	22.06 dB
PL2W	9.16420078 W
PL12W	0.12192553 W
SFO2	600.2324009 MHz
SI	32768
SF	150.9277397 MHz
WDW	EM
SSB	0
LB	1.00 Hz
GB	0
PC	1.40

Salman/Prof. Iqbal
Sample: DHT=1

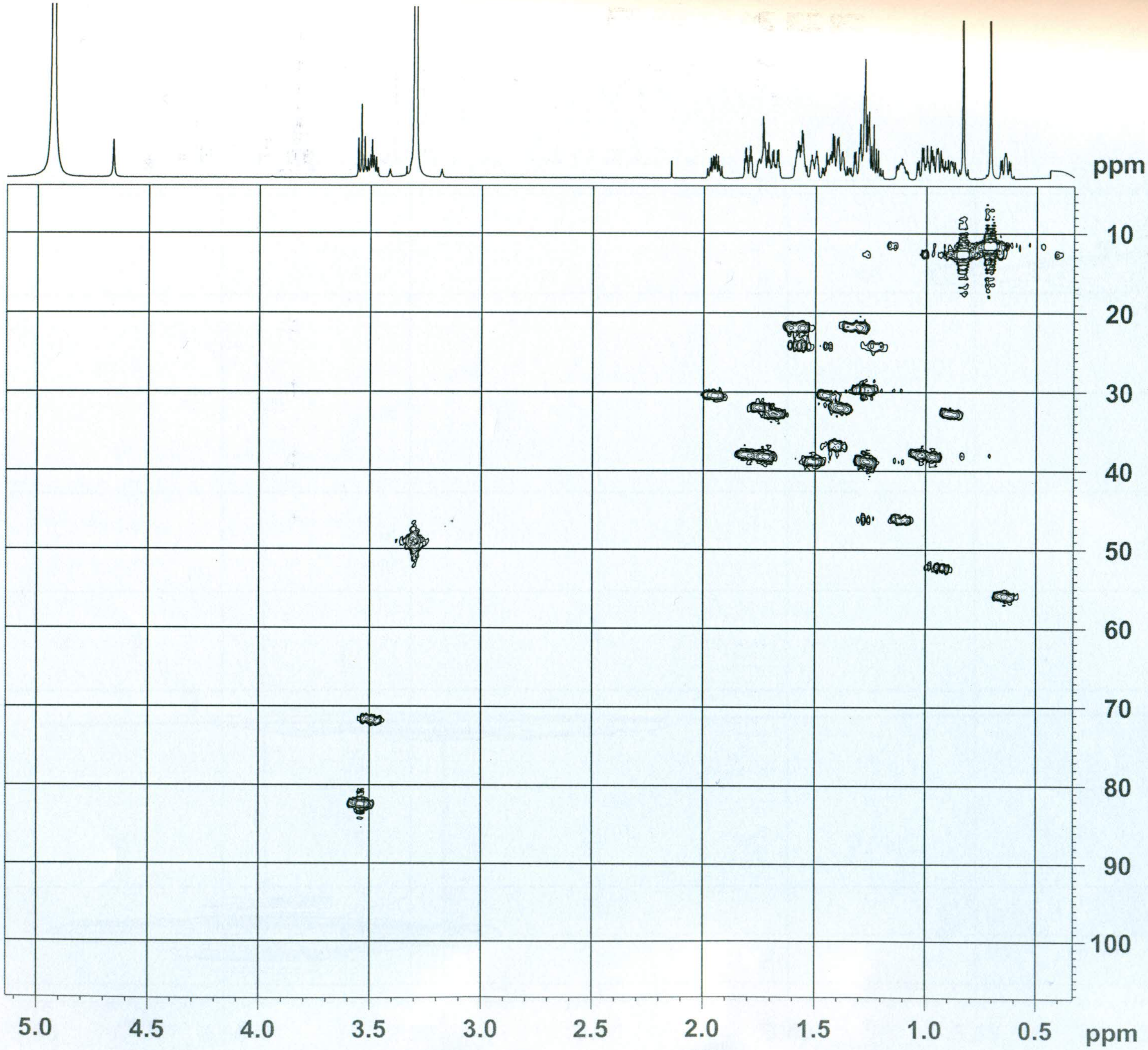

BRUKER

NAME	FEB06	
EXPNO	4	
PROCNO	1	
Date_	20090206	
Time	12.05	
INSTRUM	spect	
PROBHD	5 mm CPTCI 1H-	
PULPROG	hmqcgpqf	
TD	1024	
SOLVENT	MeOD	
NS	32	
DS	8	
SWH	3180.662	Hz
FIDRES	3.106115	Hz
AQ	0.1611800	sec
RG	46341	
DW	157.200	usec
DE	6.50	usec
TE	292.5	K
CNST2	145.0000000	
D0	0.00000300	sec
D1	1.50000000	sec
D2	0.00344828	sec
D12	0.00002000	sec
D13	0.00000400	sec
D16	0.00015000	sec
IN0	0.00001745	sec
======== CHANNEL f1 ========		
NUC1	1H	
P1	7.40	usec
P2	14.80	usec
PL1	3.30	dB
PL1W	9.16420078	W
SFO1	600.2318007	MHz
======== CHANNEL f2 ========		
CPDPRG2	garp	
NUC2	13C	
P3	10.00	usec
PCPD2	60.00	usec
PL2	2.00	dB
PL12	17.56	dB
PL2W	66.40702820	W
PL12W	1.84592509	W
SFO2	150.9422922	MHz
====== GRADIENT CHANNEL =====		
GPNAM1	SINE.100	
GPNAM2	SINE.100	
GPNAM3	SINE.100	
GPZ1	50.00	%
GPZ2	30.00	%
GPZ3	40.10	%
P16	2000.00	usec
ND0	2	
TD	256	
SFO1	150.9423	MHz
FIDRES	112.027481	Hz
SW	190.000	ppm
FnMODE	QF	
SI	1024	
SF	600.2300157	MHz
WDW	SINE	
SSB	0	
LB	0.00	Hz
GB	0	
PC	4.00	
SI	1024	
MC2	QF	
SF	150.9277397	MHz
WDW	SINE	
SSB	0	
LB	0.00	Hz
GB	0	

Salman/Prof.Iqbal
Sample: DHT=1

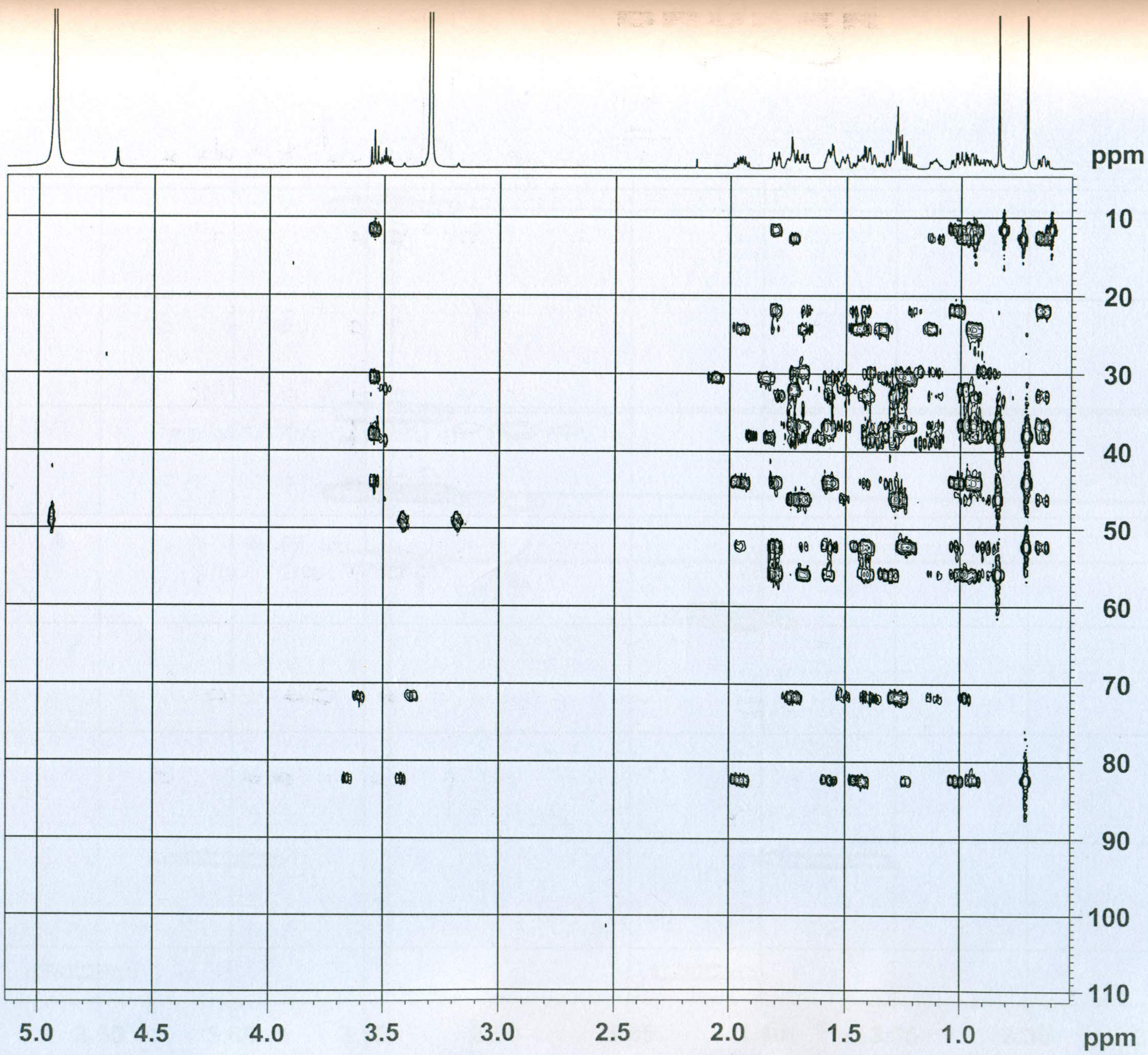

BRUKER

NAME	FEB06	
EXPNO	5	
PROCNO	1	
Date_	20090206	
Time	15.55	
INSTRUM	spect	
PROBHD	5 mm CPTCI 1H-	
PULPROG	hmbcgplpndqf	
TD	4096	
SOLVENT	MeOD	
NS	32	
DS	8	
SWH	3180.662	Hz
FIDRES	0.776529	Hz
AQ	0.6440984	sec
RG	32768	
DW	157.200	usec
DE	6.50	usec
TE	291.8	K
CNST2	145.0000000	
CNST13	13.0000000	
D0	0.00000300	sec
D1	1.50000000	sec
D2	0.00344828	sec
D6	0.03846154	sec
D16	0.00015000	sec
IN0	0.00001440	sec

======== CHANNEL f1 ========

NUC1	1H	
P1	7.40	usec
P2	14.80	usec
PL1	3.30	dB
PL1W	9.16420078	W
SFO1	600.2318007	MHz

======== CHANNEL f2 ========

NUC2	13C	
P3	10.00	usec
PL2	2.00	dB
PL2W	66.40702820	W
SFO2	150.9453107	MHz

====== GRADIENT CHANNEL =====

GPNAM1	SINE.100	
GPNAM2	SINE.100	
GPNAM3	SINE.100	
GPZ1	50.00	%
GPZ2	30.00	%
GPZ3	40.10	%
P16	2000.00	usec
ND0	2	
TD	256	
SFO1	150.9453	MHz
FIDRES	135.614929	Hz
SW	230.000	ppm
FnMODE	QF	
SI	1024	
SF	600.2300157	MHz
WDW	SINE	
SSB	0	
LB	0.00	Hz
GB	0	
PC	1.40	
SI	1024	
MC2	QF	
SF	150.9277397	MHz
WDW	SINE	
SSB	0	
LB	0.00	Hz
GB	0	

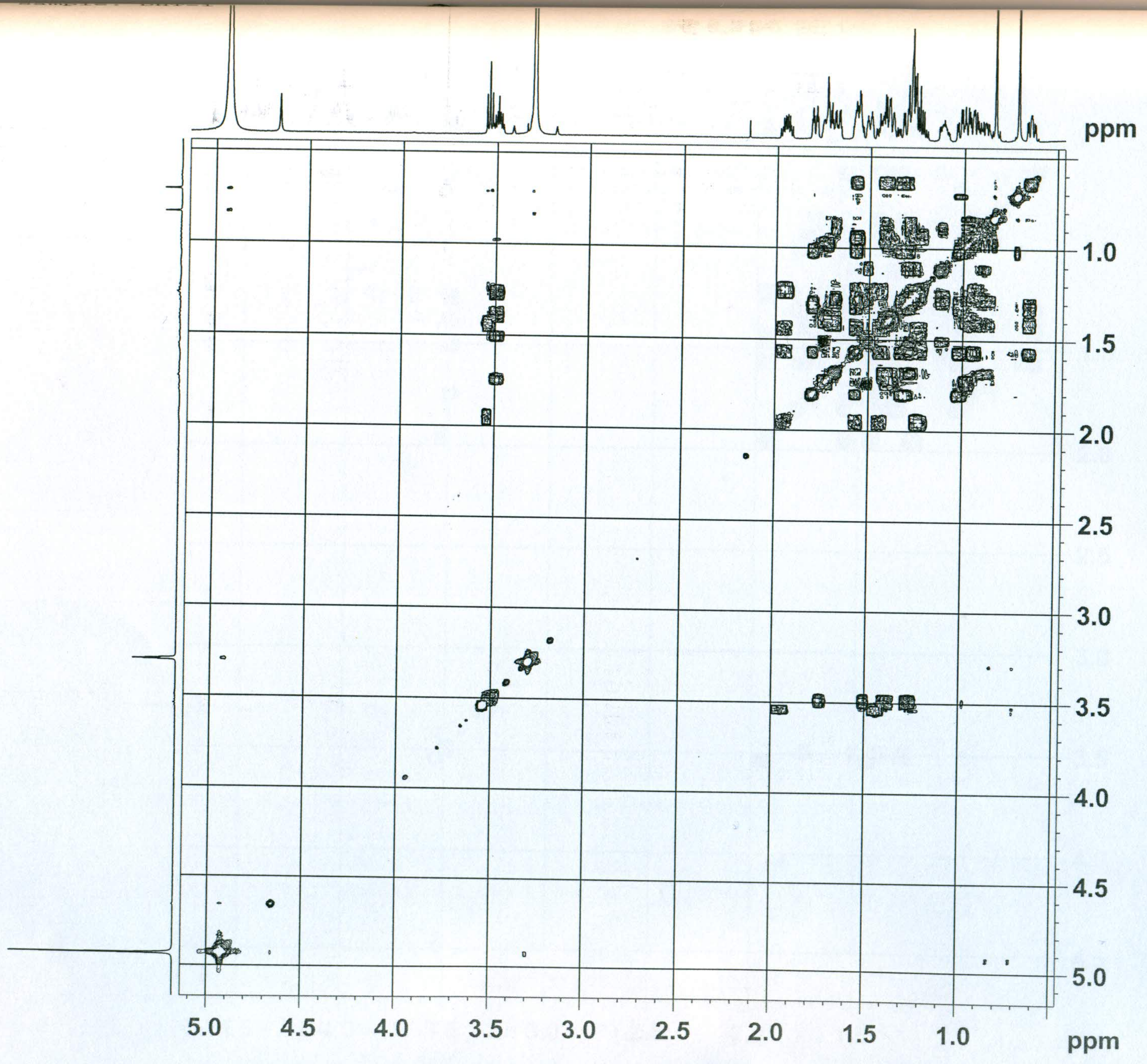

BRUKER

ANCE AV 600
LAB. No. 108

NAME	FEB06
EXPNO	2
PROCNO	1
Date_	20090206
Time	9.18
INSTRUM	spect
PROBHD	5 mm CPTCI 1H-
PULPROG	cosygpqf
TD	2048
SOLVENT	MeOD
NS	8
DS	4
SWH	3180.662 Hz
FIDRES	1.553057 Hz
AQ	0.3221528 sec
RG	181
DW	157.200 usec
DE	6.50 usec
TE	293.0 K
D0	0.00000300 sec
D1	1.48689198 sec
D13	0.00000400 sec
D16	0.00015000 sec
IN0	0.00031440 sec

======== CHANNEL f1 ========

NUC1	1H
P0	7.40 usec
P1	7.40 usec
PL1	3.30 dB
PL1W	9.16420078 W
SFO1	600.2318007 MHz

====== GRADIENT CHANNEL =====

GPNAM1	SINE.100
GPZ1	10.00 %
P16	2000.00 usec
ND0	1
TD	256
SFO1	600.2318 MHz
FIDRES	12.424461 Hz
SW	5.299 ppm
FnMODE	QF
SI	1024
SF	600.2300157 MHz
WDW	QSINE
SSB	0
LB	0.00 Hz
GB	0
PC	1.40
SI	1024
MC2	QF
SF	600.2300157 MHz
WDW	QSINE
SSB	0
LB	0.00 Hz
GB	0

Sample: DHT=1

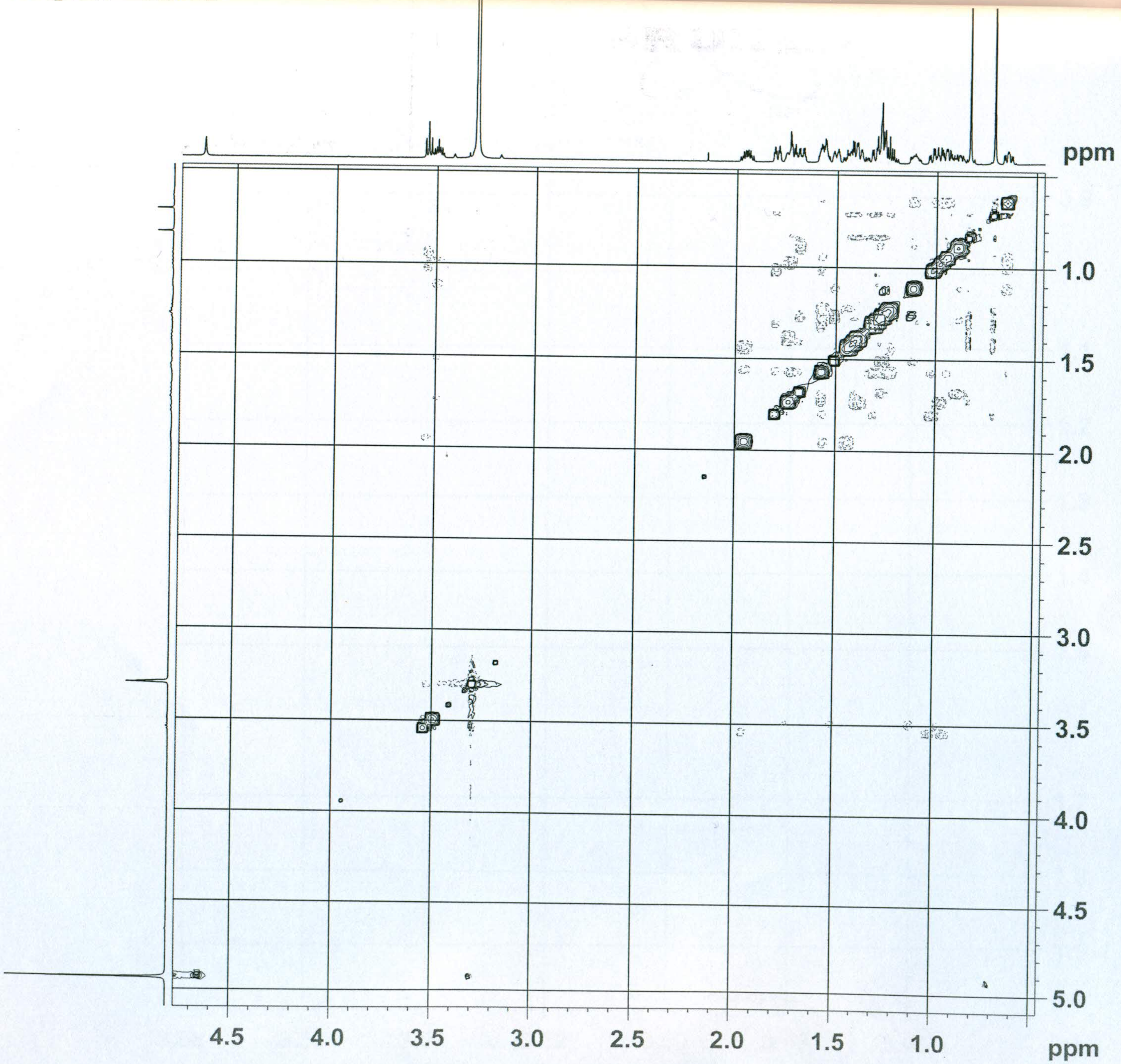

NAME	FEB06
EXPNO	3
PROCNO	1
Date_	20090206
Time	10.22
INSTRUM	spect
PROBHD	5 mm CPTCI 1H-
PULPROG	noesygpph
TD	1024
SOLVENT	MeOD
NS	8
DS	4
SWH	3180.662 Hz
FIDRES	3.106115 Hz
AQ	0.1611800 sec
RG	90.5
DW	157.200 usec
DE	6.50 usec
TE	293.0 K
D0	0.00014778 sec
D1	2.00000000 sec
D8	0.80000001 sec
D16	0.00015000 sec
IN0	0.00031440 sec

======== CHANNEL f1 ========

NUC1	1H
P1	7.40 usec
P2	14.80 usec
PL1	3.30 dB
PL1W	9.16420078 W
SFO1	600.2318007 MHz

====== GRADIENT CHANNEL =====

GPNAM1	SINE.100
GPNAM2	SINE.100
GPZ1	40.00 %
GPZ2	-40.00 %
P16	2000.00 usec
ND0	1
TD	256
SFO1	600.2318 MHz
FIDRES	12.424461 Hz
SW	5.299 ppm
FnMODE	States-TPPI
SI	1024
SF	600.2300157 MHz
WDW	QSINE
SSB	2
LB	0.00 Hz
GB	0
PC	1.40
SI	1024
MC2	States-TPPI
SF	600.2300157 MHz
WDW	QSINE
SSB	2
LB	0.00 Hz
GB	0
