# Supplementary material for: Molecular docking simulation studies on potent butyrylcholinesterase inhibitors obtained from microbial transformation of dihydrotestosterone
Source: Chem Cent J. 2013 Oct 8;7:164. doi: 10.1186/1752-153X-7-164 (PMC4126177; doi:10.1186/1752-153X-7-164)
Supplement: Additional file 2 — 1H-, 13C- and 2D-NMR spectra of compound 3. [file 1752-153X-7-164-S2.pdf]

AVANCE 400-B
LAB. No. 109

SALMAN ZAFAR/DR.IQBAL/DTC-B2/CD3OD

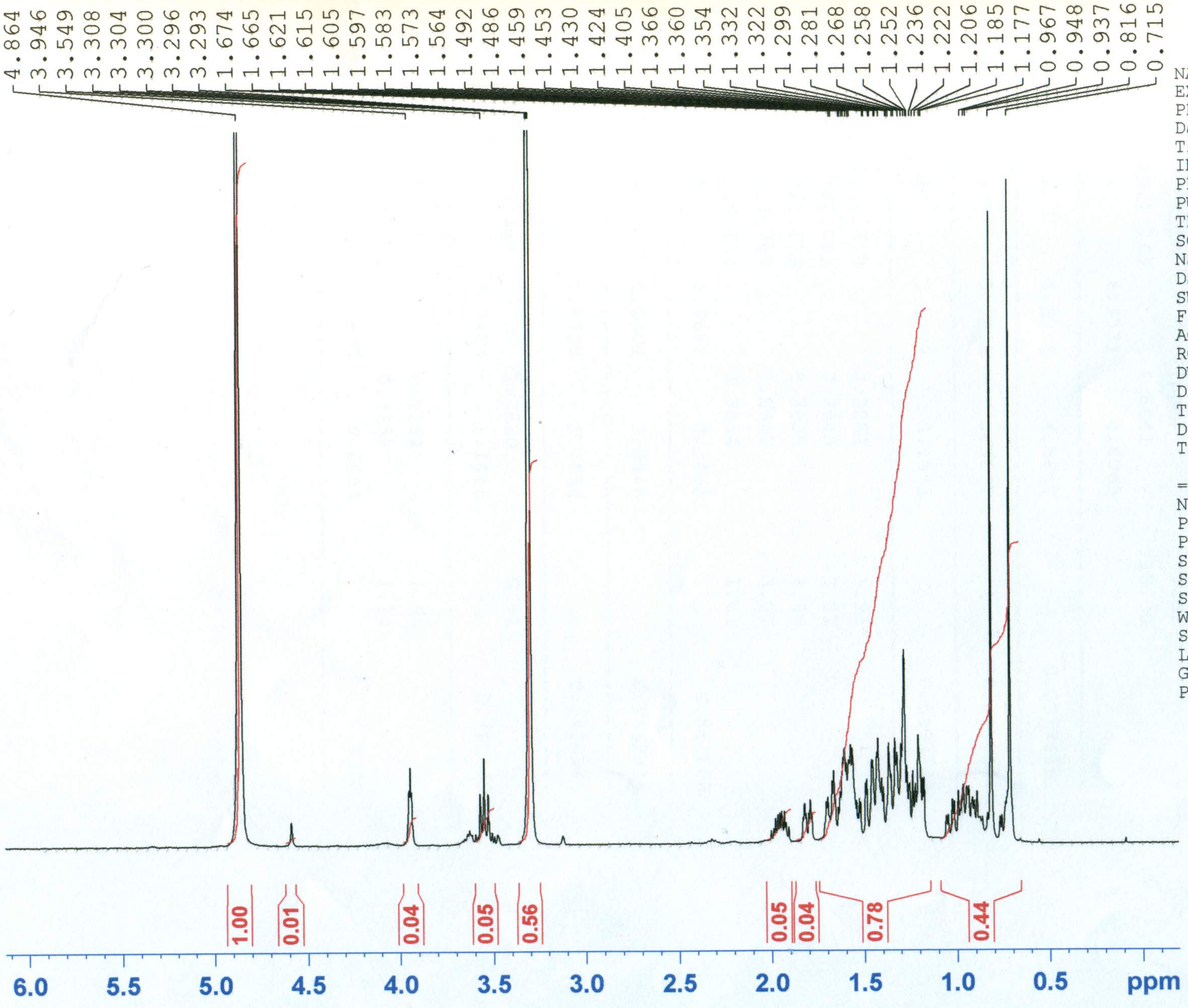

NAME	feb11
EXPNO	2
PROCNO	1
Date_	20090211
Time	10.42
INSTRUM	spect
PROBHD	5 mm DUL 13C-1
PULPROG	zg30
TD	32768
SOLVENT	MeOD
NS	128
DS	0
SWH	8012.820 Hz
FIDRES	0.244532 Hz
AQ	2.0447731 sec
RG	456.1
DW	62.400 usec
DE	6.50 usec
TE	300.0 K
D1	1.00000000 sec
TD0	1

======== CHANNEL f1 ========

NUC1	1H
P1	11.90 usec
PL1	-2.00 dB
SFO1	400.3332026 MHz
SI	16384
SF	400.3300099 MHz
WDW	EM
SSB	0
LB	0.30 Hz
GB	0
PC	1.00

Salman/Dr,Iqbal/BB/
Cd3od/DTC-B2/

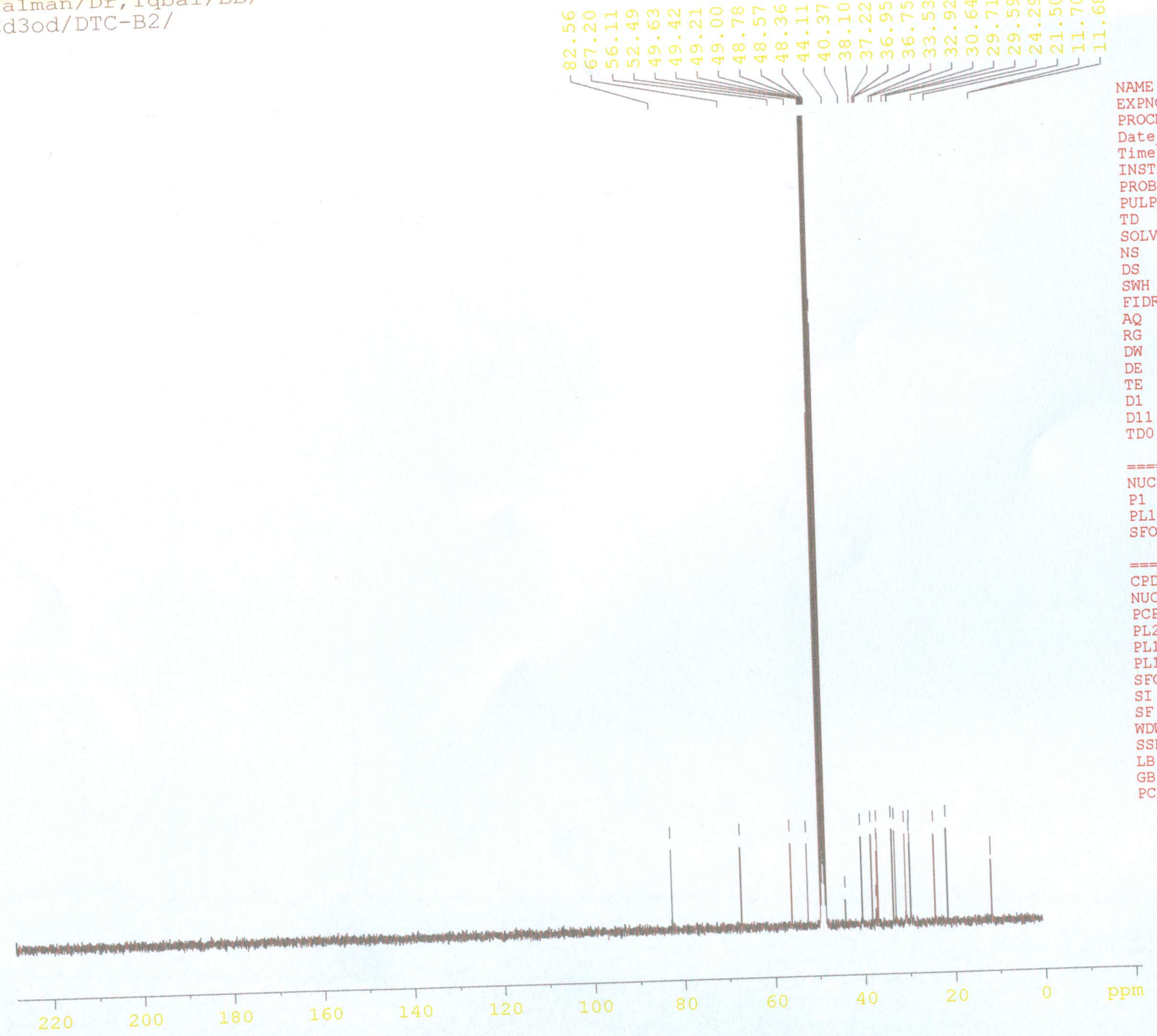

NAME	feb25
EXPNO	11
PROCNO	1
Date_	20090225
Time	15.50
INSTRUM	spect
PROBHD	5 mm Dual 13C/
PULPROG	zgpg
TD	32768
SOLVENT	MeOD
NS	27648
DS	2
SWH	24154.590 Hz
FIDRES	0.737140 Hz
AQ	0.6783476 sec
RG	32768
DW	20.700 usec
DE	6.50 usec
TE	298.6 K
D1	1.50000000 sec
D11	0.03000000 sec
TD0	27

======== CHANNEL f1 ========

NUC1	13C
P1	14.20 usec
PL1	3.00 dB
SFO1	100.6498899 MHz

======== CHANNEL f2 ========

CPDPRG2	waltz16
NUC2	1H
PCPD2	100.00 usec
PL2	4.00 dB
PL12	25.83 dB
PL13	25.83 dB
SFO2	400.2320011 MHz
SI	16384
SF	100.6377708 MHz
WDW	EM
SSB	0
LB	1.00 Hz
GB	0
PC	1.40

400-A No. 109

Salman/Dr,Iqbal/Cd3od/
Dept-135/DTC-B2

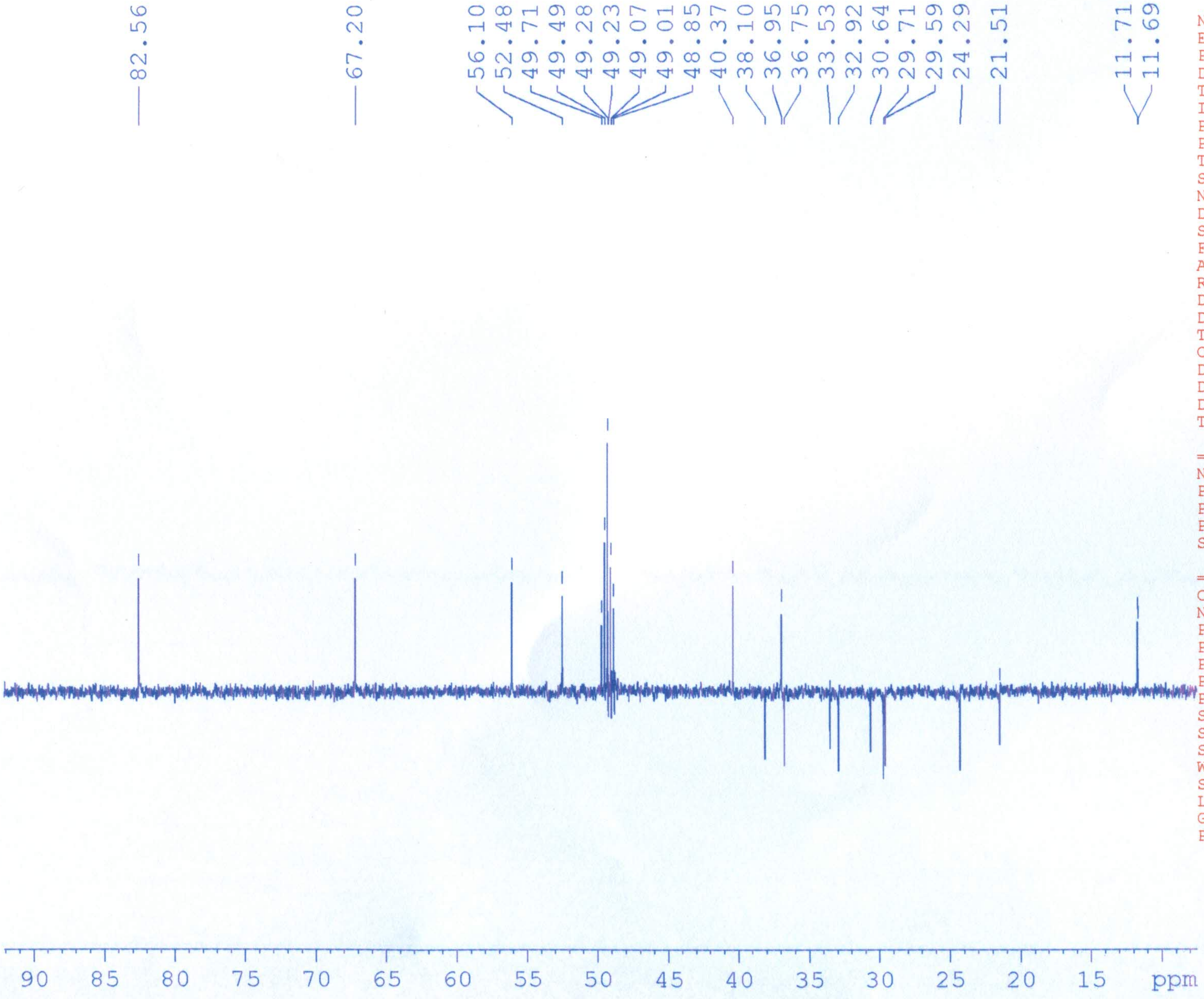

NAME	feb26
EXPNO	7
PROCNO	1
Date_	20090226
Time	11.59
INSTRUM	spect
PROBHD	5 mm Dual 13C/
PULPROG	dept135
TD	32768
SOLVENT	MeOD
NS	20480
DS	2
SWH	19157.088 Hz
FIDRES	0.584628 Hz
AQ	0.8552948 sec
RG	16384
DW	26.100 usec
DE	6.50 usec
TE	298.0 K
CNST2	145.0000000
D1	1.50000000 sec
D2	0.00344828 sec
D12	0.00002000 sec
TD0	20

======== CHANNEL f1 ========

NUC1	13C
P1	14.20 usec
P2	28.40 usec
PL1	3.00 dB
SFO1	100.6474746 MHz

======== CHANNEL f2 ========

CPDPRG2	waltz16
NUC2	1H
P3	8.10 usec
P4	16.20 usec
PCPD2	100.00 usec
PL2	4.00 dB
PL12	25.83 dB
SFO2	400.2320011 MHz
SI	16384
SF	100.6377708 MHz
WDW	EM
SSB	0
LB	1.00 Hz
GB	0
PC	1.40

400-A
No. 109

DTC-B2/Dept-90/

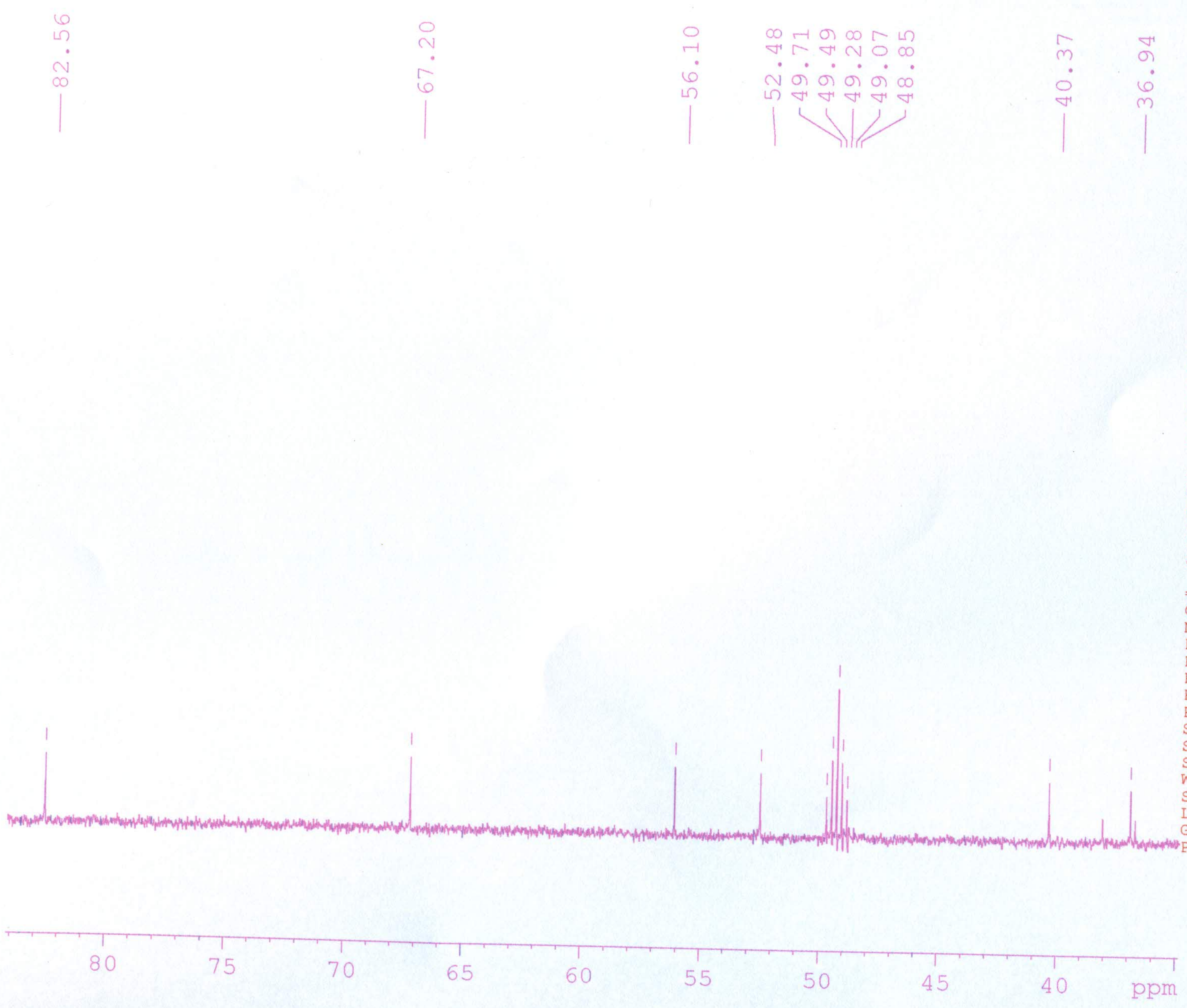

400-A
No. 109

NAME	feb26
EXPNO	8
PROCNO	1
Date_	20090227
Time	1.38
INSTRUM	spect
PROBHD	5 mm Dual 13C/
PULPROG	dept90
TD	32768
SOLVENT	MeOD
NS	8192
DS	2
SWH	19157.088 Hz
FIDRES	0.584628 Hz
AQ	0.8552948 sec
RG	16384
DW	26.100 usec
DE	6.50 usec
TE	297.5 K
CNST2	145.0000000
D1	1.50000000 sec
D2	0.00344828 sec
D12	0.00002000 sec
TD0	8

======== CHANNEL f1 ========

NUC1	13C
P1	14.20 usec
P2	28.40 usec
PL1	3.00 dB
SFO1	100.6474746 MHz

======== CHANNEL f2 ========

CPDPRG2	waltz16
NUC2	1H
P3	8.10 usec
P4	16.20 usec
PCPD2	100.00 usec
PL2	4.00 dB
PL12	25.83 dB
SFO2	400.2320011 MHz
SI	16384
SF	100.6377708 MHz
WDW	EM
SSB	0
LB	1.00 Hz
GB	0
PC	1.40

[illegible]
Sample: DTC=B2

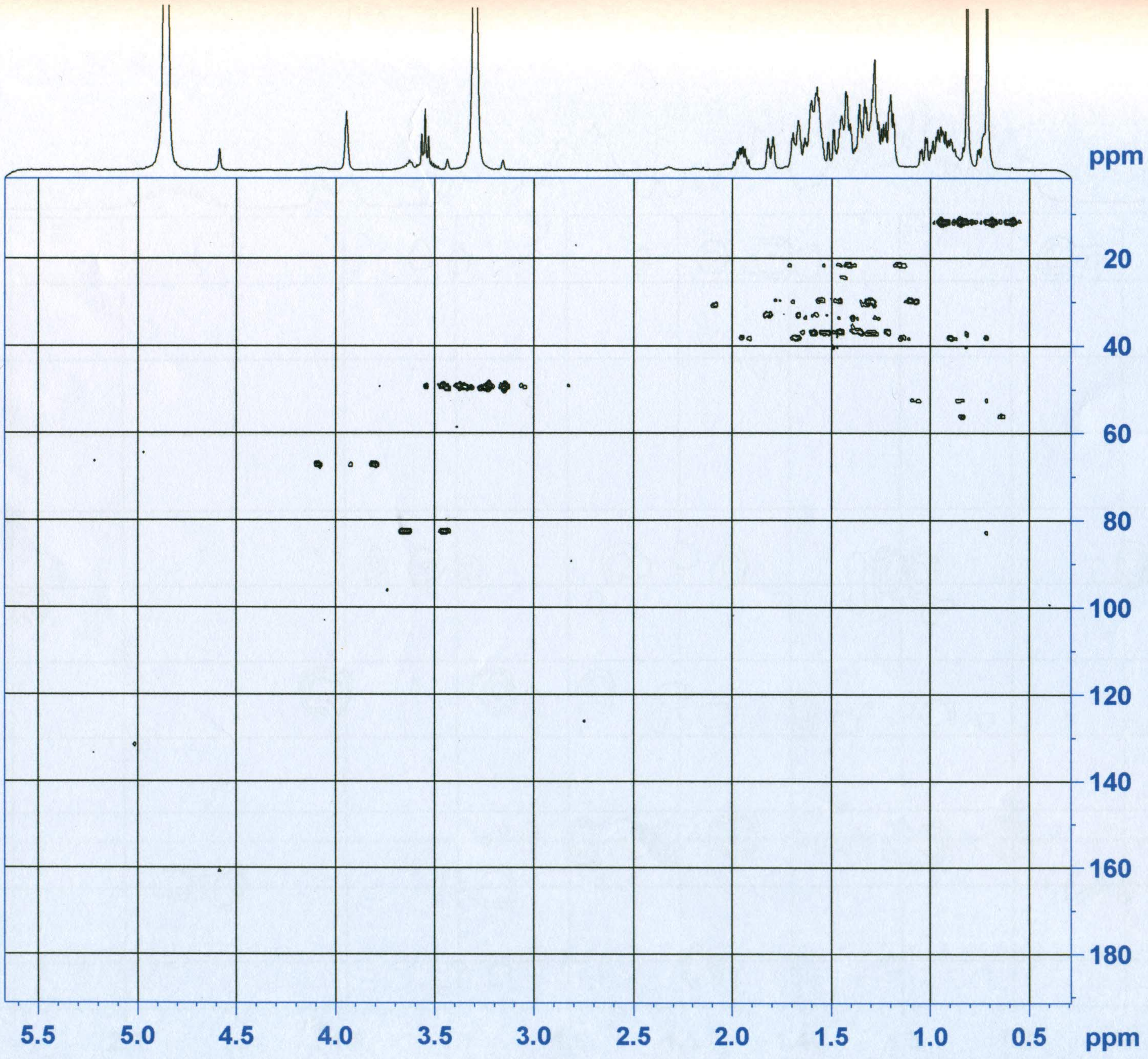

BRUKER

NAME	FEB24	
EXPNO	4	
PROCNO	1	
Date_	20090224	
Time	15.49	
INSTRUM	spect	
PROBHD	5 mm TXI 1H-13	
PULPROG	hmqcgpqf	
TD	1024	
SOLVENT	MeOD	
NS	32	
DS	8	
SWH	2693.966	Hz
FIDRES	2.630826	Hz
AQ	0.1902900	sec
RG	32768	
DW	185.600	usec
DE	6.50	usec
TE	296.9	K
CNST2	145.0000000	
D0	0.00000300	sec
D1	1.50000000	sec
D2	0.00344828	sec
D12	0.00002000	sec
D13	0.00000400	sec
D16	0.00015000	sec
IN0	0.00002090	sec

======== CHANNEL f1 ========

NUC1	1H	
P1	7.40	usec
P2	14.80	usec
PL1	3.00	dB
SFO1	500.2315007	MHz

======== CHANNEL f2 ========

CPDPRG2	garp	
NUC2	13C	
P3	12.70	usec
PCPD2	100.00	usec
PL2	-4.00	dB
PL12	25.00	dB
SFO2	125.7948834	MHz

====== GRADIENT CHANNEL =====

GPNAM1	SINE.100	
GPNAM2	SINE.100	
GPNAM3	SINE.100	
GPX1	0.00	%
GPX2	0.00	%
GPX3	0.00	%
GPY1	0.00	%
GPY2	0.00	%
GPY3	0.00	%
GPZ1	50.00	%
GPZ2	30.00	%
GPZ3	40.10	%
P16	1500.00	usec
ND0	2	
TD	256	
SFO1	125.7949	MHz
FIDRES	93.363388	Hz
SW	190.000	ppm
FnMODE	QF	
SI	1024	
SF	500.2300120	MHz
WDW	SINE	
SSB	0	
LB	0.00	Hz
GB	0	
PC	1.00	
SI	512	
MC2	QF	
SF	125.7827554	MHz
WDW	SINE	
SSB	0	
LB	0.00	Hz
GB	0	

[illegible] 500
LAB. No. 118

[illegible]
Sample: DTC=B2

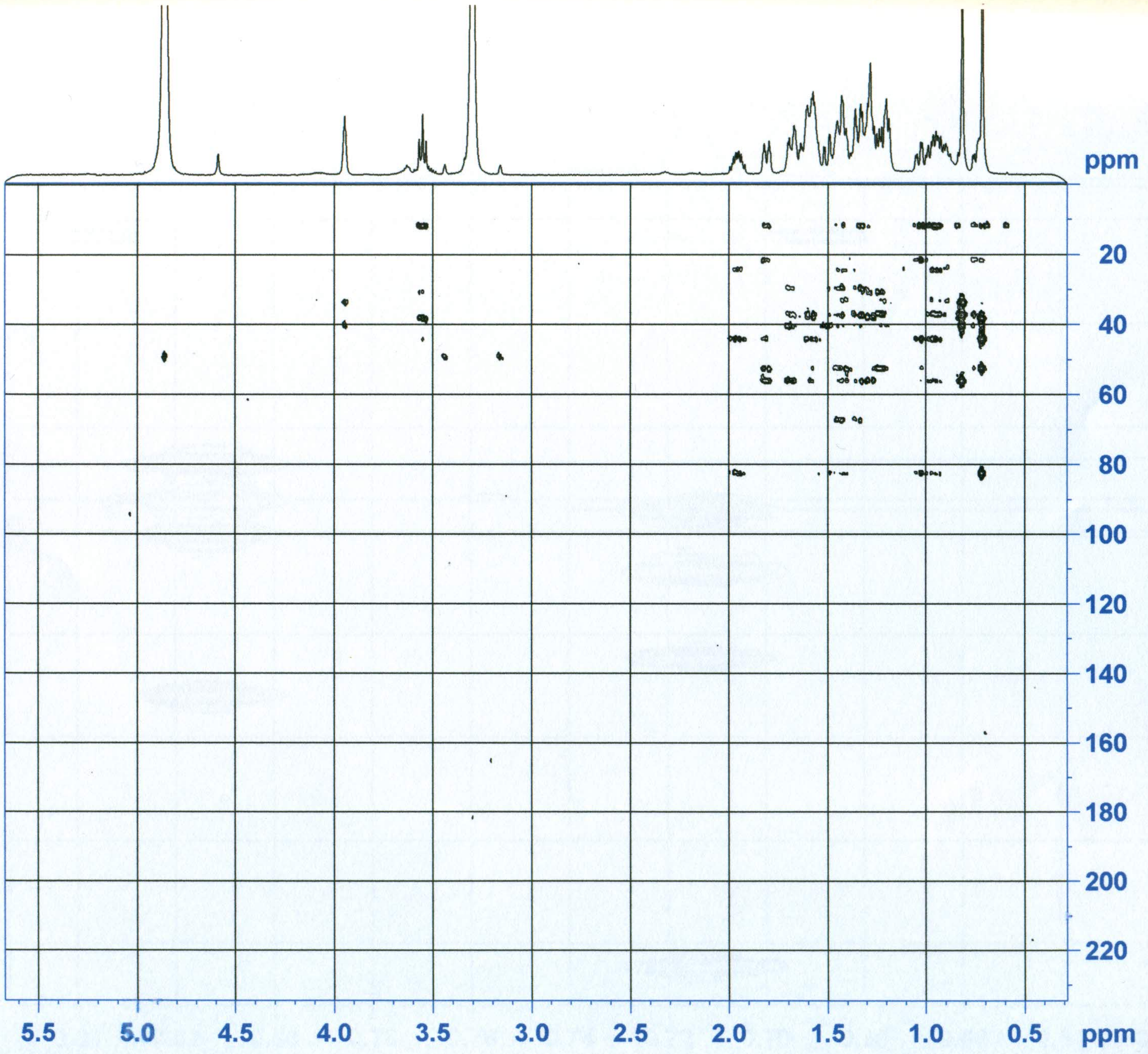

BRUKER

NAME	FEB24	
EXPNO	5	
PROCNO	1	
Date_	20090224	
Time_	19.44	
INSTRUM	spect	
PROBHD	5 mm TXI 1H-13	
PULPROG	hmbcgplpndqf	
TD	2048	
SOLVENT	MeOD	
NS	64	
DS	8	
SWH	2693.966	Hz
FIDRES	1.315413	Hz
AQ	0.3803444	sec
RG	32768	
DW	185.600	usec
DE	6.50	usec
TE	297.1	K
CNST2	145.0000000	
CNST13	10.0000000	
D0	0.00000300	sec
D1	1.50000000	sec
D2	0.00344828	sec
D6	0.05000000	sec
D16	0.00015000	sec
IN0	0.00001690	sec
======== CHANNEL f1 ========		
NUC1	1H	
P1	7.40	usec
P2	14.80	usec
PL1	3.00	dB
SFO1	500.2315007	MHz
======== CHANNEL f2 ========		
NUC2	13C	
P3	12.70	usec
PL2	-4.00	dB
SFO2	125.7974871	MHz
====== GRADIENT CHANNEL =====		
GPNAM1	SINE.100	
GPNAM2	SINE.100	
GPNAM3	SINE.100	
GPX1	0.00	%
GPX2	0.00	%
GPX3	0.00	%
GPY1	0.00	%
GPY2	0.00	%
GPY3	0.00	%
GPZ1	50.00	%
GPZ2	30.00	%
GPZ3	40.10	%
P16	1500.00	usec
ND0	2	
TD	256	
SFO1	125.7975	MHz
FIDRES	115.478165	Hz
SW	235.000	ppm
FnMODE	QF	
SI	2048	
SF	500.2300120	MHz
WDW	SINE	
SSB	0	
LB	0.00	Hz
GB	0	
PC	1.00	
SI	512	
MC2	QF	
SF	125.7827554	MHz
WDW	SINE	
SSB	0	
LB	0.00	Hz
GB	0	

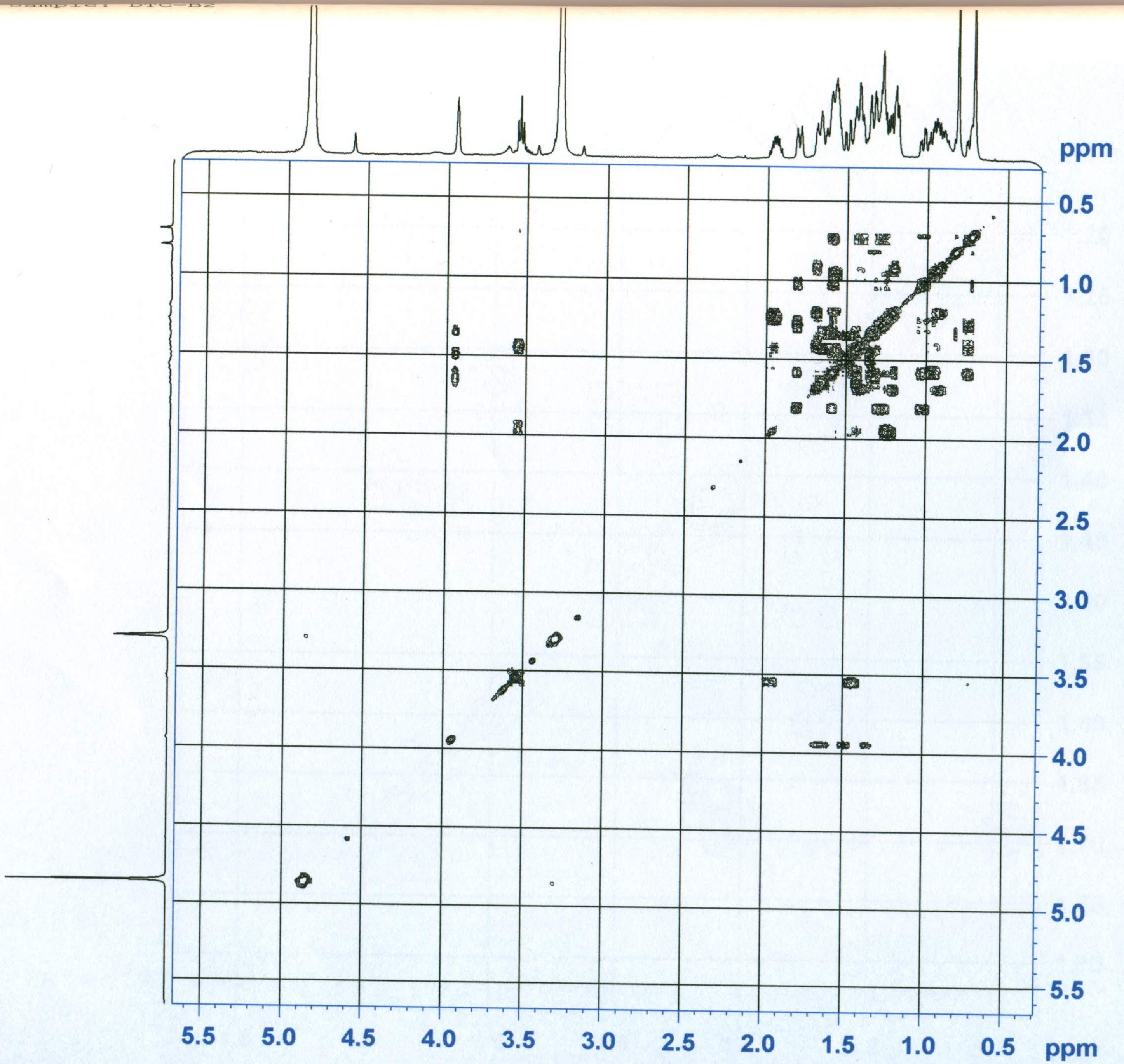

BRUKER

LAB. No. 118

NAME	FEB24
EXPNO	2
PROCNO	1
Date_	20090224
Time	11.16
INSTRUM	spect
PROBHD	5 mm TXI 1H-13
PULPROG	cosygpqf
TD	2048
SOLVENT	MeOD
NS	8
DS	8
SWH	2693.966 Hz
FIDRES	1.315413 Hz
AQ	0.3803444 sec
RG	812.7
DW	185.600 usec
DE	6.50 usec
TE	297.3 K
D0	0.00000300 sec
D1	1.48689198 sec
D13	0.00000400 sec
D16	0.00015000 sec
IN0	0.00037120 sec
======== CHANNEL f1 ========	
NUC1	1H
P0	7.40 usec
P1	7.40 usec
PL1	3.00 dB
SFO1	500.2315007 MHz
====== GRADIENT CHANNEL =====	
GPNAM1	SINE.100
GPX1	0.00 %
GPY1	0.00 %
GPZ1	10.00 %
P16	1500.00 usec
ND0	1
TD	256
SFO1	500.2315 MHz
FIDRES	10.523305 Hz
SW	5.385 ppm
FnMODE	QF
SI	1024
SF	500.2300120 MHz
WDW	QSINE
SSB	0
LB	0.00 Hz
GB	0
PC	1.00
SI	1024
MC2	QF
SF	500.2300120 MHz
WDW	QSINE
SSB	0
LB	0.00 Hz
GB	0

Salman/Prof.Iqbal
Sample:DTC=B2

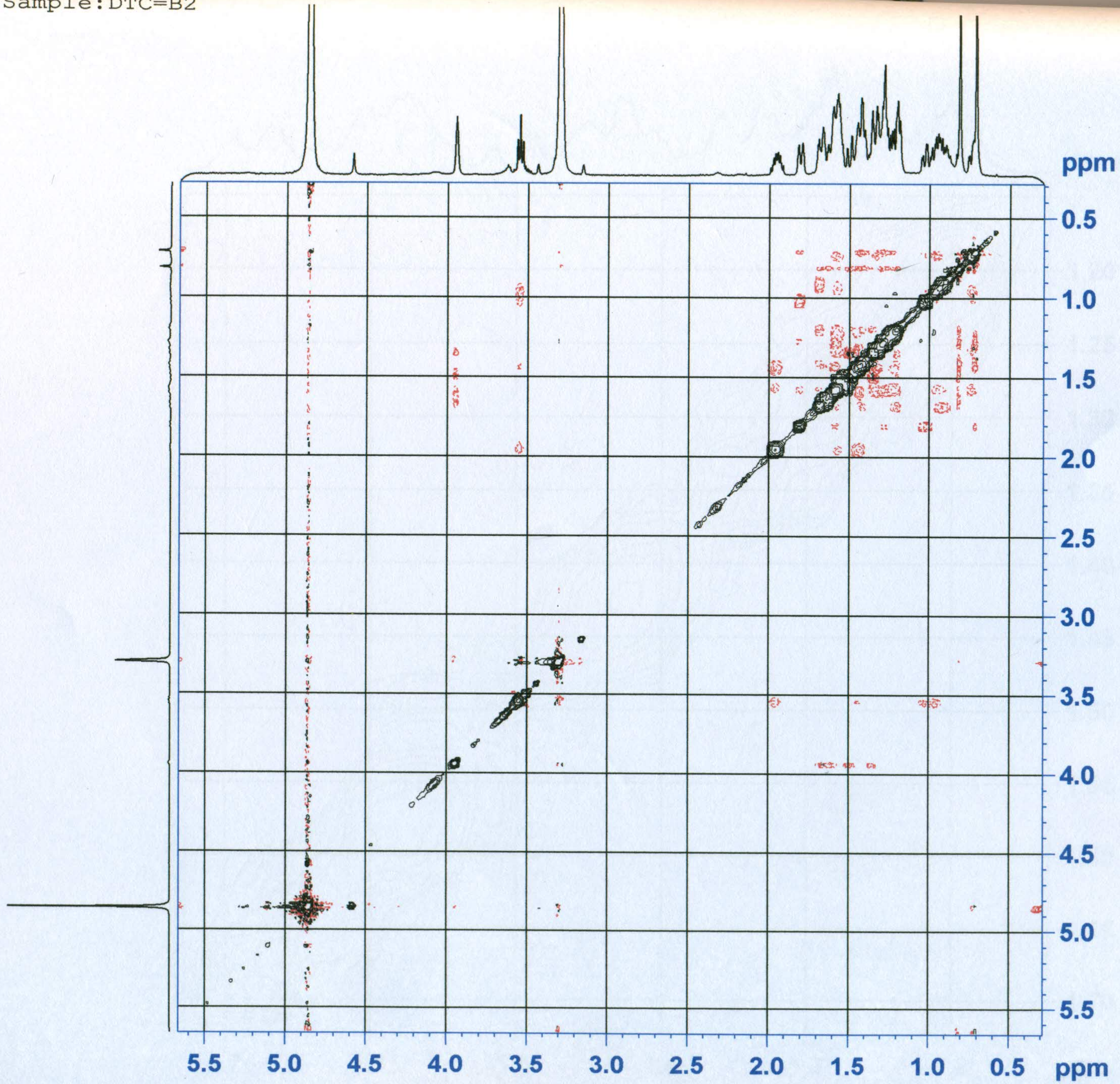

BRUKER

NAME	FEB24	
EXPNO	3	
PROCNO	1	
Date_	20090224	
Time	12.23	
INSTRUM	spect	
PROBHD	5 mm TXI 1H-13	
PULPROG	noesygpph	
TD	1024	
SOLVENT	MeOD	
NS	16	
DS	8	
SWH	2693.966	Hz
FIDRES	2.630826	Hz
AQ	0.1902900	sec
RG	574.7	
DW	185.600	usec
DE	6.50	usec
TE	297.4	K
D0	0.00017618	sec
D1	2.00000000	sec
D8	0.80000001	sec
D16	0.00015000	sec
IN0	0.00037120	sec

======== CHANNEL f1 ========

NUC1	1H	
P1	7.40	usec
P2	14.80	usec
PL1	3.00	dB
SFO1	500.2315007	MHz

====== GRADIENT CHANNEL =====

GPNAM1	SINE.100	
GPNAM2	SINE.100	
GPX1	0.00	%
GPX2	0.00	%
GPY1	0.00	%
GPY2	0.00	%
GPZ1	40.00	%
GPZ2	-40.00	%
P16	1500.00	usec
ND0	1	
TD	256	
SFO1	500.2315	MHz
FIDRES	10.523305	Hz
SW	5.385	ppm
FnMODE	States-TPPI	
SI	1024	
SF	500.2300120	MHz
WDW	QSINE	
SSB	2	
LB	0.00	Hz
GB	0	
PC	1.00	
SI	1024	
MC2	States-TPPI	
SF	500.2300120	MHz
WDW	QSINE	
SSB	2	
LB	0.00	Hz
GB	0	

AVANCE 500
LAB. No. 118
