# Supplementary material for: Molecular docking simulation studies on potent butyrylcholinesterase inhibitors obtained from microbial transformation of dihydrotestosterone
Source: Chem Cent J. 2013 Oct 8;7:164. doi: 10.1186/1752-153X-7-164 (PMC4126177; doi:10.1186/1752-153X-7-164)
Supplement: Additional file 3 — 1H-, 13C- and 2D-NMR spectra of compound 4. [file 1752-153X-7-164-S3.pdf]

Salman/Dr,Iqbal/TGF-4/

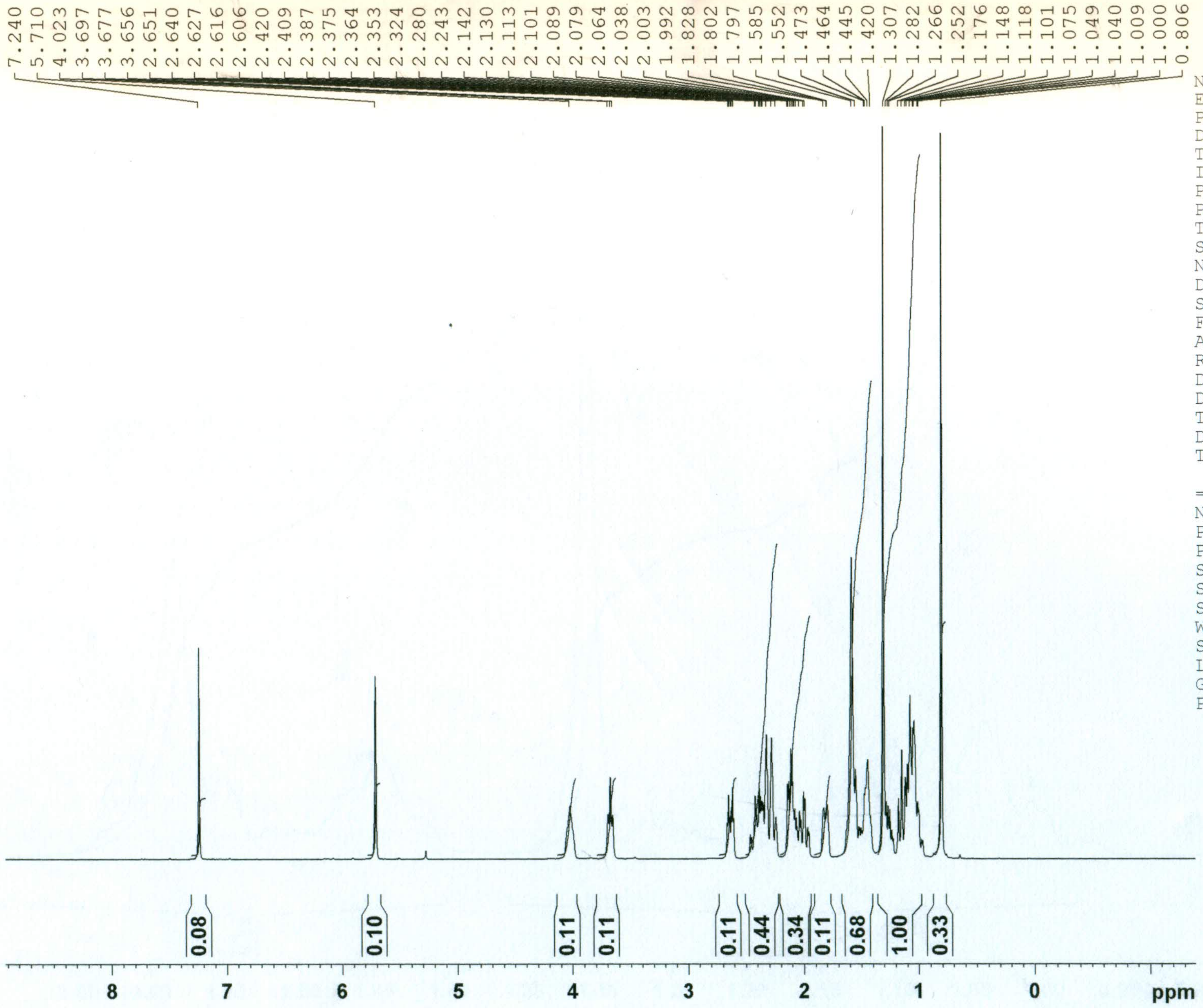

NAME	Oct09
EXPNO	3
PROCNO	1
Date_	20091009
Time	10.11
INSTRUM	spect
PROBHD	5 mm Dual 13C/
PULPROG	zg30
TD	32768
SOLVENT	CDCl3
NS	128
DS	0
SWH	8012.820 Hz
FIDRES	0.244532 Hz
AQ	2.0447731 sec
RG	181
DW	62.400 usec
DE	6.50 usec
TE	300.1 K
D1	1.00000000 sec
TD0	1

======== CHANNEL f1 ========

NUC1	1H
P1	8.10 usec
PL1	4.00 dB
SFO1	400.2328016 MHz
SI	16384
SF	400.2300132 MHz
WDW	EM
SSB	0
LB	0.30 Hz
GB	0
PC	2.00

AVANCE 400-A
LAB. No. 109

[illegible]
Sample: TGF4

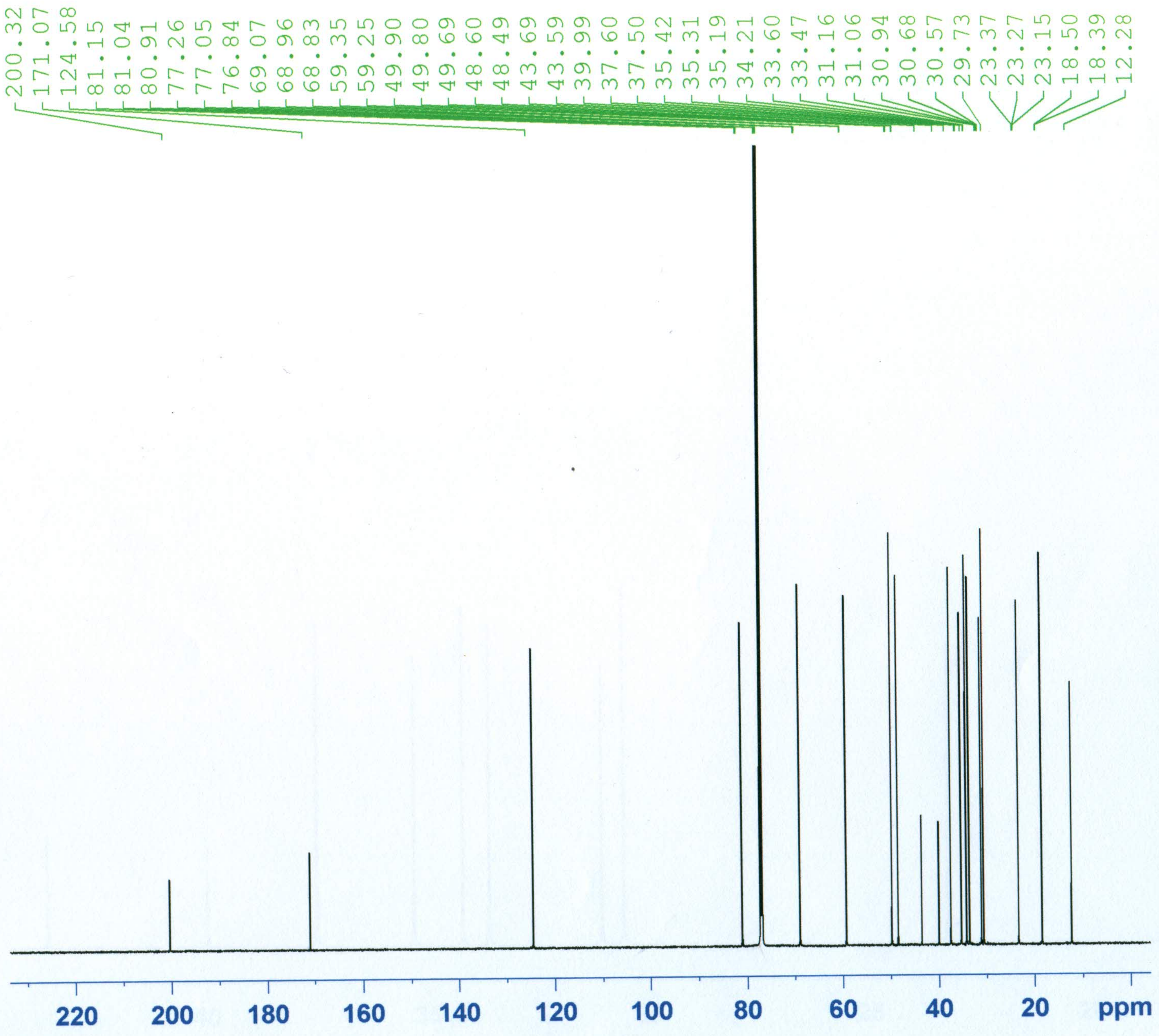

BRUKER

NAME	Oct 24	
EXPNO	6	
PROCNO	1	
Date_	20091025	
Time_	2.25	
INSTRUM	spect	
PROBHD	5 mm CPTCI 1H-	
PULPROG	zgpg	
TD	65536	
SOLVENT	CDCl3	
NS	12288	
DS	2	
SWH	35971.223	Hz
FIDRES	0.548877	Hz
AQ	0.9110143	sec
RG	32768	
DW	13.900	usec
DE	6.50	usec
TE	294.0	K
D1	1.50000000	sec
D11	0.03000000	sec
TD0	12	

======== CHANNEL f1 ========

NUC1	13C	
P1	16.00	usec
PL1	2.00	dB
PL1W	66.40702820	W
SFO1	150.9453107	MHz

======== CHANNEL f2 ========

CPDPRG2	waltz16	
NUC2	1H	
PCPD2	65.00	usec
PL2	3.30	dB
PL12	22.06	dB
PL13	27.00	dB
PL2W	9.16420078	W
PL12W	0.12192553	W
PL13W	0.03909260	W
SFO2	600.2336014	MHz
SI	32768	
SF	150.9279540	MHz
WDW	EM	
SSB	0	
LB	1.00	Hz
GB	0	
PC	1.40	

AVANCE AV 600
LAB. No. 108

Sample: TGF4

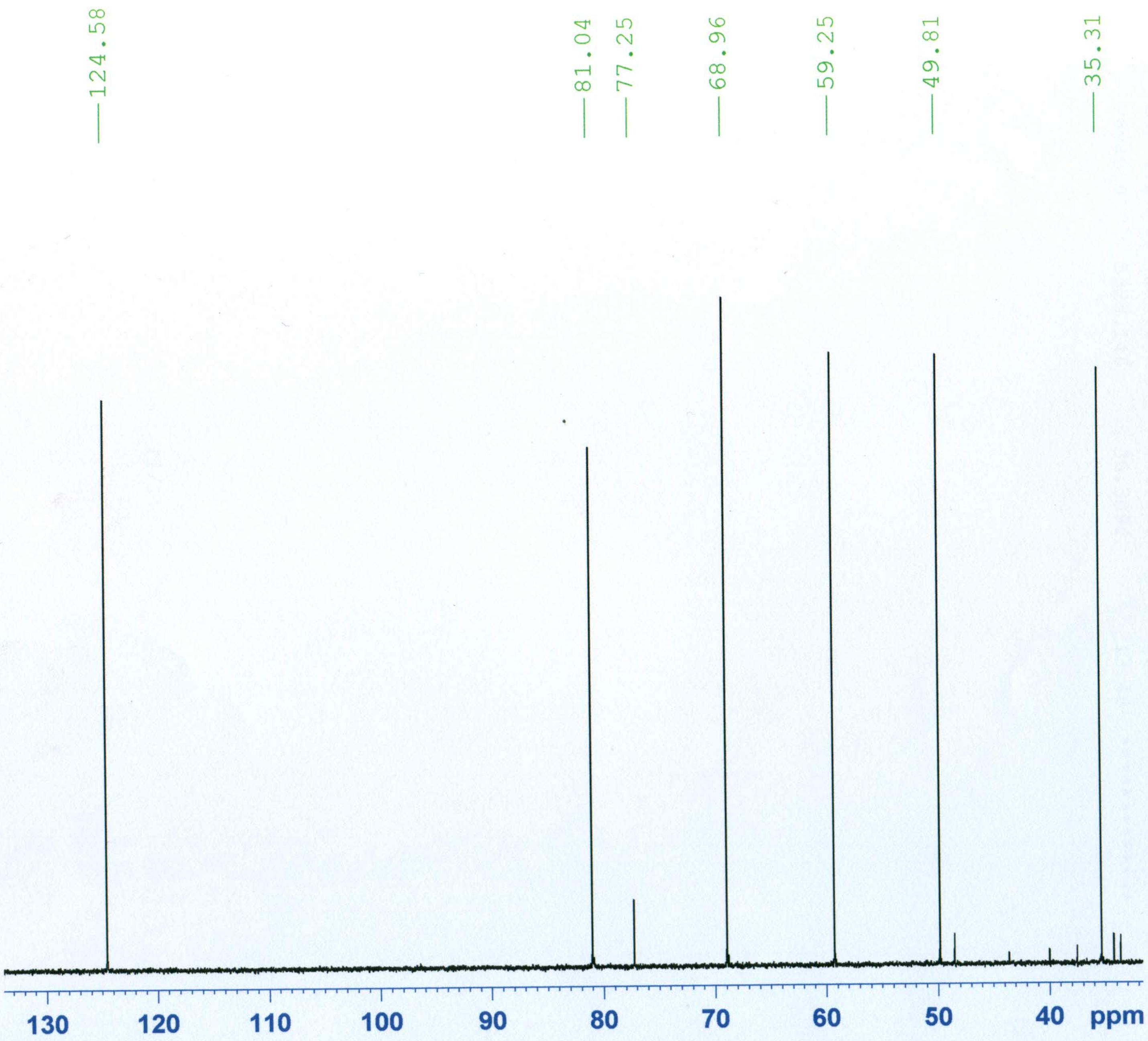

BRUKER

NAME	Oct 24
EXPNO	8
PROCNO	1
Date_	20091025
Time	15.24
INSTRUM	spect
PROBHD	5 mm CPTCI 1H-
PULPROG	deptsp90
TD	65536
SOLVENT	CDCl3
NS	1955
DS	2
SWH	30303.031 Hz
FIDRES	0.462388 Hz
AQ	1.0814105 sec
RG	32768
DW	16.500 usec
DE	6.50 usec
TE	294.9 K
CNST2	145.0000000
D1	1.50000000 sec
D2	0.00344828 sec
D12	0.00002000 sec
TD0	6

======== CHANNEL f1 ========

NUC1	13C
P1	16.00 usec
P12	2000.00 usec
PL0	120.00 dB
PL1	2.00 dB
PL0W	0.00000000 W
PL1W	66.40702820 W
SFO1	150.9430463 MHz
SP2	1.99 dB
SPNAM2	Crp60comp.4
SPOAL2	0.500
SPOFFS2	0.00 Hz

======== CHANNEL f2 ========

CPDPRG2	waltz16
NUC2	1H
P3	7.50 usec
P4	15.00 usec
PCPD2	65.00 usec
PL2	3.30 dB
PL12	22.06 dB
PL2W	9.16420078 W
PL12W	0.12192553 W
SFO2	600.2324009 MHz
SI	32768
SF	150.9279540 MHz
WDW	EM
SSB	0
LB	1.00 Hz
GB	0
PC	1.40

AVANCE AV 600
LAB. No. 108

Sample: TGF4

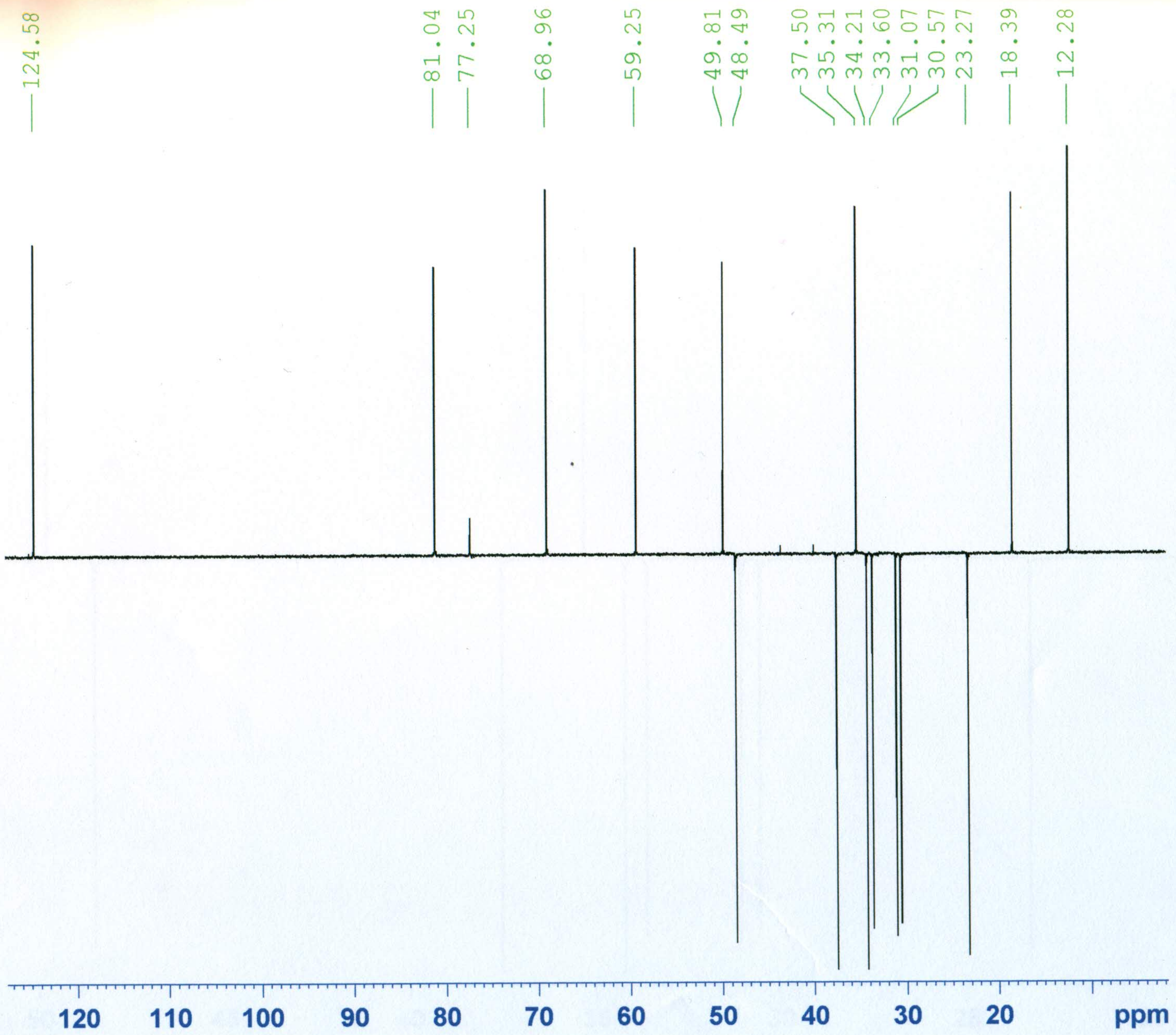

BRUKER

NAME	Oct 24	
EXPNO	7	
PROCNO	1	
Date_	20091025	
Time	10.54	
INSTRUM	spect	
PROBHD	5 mm CPTCI 1H-	
PULPROG	deptsp135	
TD	65536	
SOLVENT	CDCl3	
NS	6144	
DS	2	
SWH	30303.031	Hz
FIDRES	0.462388	Hz
AQ	1.0814105	sec
RG	32768	
DW	16.500	usec
DE	6.50	usec
TE	294.5	K
CNST2	145.0000000	
D1	1.50000000	sec
D2	0.00344828	sec
D12	0.00002000	sec
TD0	6	

======== CHANNEL f1 ========

NUC1	13C	
P1	16.00	usec
P12	2000.00	usec
PL0	120.00	dB
PL1	2.00	dB
PL0W	0.00000000	W
PL1W	66.40702820	W
SFO1	150.9430463	MHz
SP2	1.99	dB
SPNAM2	Crp60comp.4	
SPOAL2	0.500	
SPOFFS2	0.00	Hz

======== CHANNEL f2 ========

CPDPRG2	waltz16	
NUC2	1H	
P3	7.50	usec
P4	15.00	usec
PCPD2	65.00	usec
PL2	3.30	dB
PL12	22.06	dB
PL2W	9.16420078	W
PL12W	0.12192553	W
SFO2	600.2324009	MHz
SI	32768	
SF	150.9279540	MHz
WDW	EM	
SSB	0	
LB	1.00	Hz
GB	0	
PC	1.40	

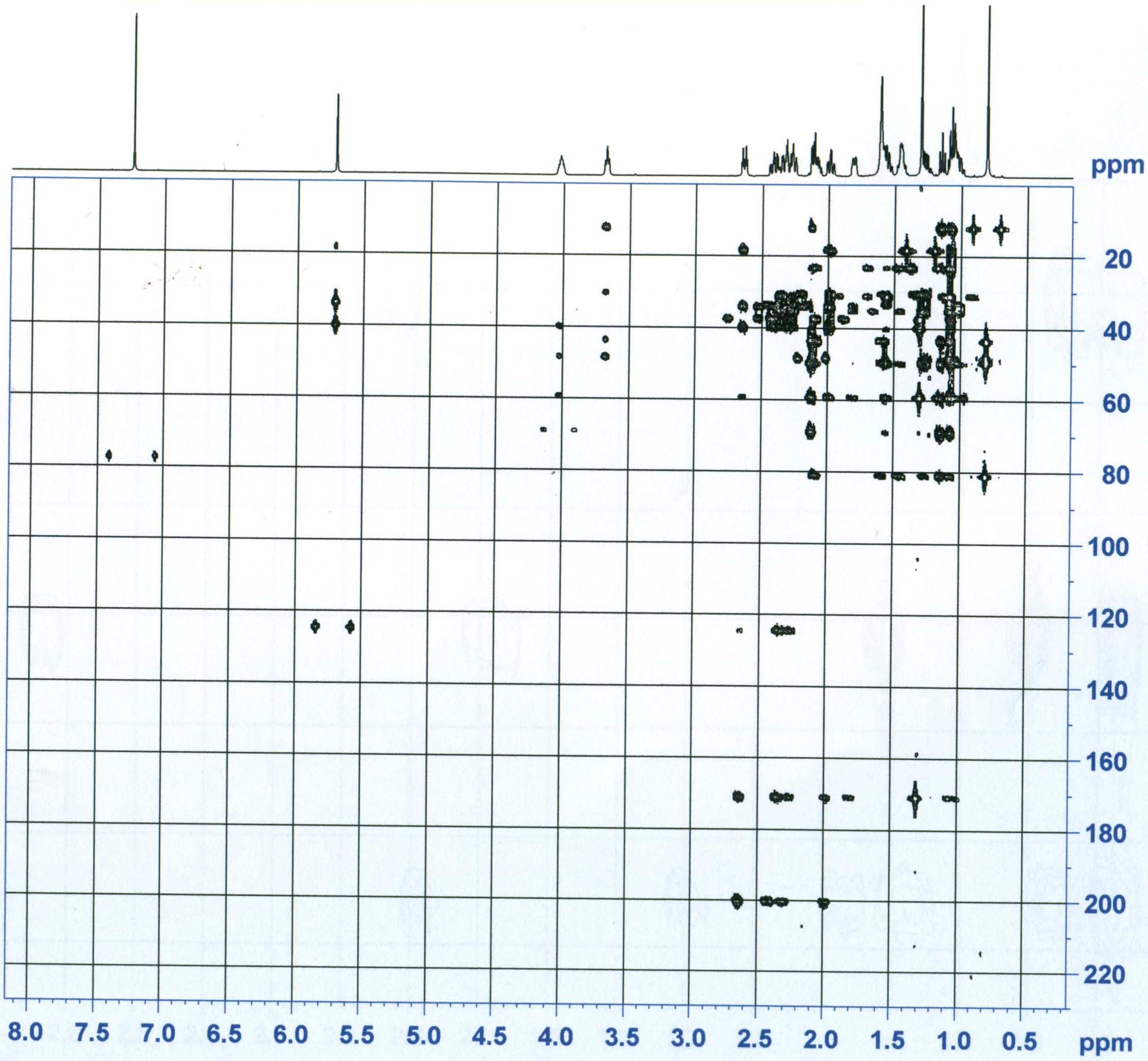

BRUKER

NAME	Oct 24
EXPNO	5
PROCNO	1
Date_	20091024
Time	22.13
INSTRUM	spect
PROBHD	5 mm CPTCI 1H-
PULPROG	hmbcgplpndqf
TD	4096
SOLVENT	CDCl3
NS	32
DS	8
SWH	4807.692 Hz
FIDRES	1.173753 Hz
AQ	0.4261380 sec
RG	46341
DW	104.000 usec
DE	6.50 usec
TE	294.2 K
CNST2	145.0000000
CNST13	13.0000000
D0	0.00000300 sec
D1	1.50000000 sec
D2	0.00344828 sec
D6	0.03846154 sec
D16	0.00015000 sec
IN0	0.00001440 sec

======== CHANNEL f1 ========

NUC1	1H
P1	7.40 usec
P2	14.80 usec
PL1	3.30 dB
PL1W	9.16420078 W
SFO1	600.2325210 MHz

======== CHANNEL f2 ========

NUC2	13C
P3	10.00 usec
PL2	2.00 dB
PL2W	66.40702820 W
SFO2	150.9453107 MHz

====== GRADIENT CHANNEL =====

GPNAM1	SINE.100
GPNAM2	SINE.100
GPNAM3	SINE.100
GPZ1	50.00 %
GPZ2	30.00 %
GPZ3	40.10 %
P16	2000.00 usec
ND0	2
TD	256
SFO1	150.9453 MHz
FIDRES	135.614929 Hz
SW	230.000 ppm
FnMODE	QF
SI	1024
SF	600.2300250 MHz
WDW	SINE
SSB	0
LB	0.00 Hz
GB	0
PC	1.40
SI	1024
MC2	QF
SF	150.9279540 MHz
WDW	SINE
SSB	0
LB	0.00 Hz
GB	0

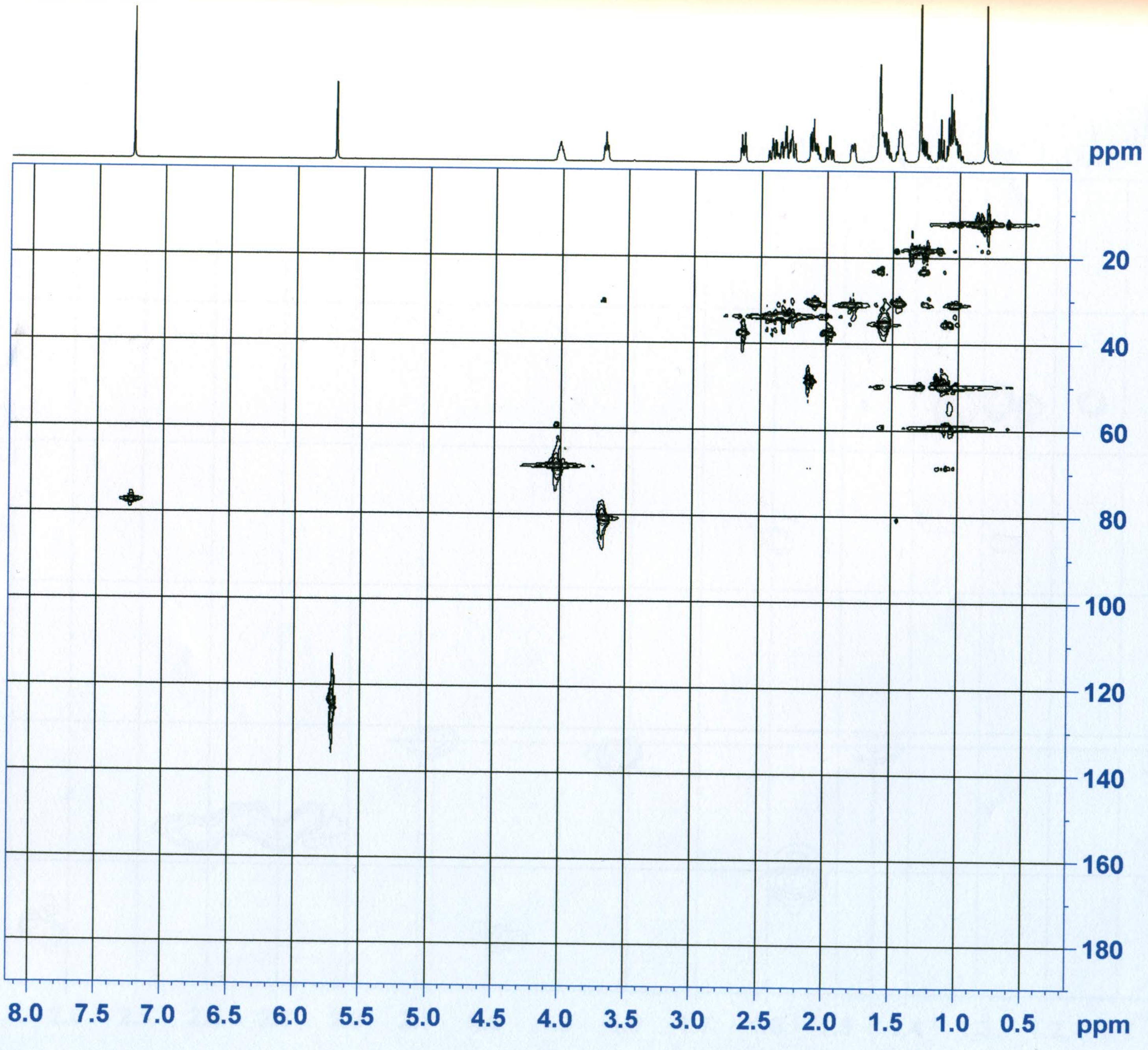

BRUKER

NAME	Oct 24	
EXPNO	4	
PROCNO	1	
Date_	20091024	
Time	18.29	
INSTRUM	spect	
PROBHD	5 mm CPTCI 1H-	
PULPROG	hsqcetgpsi	
TD	1024	
SOLVENT	CDCl3	
NS	32	
DS	8	
SWH	4807.692	Hz
FIDRES	4.695012	Hz
AQ	0.1066500	sec
RG	46341	
DW	104.000	usec
DE	6.50	usec
TE	294.4	K
CNST2	145.0000000	
D0	0.00000300	sec
D1	1.50000000	sec
D4	0.00172414	sec
D11	0.03000000	sec
D13	0.00000400	sec
D16	0.00015000	sec
D24	0.00110000	sec
IN0	0.00001745	sec
ZGOPTNS		

======== CHANNEL f1 ========

NUC1	1H	
P1	7.40	usec
P2	14.80	usec
P28	0.50	usec
PL1	3.30	dB
PL1W	9.16420078	W
SFO1	600.2325210	MHz

======== CHANNEL f2 ========

CPDPRG2	garp	
NUC2	13C	
P3	10.00	usec
P4	20.00	usec
PCPD2	60.00	usec
PL2	2.00	dB
PL12	17.56	dB
PL2W	66.40702820	W
PL12W	1.84592509	W
SFO2	150.9422922	MHz

====== GRADIENT CHANNEL =====

GPNAM1	SINE.100	
GPNAM2	SINE.100	
GPZ1	80.00	%
GPZ2	20.10	%
P16	2000.00	usec
ND0	2	
TD	256	
SFO1	150.9423	MHz
FIDRES	112.027481	Hz
SW	190.000	ppm
FnMODE	Echo-Antiecho	
SI	1024	
SF	600.2300250	MHz
WDW	QSINE	
SSB	2	
LB	0.00	Hz
GB	0	
PC	4.00	
SI	1024	
MC2	echo-antiecho	
SF	150.9279540	MHz
WDW	QSINE	
SSB	2	
LB	0.00	Hz
GB	0	

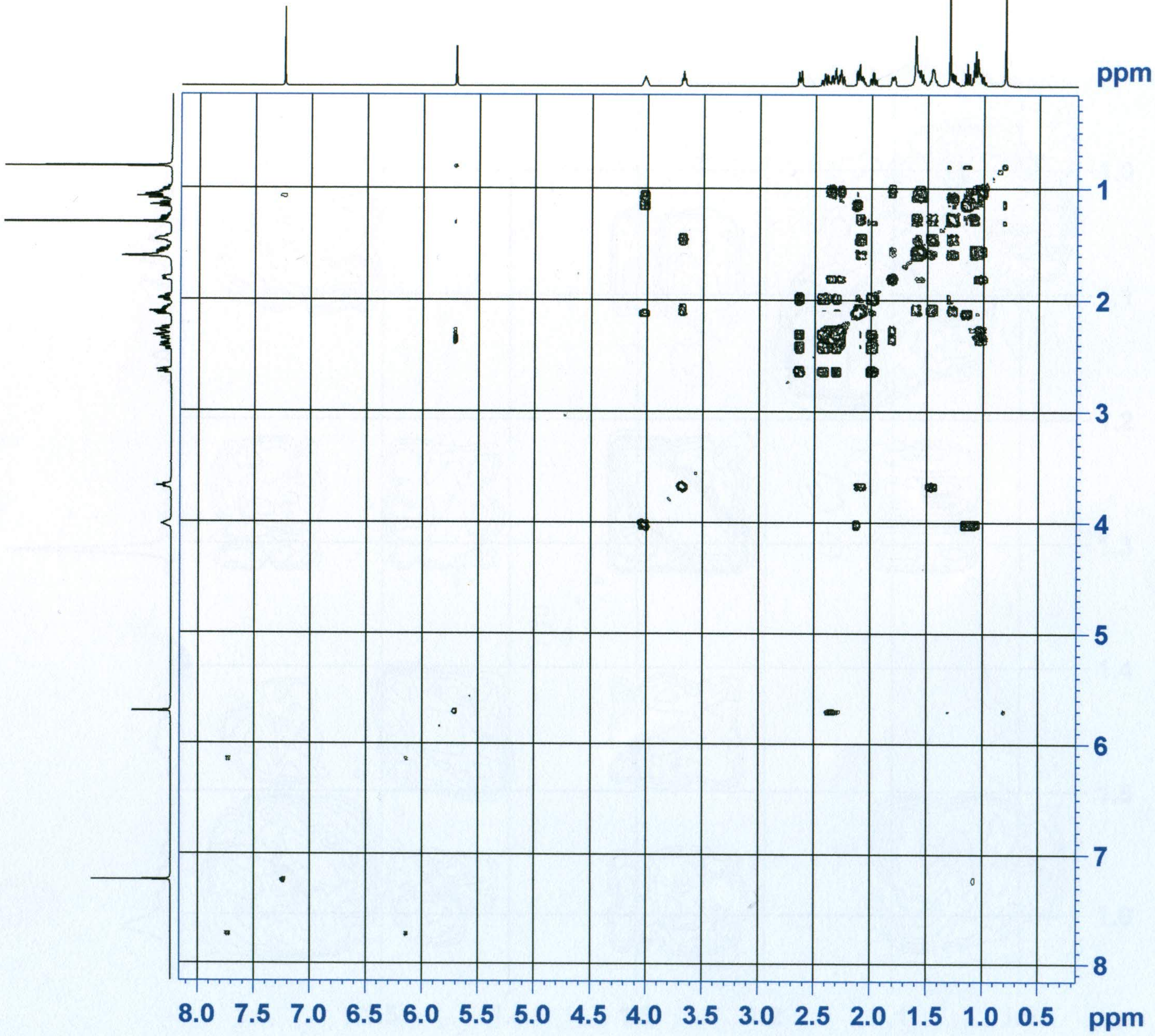

BRUKER

NAME	Oct 24	
EXPNO	2	
PROCNO	1	
Date_	20091024	
Time	14.43	
INSTRUM	spect	
PROBHD	5 mm CPTCI 1H-	
PULPROG	cosydfqf	
TD	2048	
SOLVENT	CDCl3	
NS	8	
DS	4	
SWH	4807.692	Hz
FIDRES	2.347506	Hz
AQ	0.2131460	sec
RG	35.9	
DW	104.000	usec
DE	6.50	usec
TE	294.4	K
D0	0.00000300	sec
D1	1.50000000	sec
D13	0.00000400	sec
D20	0.00000200	sec
IN0	0.00020800	sec
======== CHANNEL f1 ========		
NUC1	1H	
P1	7.40	usec
PL1	3.30	dB
PL1W	9.16420078	W
SFO1	600.2325210	MHz
ND0	1	
TD	256	
SFO1	600.2325	MHz
FIDRES	18.780046	Hz
SW	8.010	ppm
FnMODE	QF	
SI	1024	
SF	600.2300250	MHz
WDW	QSINE	
SSB	0	
LB	0.00	Hz
GB	0	
PC	1.40	
SI	1024	
MC2	QF	
SF	600.2300250	MHz
WDW	QSINE	
SSB	0	
LB	0.00	Hz
GB	0	

AVANCE AV 600
LAB. No. 108

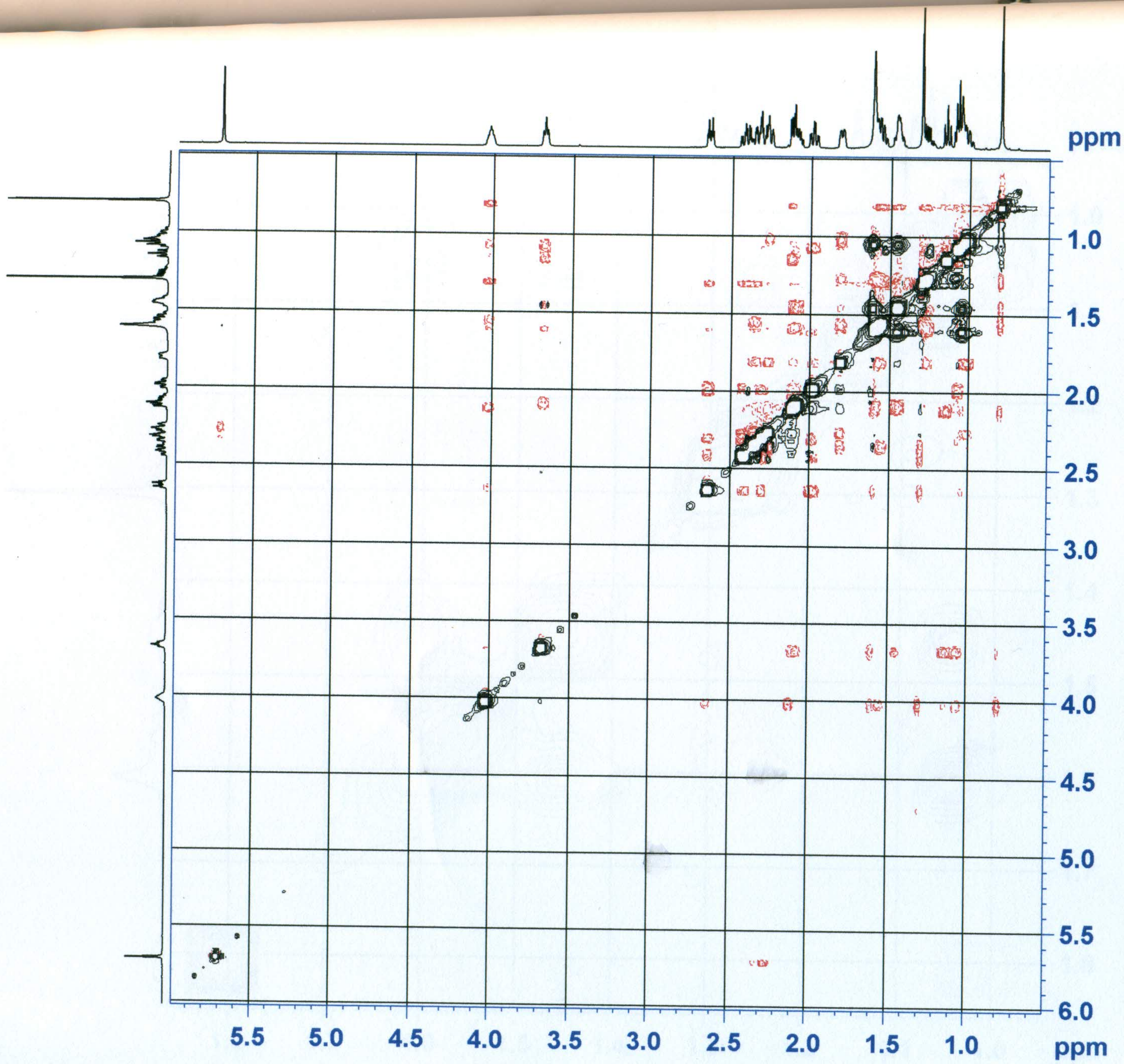

BRUKER

NAME	Oct 24	
EXPNO	3	
PROCNO	1	
Date_	20091024	
Time	15.43	
INSTRUM	spect	
PROBHD	5 mm CPTCI 1H-	
PULPROG	noesygpph	
TD	1024	
SOLVENT	CDCl3	
NS	16	
DS	4	
SWH	4807.692	Hz
FIDRES	4.695012	Hz
AQ	0.1066500	sec
RG	57	
DW	104.000	usec
DE	6.50	usec
TE	294.5	K
D0	0.00009458	sec
D1	1.50000000	sec
D8	0.80000001	sec
D16	0.00015000	sec
IN0	0.00020800	sec

======== CHANNEL f1 ========

NUC1	1H	
P1	7.40	usec
P2	14.80	usec
PL1	3.30	dB
PL1W	9.16420078	W
SFO1	600.2325210	MHz

====== GRADIENT CHANNEL =====

GPNAM1	SINE.100	
GPNAM2	SINE.100	
GPZ1	40.00	%
GPZ2	-40.00	%
P16	2000.00	usec
ND0	1	
TD	256	
SFO1	600.2325	MHz
FIDRES	18.780046	Hz
SW	8.010	ppm
FnMODE	States-TPPI	
SI	1024	
SF	600.2300250	MHz
WDW	QSINE	
SSB	2	
LB	0.00	Hz
GB	0	
PC	1.40	
SI	1024	
MC2	States-TPPI	
SF	600.2300250	MHz
WDW	QSINE	
SSB	2	
LB	0.00	Hz
GB	0	

AVANCE AV 600
LAB. No. 108
