# Supplementary material for: Molecular docking simulation studies on potent butyrylcholinesterase inhibitors obtained from microbial transformation of dihydrotestosterone
Source: Chem Cent J. 2013 Oct 8;7:164. doi: 10.1186/1752-153X-7-164 (PMC4126177; doi:10.1186/1752-153X-7-164)
Supplement: Additional file 4 — 1H-, 13C- and 2D-NMR spectra of compound 5. [file 1752-153X-7-164-S4.pdf]

Salman/Dr,Iqbal/TGF-F6-1/

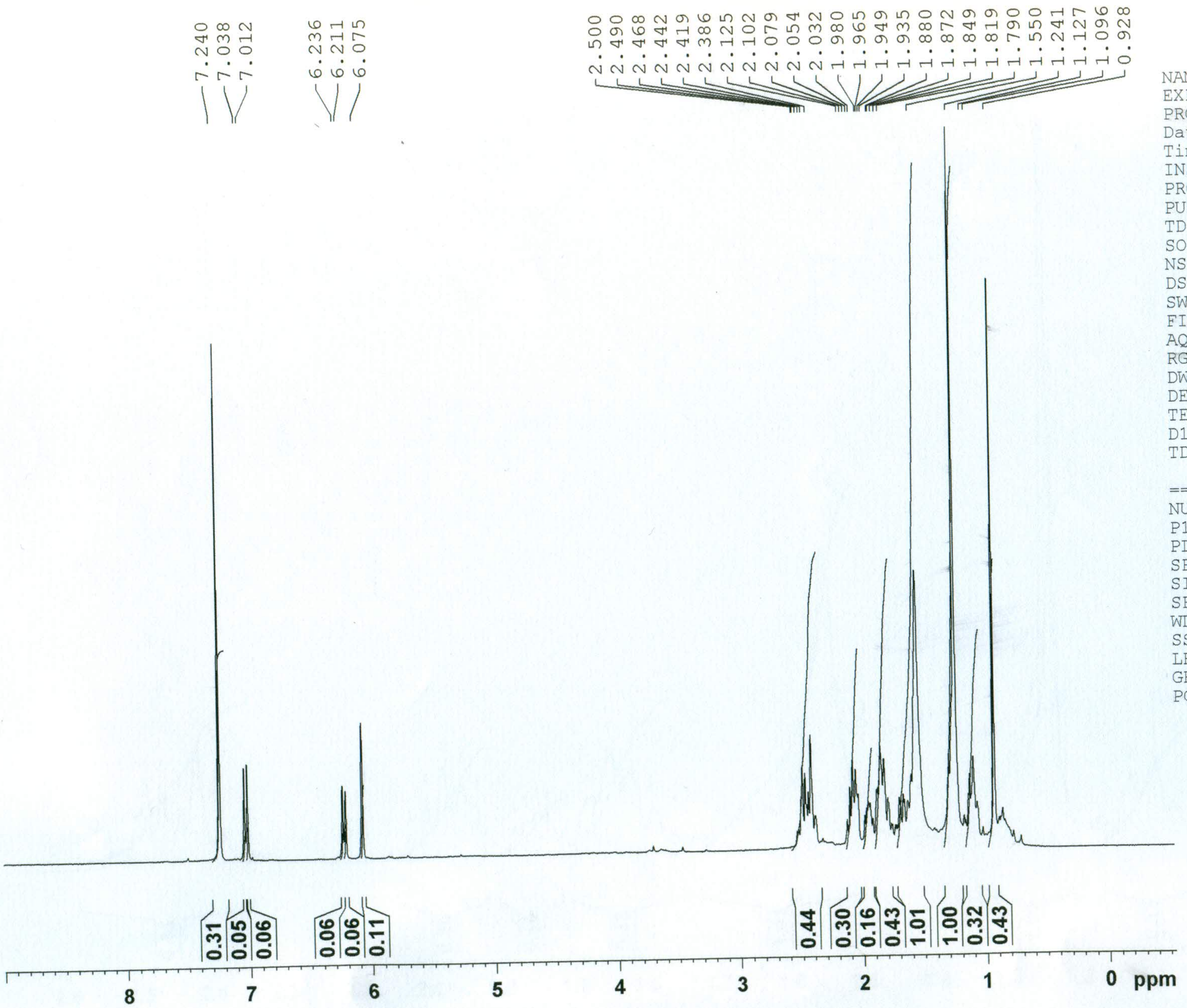


| | |
|---|---|
| NAME | Oct08 |
| EXPNO | 2 |
| PROCNO | 1 |
| Date_ | 20091008 |
| Time_ | 10.44 |
| INSTRUM | spect |
| PROBHD | 5 mm Dual 13C/ |
| PULPROG | zg30 |
| TD | 32768 |
| SOLVENT | CDCl3 |
| NS | 128 |
| DS | 0 |
| SWH | 8012.820 Hz |
| FIDRES | 0.244532 Hz |
| AQ | 2.0447731 sec |
| RG | 512 |
| DW | 62.400 usec |
| DE | 6.50 usec |
| TE | 300.7 K |
| D1 | 1.00000000 sec |
| TD0 | 1 |

======== CHANNEL f1 ========

| | |
|---|---|
| NUC1 | 1H |
| P1 | 8.10 usec |
| PL1 | 4.00 dB |
| SFO1 | 400.2328016 MHz |
| SI | 16384 |
| SF | 400.2300132 MHz |
| WDW | EM |
| SSB | 0 |
| LB | 0.30 Hz |
| GB | 0 |
| PC | 2.00 |

AVANCE 400-A
LAB. No. 109

Sample: TGFF61

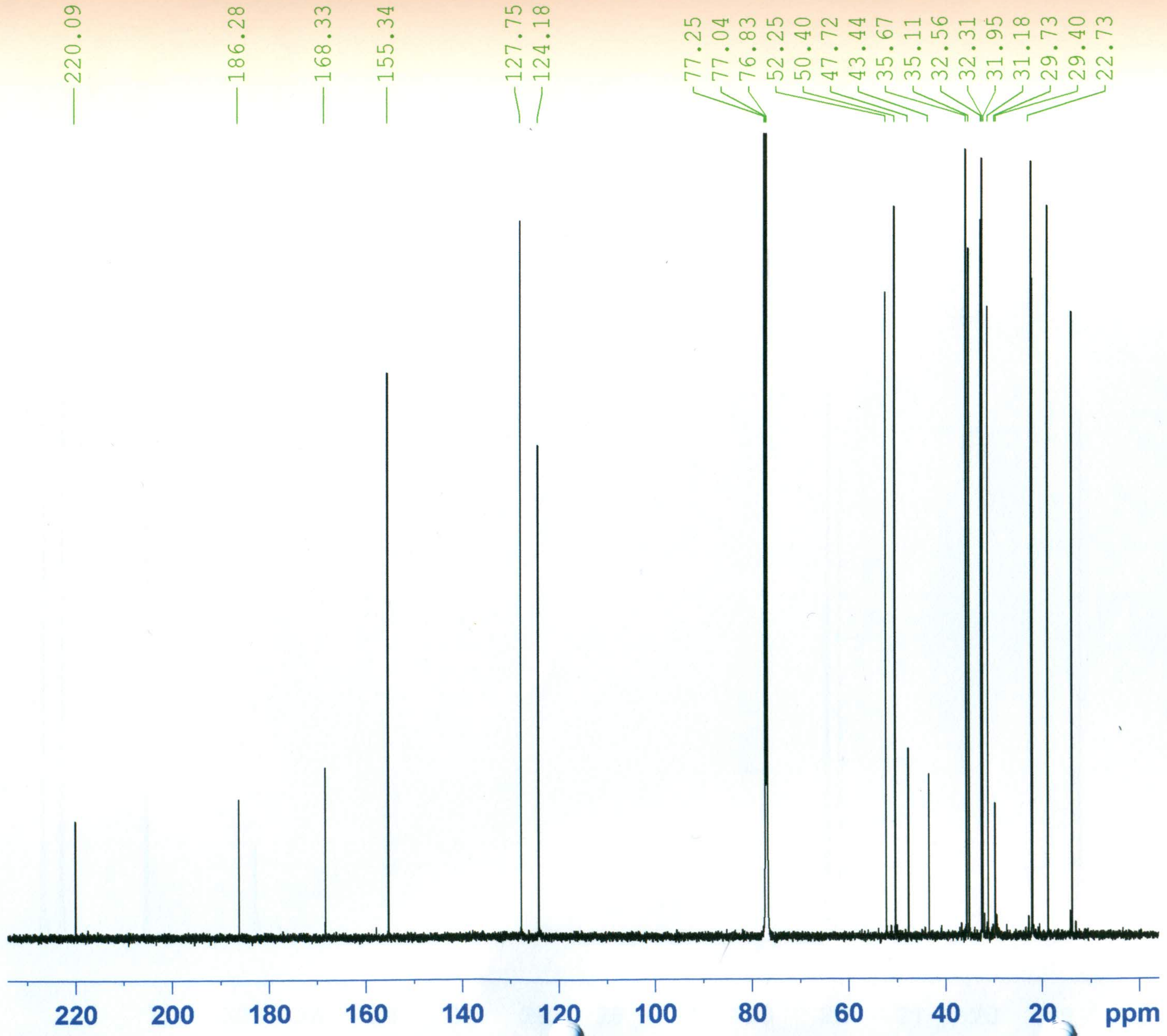


BRUKER

AVANCE AV 600
LAB. No. 108

| | | |
|---|---|---|
| NAME | Oct 26 | |
| EXPNO | 6 | |
| PROCNO | 1 | |
| Date_ | 20091026 | |
| Time | 22.00 | |
| INSTRUM | spect | |
| PROBHD | 5 mm CPTCI 1H- | |
| PULPROG | zgpg | |
| TD | 65536 | |
| SOLVENT | CDCl3 | |
| NS | 12288 | |
| DS | 2 | |
| SWH | 35971.223 | Hz |
| FIDRES | 0.548877 | Hz |
| AQ | 0.9110143 | sec |
| RG | 32768 | |
| DW | 13.900 | usec |
| DE | 6.50 | usec |
| TE | 294.1 | K |
| D1 | 1.50000000 | sec |
| D11 | 0.03000000 | sec |
| TD0 | 12 | |
| ======== CHANNEL f1 ======== | | |
| NUC1 | 13C | |
| P1 | 16.00 | usec |
| PL1 | 2.00 | dB |
| PL1W | 66.40702820 | W |
| SFO1 | 150.9453107 | MHz |
| ======== CHANNEL f2 ======== | | |
| CPDPRG2 | waltz16 | |
| NUC2 | 1H | |
| PCPD2 | 65.00 | usec |
| PL2 | 3.30 | dB |
| PL12 | 22.06 | dB |
| PL13 | 27.00 | dB |
| PL2W | 9.16420078 | W |
| PL12W | 0.12192553 | W |
| PL13W | 0.03909260 | W |
| SFO2 | 600.2336014 | MHz |
| SI | 32768 | |
| SF | 150.9279540 | MHz |
| WDW | EM | |
| SSB | 0 | |
| LB | 1.00 | Hz |
| GB | 0 | |
| PC | 1.40 | |

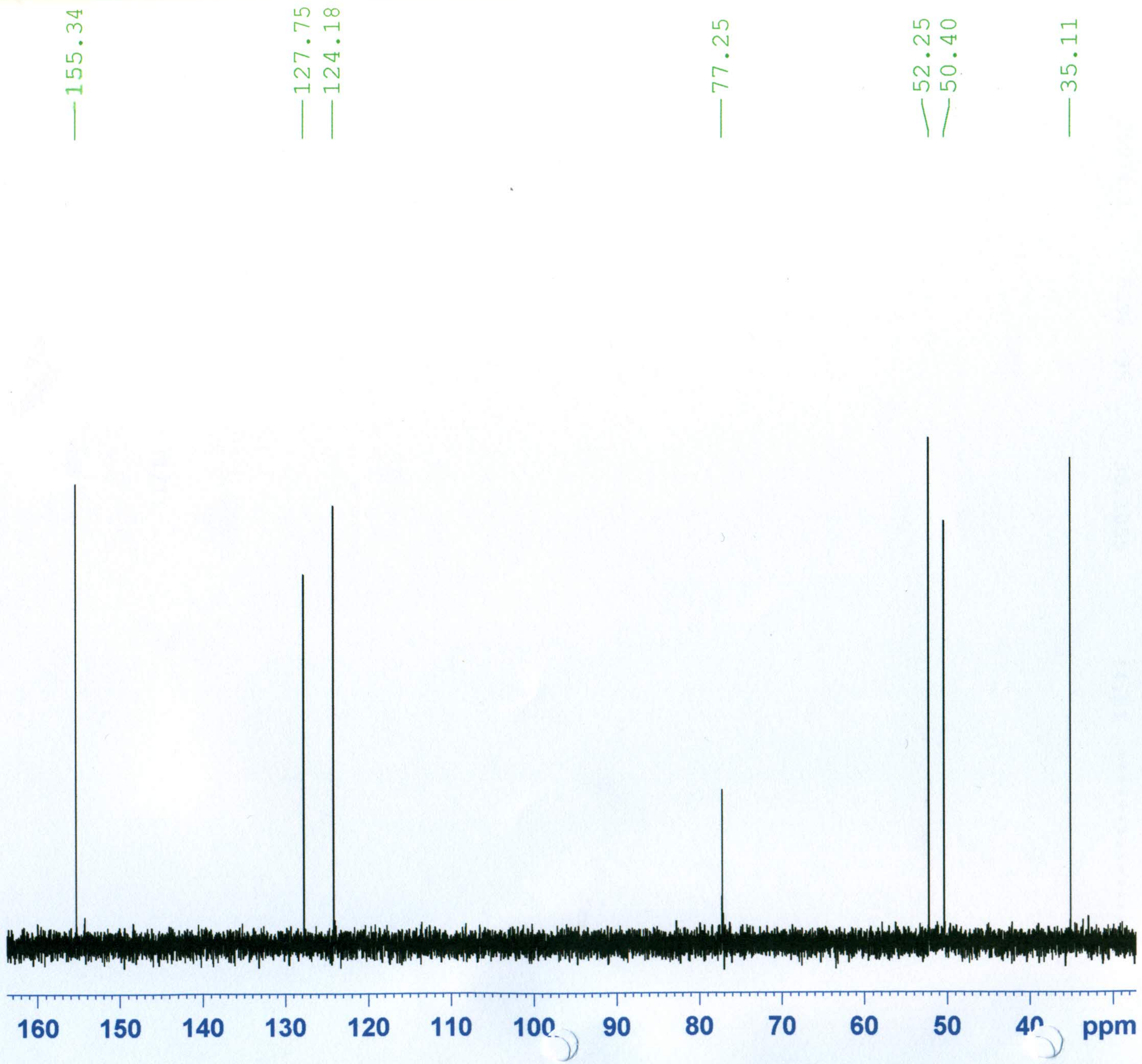


BRUKER

AVANCE AV 600
LAB. NO. 106

| | | |
|---|---|---|
| NAME | Oct 26 | |
| EXPNO | 8 | |
| PROCNO | 1 | |
| Date_ | 20091027 | |
| Time | 8.24 | |
| INSTRUM | spect | |
| PROBHD | 5 mm CPTCI 1H- | |
| PULPROG | deptsp90 | |
| TD | 65536 | |
| SOLVENT | CDCl3 | |
| NS | 334 | |
| DS | 2 | |
| SWH | 30303.031 | Hz |
| FIDRES | 0.462388 | Hz |
| AQ | 1.0814105 | sec |
| RG | 32768 | |
| DW | 16.500 | usec |
| DE | 6.50 | usec |
| TE | 293.6 | K |
| CNST2 | 145.0000000 | |
| D1 | 1.50000000 | sec |
| D2 | 0.00344828 | sec |
| D12 | 0.00002000 | sec |
| TD0 | 6 | |
| ======== CHANNEL f1 ======== | | |
| NUC1 | 13C | |
| P1 | 16.00 | usec |
| P12 | 2000.00 | usec |
| PL0 | 120.00 | dB |
| PL1 | 2.00 | dB |
| PL0W | 0.00000000 | W |
| PL1W | 66.40702820 | W |
| SFO1 | 150.9430463 | MHz |
| SP2 | 1.99 | dB |
| SPNAM2 | Crp60comp.4 | |
| SPOAL2 | 0.500 | |
| SPOFFS2 | 0.00 | Hz |
| ======== CHANNEL f2 ======== | | |
| CPDPRG2 | waltz16 | |
| NUC2 | 1H | |
| P3 | 7.50 | usec |
| P4 | 15.00 | usec |
| PCPD2 | 65.00 | usec |
| PL2 | 3.30 | dB |
| PL12 | 22.06 | dB |
| PL2W | 9.16420078 | W |
| PL12W | 0.12192553 | W |
| SFO2 | 600.2324009 | MHz |
| SI | 32768 | |
| SF | 150.9279540 | MHz |
| WDW | EM | |
| SSB | 0 | |
| LB | 1.00 | Hz |
| GB | 0 | |
| PC | 1.40 | |

Salman/Dr.Iqbal
Sample: TGFF61

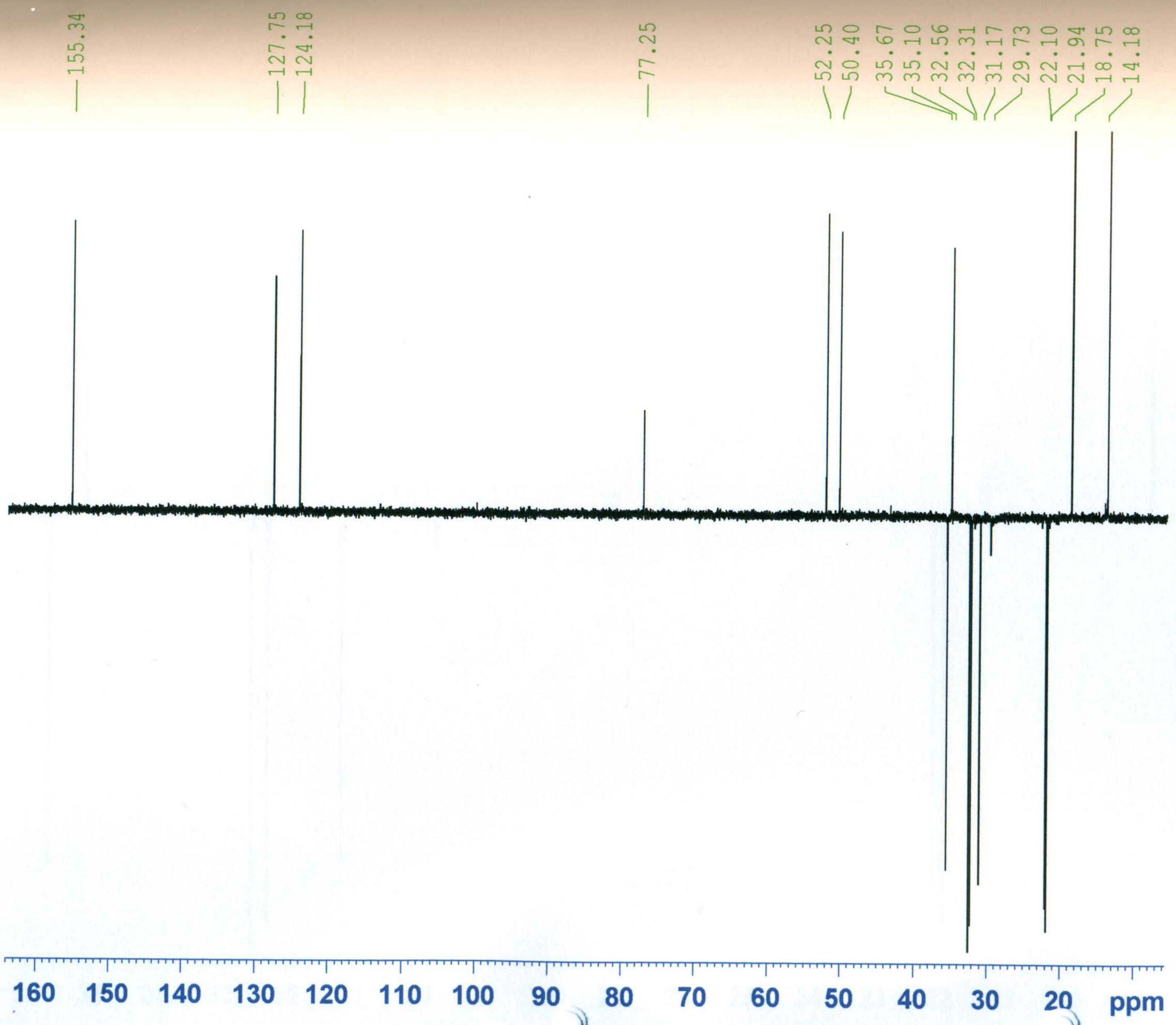


BRUKER

AVANCE AV 600
LAB. NO. 106

| | | |
|---|---|---|
| NAME | Oct 26 | |
| EXPNO | 7 | |
| PROCNO | 1 | |
| Date_ | 20091027 | |
| Time | 6.30 | |
| INSTRUM | spect | |
| PROBHD | 5 mm CPTCI 1H- | |
| PULPROG | deptsp135 | |
| TD | 65536 | |
| SOLVENT | CDCl3 | |
| NS | 3283 | |
| DS | 2 | |
| SWH | 30303.031 | Hz |
| FIDRES | 0.462388 | Hz |
| AQ | 1.0814105 | sec |
| RG | 32768 | |
| DW | 16.500 | usec |
| DE | 6.50 | usec |
| TE | 293.4 | K |
| CNST2 | 145.0000000 | |
| D1 | 1.50000000 | sec |
| D2 | 0.00344828 | sec |
| D12 | 0.00002000 | sec |
| TD0 | 6 | |

======== CHANNEL f1 ========

| | | |
|---|---|---|
| NUC1 | 13C | |
| P1 | 16.00 | usec |
| P12 | 2000.00 | usec |
| PL0 | 120.00 | dB |
| PL1 | 2.00 | dB |
| PL0W | 0.00000000 | W |
| PL1W | 66.40702820 | W |
| SFO1 | 150.9430463 | MHz |
| SP2 | 1.99 | dB |
| SPNAM2 | Crp60comp.4 | |
| SPOAL2 | 0.500 | |
| SPOFFS2 | 0.00 | Hz |

======== CHANNEL f2 ========

| | | |
|---|---|---|
| CPDPRG2 | waltz16 | |
| NUC2 | 1H | |
| P3 | 7.50 | usec |
| P4 | 15.00 | usec |
| PCPD2 | 65.00 | usec |
| PL2 | 3.30 | dB |
| PL12 | 22.06 | dB |
| PL2W | 9.16420078 | W |
| PL12W | 0.12192553 | W |
| SFO2 | 600.2324009 | MHz |
| SI | 32768 | |
| SF | 150.9279540 | MHz |
| WDW | EM | |
| SSB | 0 | |
| LB | 1.00 | Hz |
| GB | 0 | |
| PC | 1.40 | |

Sample; TGFF61

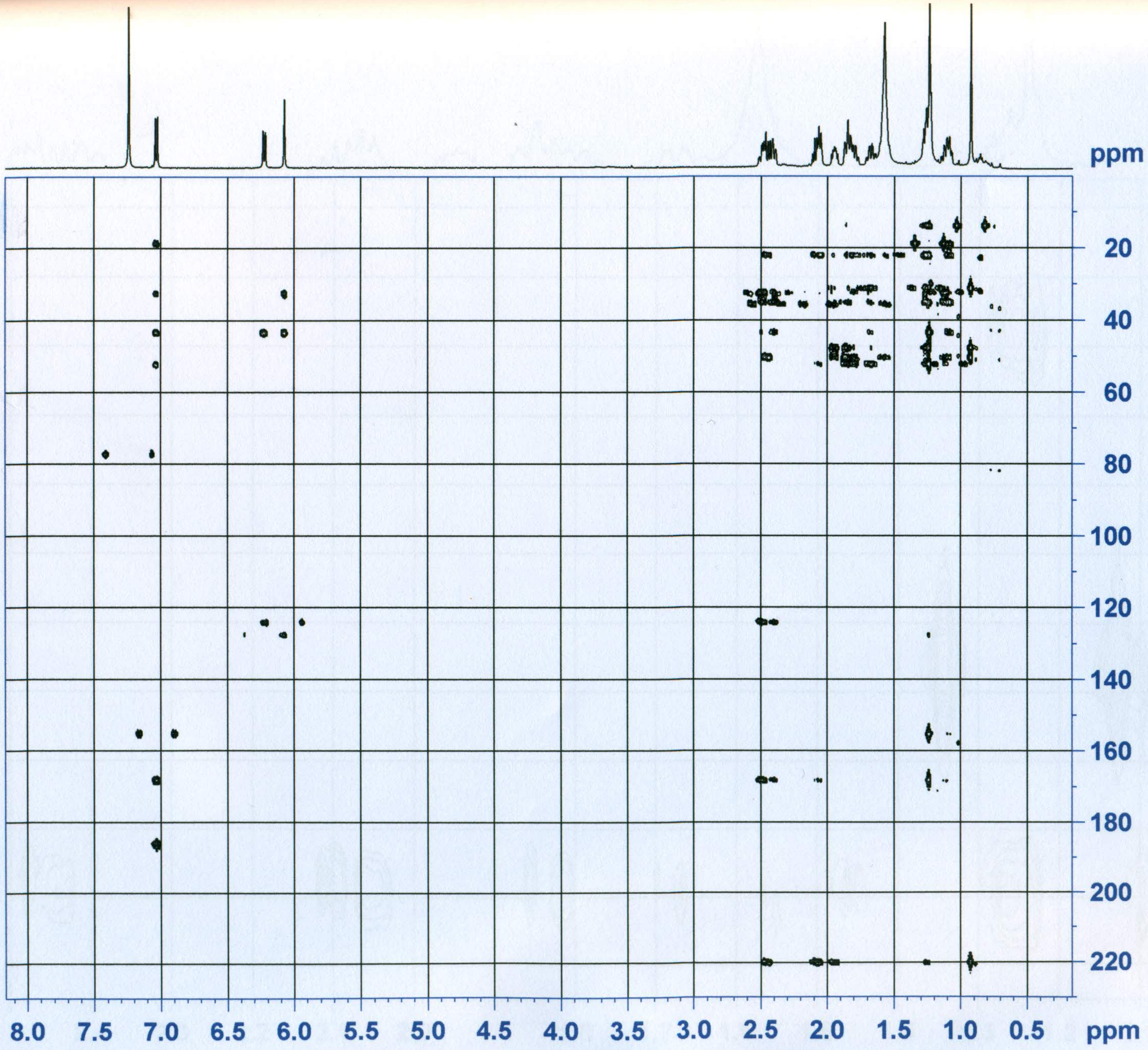


BRUKER

```
NAME             Oct 26
EXPNO                 5
PROCNO                1
Date_          20091026
Time              16.48
INSTRUM           spect
PROBHD   5 mm CPTCI 1H-
PULPROG    hmbcgplpndqf
TD                 4096
SOLVENT           CDCl3
NS                   32
DS                    8
SWH            4807.692 Hz
FIDRES         1.173753 Hz
AQ            0.4261380 sec
RG                46341
DW              104.000 usec
DE                 6.50 usec
TE                294.5 K
CNST2       145.0000000
CNST13       13.0000000
D0           0.00000300 sec
D1           1.50000000 sec
D2           0.00344828 sec
D6           0.03846154 sec
D16          0.00015000 sec
IN0          0.00001440 sec

======== CHANNEL f1 ========
NUC1                 1H
P1                 7.40 usec
P2                14.80 usec
PL1                3.30 dB
PL1W         9.16420078 W
SFO1        600.2325210 MHz

======== CHANNEL f2 ========
NUC2                13C
P3                10.00 usec
PL2                2.00 dB
PL2W        66.40702820 W
SFO2        150.9453107 MHz

====== GRADIENT CHANNEL =====
GPNAM1         SINE.100
GPNAM2         SINE.100
GPNAM3         SINE.100
GPZ1              50.00 %
GPZ2              30.00 %
GPZ3              40.10 %
P16             2000.00 usec
ND0                   2
TD                  256
SFO1           150.9453 MHz
FIDRES       135.614929 Hz
SW              230.000 ppm
FnMODE               QF
SI                 1024
SF          600.2300250 MHz
WDW                SINE
SSB                   0
LB                 0.00 Hz
GB                    0
PC                 1.40
SI                 1024
MC2                  QF
SF          150.9279540 MHz
WDW                SINE
SSB                   0
LB                 0.00 Hz
GB                    0
```

AVANCE AV 600
LAB. No. 108

[illegible]
Sample: TGPF61

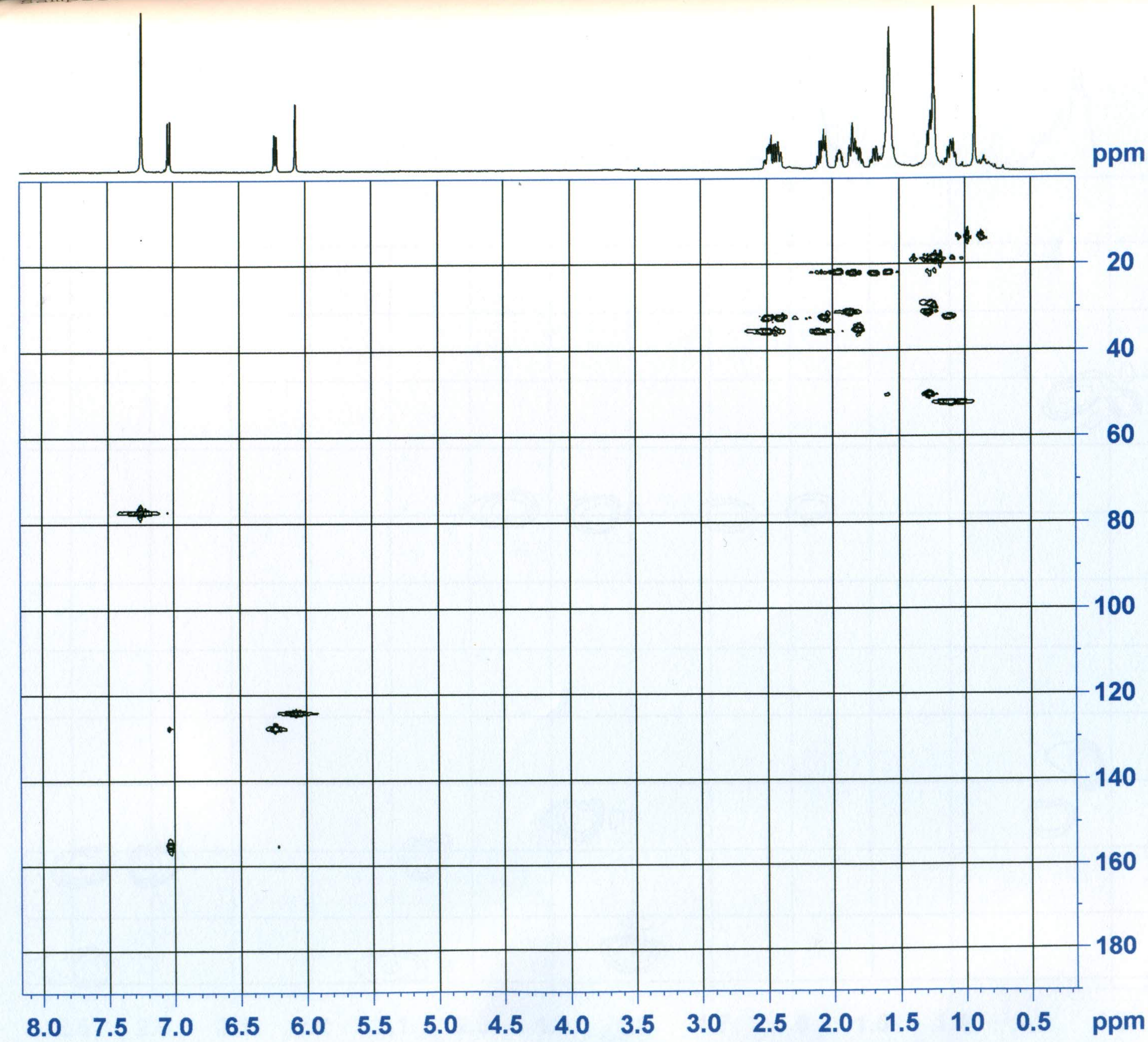


BRUKER

AVANCE AV 600
LAB. No. 108

| | |
|---|---|
| NAME | Oct 26 |
| EXPNO | 4 |
| PROCNO | 1 |
| Date_ | 20091026 |
| Time | 13.05 |
| INSTRUM | spect |
| PROBHD | 5 mm CPTCI 1H- |
| PULPROG | hsqcetgpsi |
| TD | 1024 |
| SOLVENT | CDCl3 |
| NS | 32 |
| DS | 8 |
| SWH | 4807.692 Hz |
| FIDRES | 4.695012 Hz |
| AQ | 0.1066500 sec |
| RG | 46341 |
| DW | 104.000 usec |
| DE | 6.50 usec |
| TE | 294.5 K |
| CNST2 | 145.0000000 |
| D0 | 0.00000300 sec |
| D1 | 1.50000000 sec |
| D4 | 0.00172414 sec |
| D11 | 0.03000000 sec |
| D13 | 0.00000400 sec |
| D16 | 0.00015000 sec |
| D24 | 0.00110000 sec |
| IN0 | 0.00001745 sec |
| ZGOPTNS | |

======== CHANNEL f1 ========

| | |
|---|---|
| NUC1 | 1H |
| P1 | 7.40 usec |
| P2 | 14.80 usec |
| P28 | 0.50 usec |
| PL1 | 3.30 dB |
| PL1W | 9.16420078 W |
| SFO1 | 600.2325210 MHz |

======== CHANNEL f2 ========

| | |
|---|---|
| CPDPRG2 | garp |
| NUC2 | 13C |
| P3 | 10.00 usec |
| P4 | 20.00 usec |
| PCPD2 | 60.00 usec |
| PL2 | 2.00 dB |
| PL12 | 17.56 dB |
| PL2W | 66.40702820 W |
| PL12W | 1.84592509 W |
| SFO2 | 150.9422922 MHz |

====== GRADIENT CHANNEL =====

| | |
|---|---|
| GPNAM1 | SINE.100 |
| GPNAM2 | SINE.100 |
| GPZ1 | 80.00 % |
| GPZ2 | 20.10 % |
| P16 | 2000.00 usec |
| ND0 | 2 |
| TD | 256 |
| SFO1 | 150.9423 MHz |
| FIDRES | 112.027481 Hz |
| SW | 190.000 ppm |
| FnMODE | Echo-Antiecho |
| SI | 1024 |
| SF | 600.2300250 MHz |
| WDW | QSINE |
| SSB | 2 |
| LB | 0.00 Hz |
| GB | 0 |
| PC | 4.00 |
| SI | 1024 |
| MC2 | echo-antiecho |
| SF | 150.9279540 MHz |
| WDW | QSINE |
| SSB | 2 |
| LB | 0.00 Hz |
| GB | 0 |

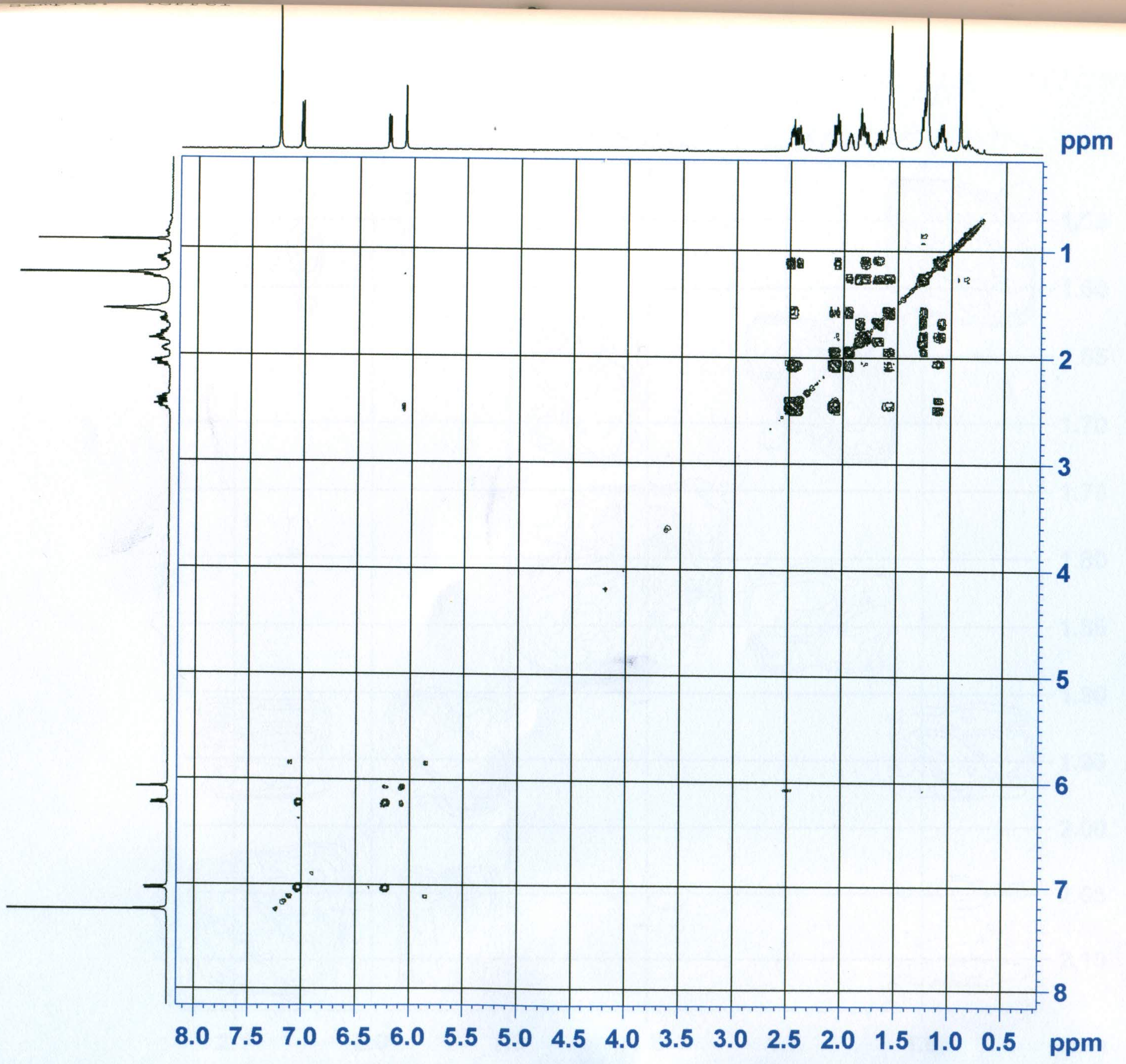


BRUKER

| | | |
|---|---|---|
| NAME | Oct 26 | |
| EXPNO | 2 | |
| PROCNO | 1 | |
| Date_ | 20091026 | |
| Time | 9.18 | |
| INSTRUM | spect | |
| PROBHD | 5 mm CPTCI 1H- | |
| PULPROG | cosydfqf | |
| TD | 2048 | |
| SOLVENT | CDCl3 | |
| NS | 8 | |
| DS | 4 | |
| SWH | 4807.692 | Hz |
| FIDRES | 2.347506 | Hz |
| AQ | 0.2131460 | sec |
| RG | 35.9 | |
| DW | 104.000 | usec |
| DE | 6.50 | usec |
| TE | 294.3 | K |
| D0 | 0.00000300 | sec |
| D1 | 1.50000000 | sec |
| D13 | 0.00000400 | sec |
| D20 | 0.00000200 | sec |
| IN0 | 0.00020800 | sec |

======== CHANNEL f1 ========

| | | |
|---|---|---|
| NUC1 | 1H | |
| P1 | 7.40 | usec |
| PL1 | 3.30 | dB |
| PL1W | 9.16420078 | W |
| SFO1 | 600.2325210 | MHz |
| ND0 | 1 | |
| TD | 256 | |
| SFO1 | 600.2325 | MHz |
| FIDRES | 18.780046 | Hz |
| SW | 8.010 | ppm |
| FnMODE | QF | |
| SI | 1024 | |
| SF | 600.2300250 | MHz |
| WDW | QSINE | |
| SSB | 0 | |
| LB | 0.00 | Hz |
| GB | 0 | |
| PC | 1.40 | |
| SI | 1024 | |
| MC2 | QF | |
| SF | 600.2300250 | MHz |
| WDW | QSINE | |
| SSB | 0 | |
| LB | 0.00 | Hz |
| GB | 0 | |

[illegible]ANCE AV 600
LAB. No. 108

Sample: TGFF61

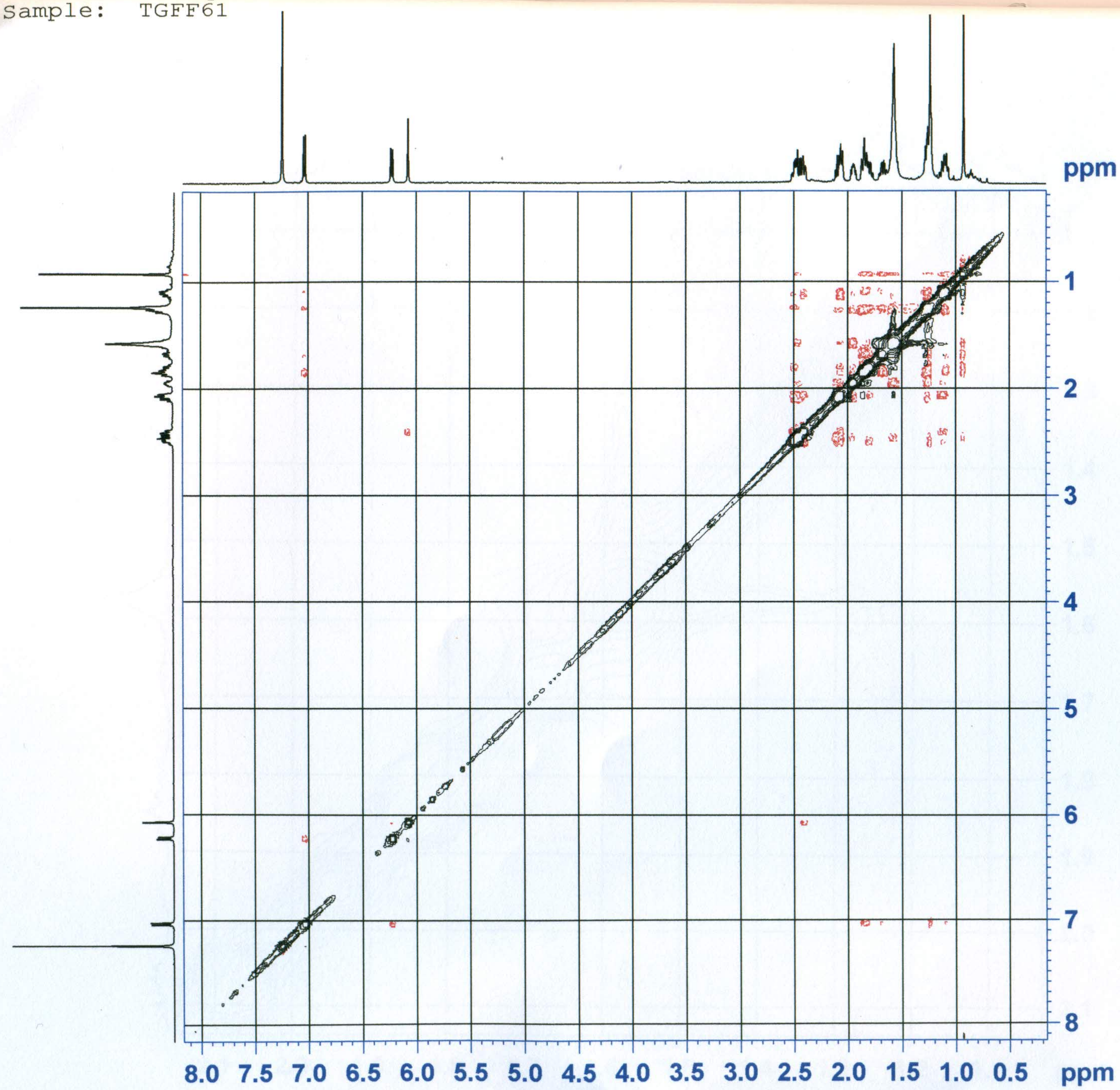


BRUKER

| | | |
|---|---|---|
| NAME | Oct 26 | |
| EXPNO | 3 | |
| PROCNO | 1 | |
| Date_ | 20091026 | |
| Time_ | 10.18 | |
| INSTRUM | spect | |
| PROBHD | 5 mm CPTCI 1H- | |
| PULPROG | noesygpph | |
| TD | 1024 | |
| SOLVENT | CDCl3 | |
| NS | 16 | |
| DS | 4 | |
| SWH | 4807.692 | Hz |
| FIDRES | 4.695012 | Hz |
| AQ | 0.1066500 | sec |
| RG | 57 | |
| DW | 104.000 | usec |
| DE | 6.50 | usec |
| TE | 294.6 | K |
| D0 | 0.00009458 | sec |
| D1 | 1.50000000 | sec |
| D8 | 0.80000001 | sec |
| D16 | 0.00015000 | sec |
| IN0 | 0.00020800 | sec |
| ======== CHANNEL f1 ======== | | |
| NUC1 | 1H | |
| P1 | 7.40 | usec |
| P2 | 14.80 | usec |
| PL1 | 3.30 | dB |
| PL1W | 9.16420078 | W |
| SFO1 | 600.2325210 | MHz |
| ====== GRADIENT CHANNEL ===== | | |
| GPNAM1 | SINE.100 | |
| GPNAM2 | SINE.100 | |
| GPZ1 | 40.00 | % |
| GPZ2 | -40.00 | % |
| P16 | 2000.00 | usec |
| ND0 | 1 | |
| TD | 256 | |
| SFO1 | 600.2325 | MHz |
| FIDRES | 18.780046 | Hz |
| SW | 8.010 | ppm |
| FnMODE | States-TPPI | |
| SI | 1024 | |
| SF | 600.2300250 | MHz |
| WDW | QSINE | |
| SSB | 2 | |
| LB | 0.00 | Hz |
| GB | 0 | |
| PC | 1.40 | |
| SI | 1024 | |
| MC2 | States-TPPI | |
| SF | 600.2300250 | MHz |
| WDW | QSINE | |
| SSB | 2 | |
| LB | 0.00 | Hz |
| GB | 0 | |

AVANCE AV 600
LAB. No. 108
