# Supplementary material for: Molecular docking simulation studies on potent butyrylcholinesterase inhibitors obtained from microbial transformation of dihydrotestosterone
Source: Chem Cent J. 2013 Oct 8;7:164. doi: 10.1186/1752-153X-7-164 (PMC4126177; doi:10.1186/1752-153X-7-164)
Supplement: Additional file 5 — 1H-, 13C- and 2D-NMR spectra of compound 6. [file 1752-153X-7-164-S5.pdf]

Salman/Dr,Iqbal/TGF-7-31/

AVANCE 400-A
LAB. No. 109

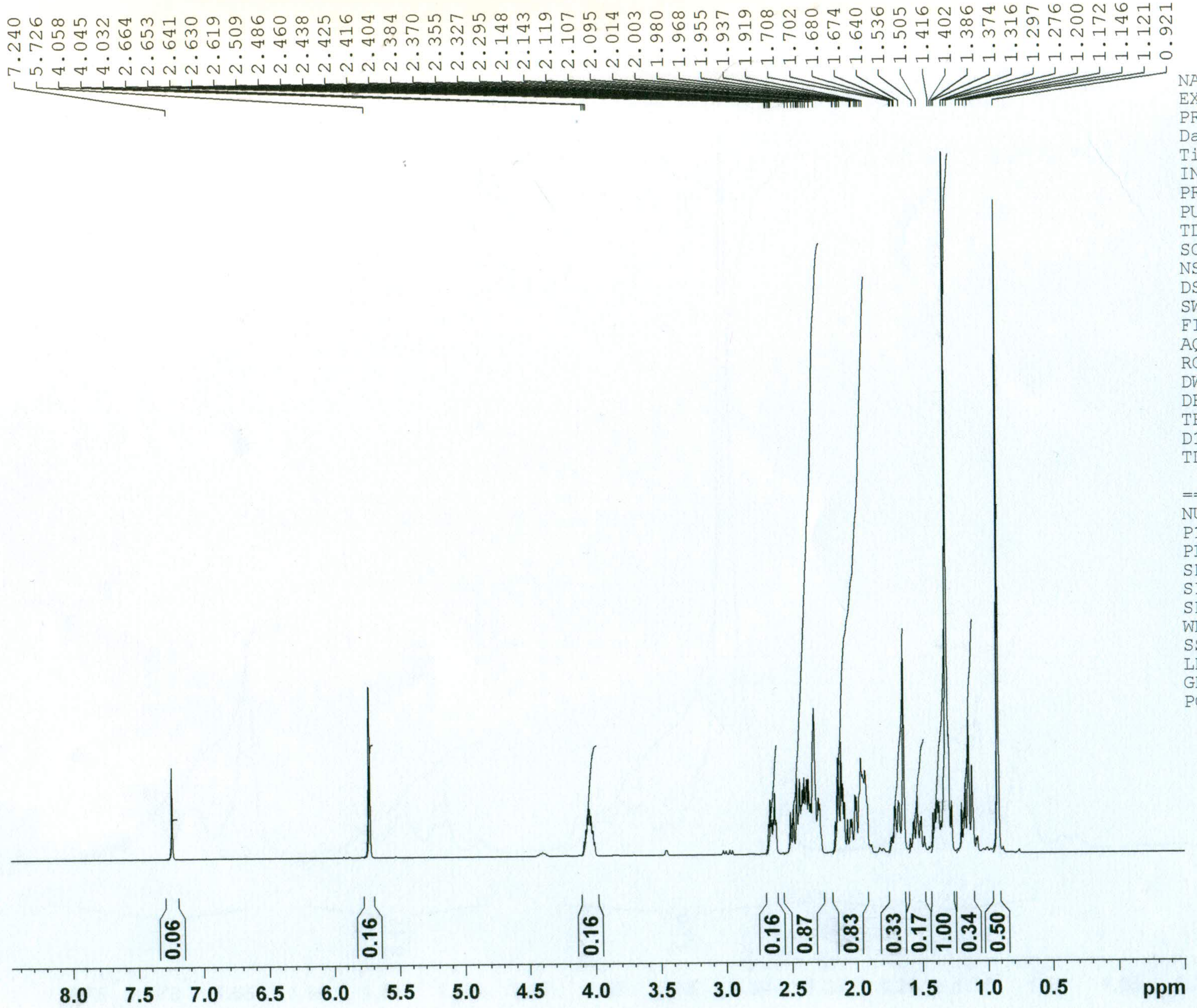

NAME	Oct08	
EXPNO	3	
PROCNO	1	
Date_	20091008	
Time	10.52	
INSTRUM	spect	
PROBHD	5 mm Dual 13C/	
PULPROG	zg30	
TD	32768	
SOLVENT	CDCl3	
NS	64	
DS	0	
SWH	8012.820	Hz
FIDRES	0.244532	Hz
AQ	2.0447731	sec
RG	114	
DW	62.400	usec
DE	6.50	usec
TE	300.6	K
D1	1.00000000	sec
TD0	1	

======== CHANNEL f1 ========

NUC1	1H	
P1	8.10	usec
PL1	4.00	dB
SFO1	400.2328016	MHz
SI	16384	
SF	400.2300132	MHz
WDW	EM	
SSB	0	
LB	0.30	Hz
GB	0	
PC	2.00	

SALMAN ZAFAR/DR.IQBAL/TGF7-3-1/

LAB No. 109-B

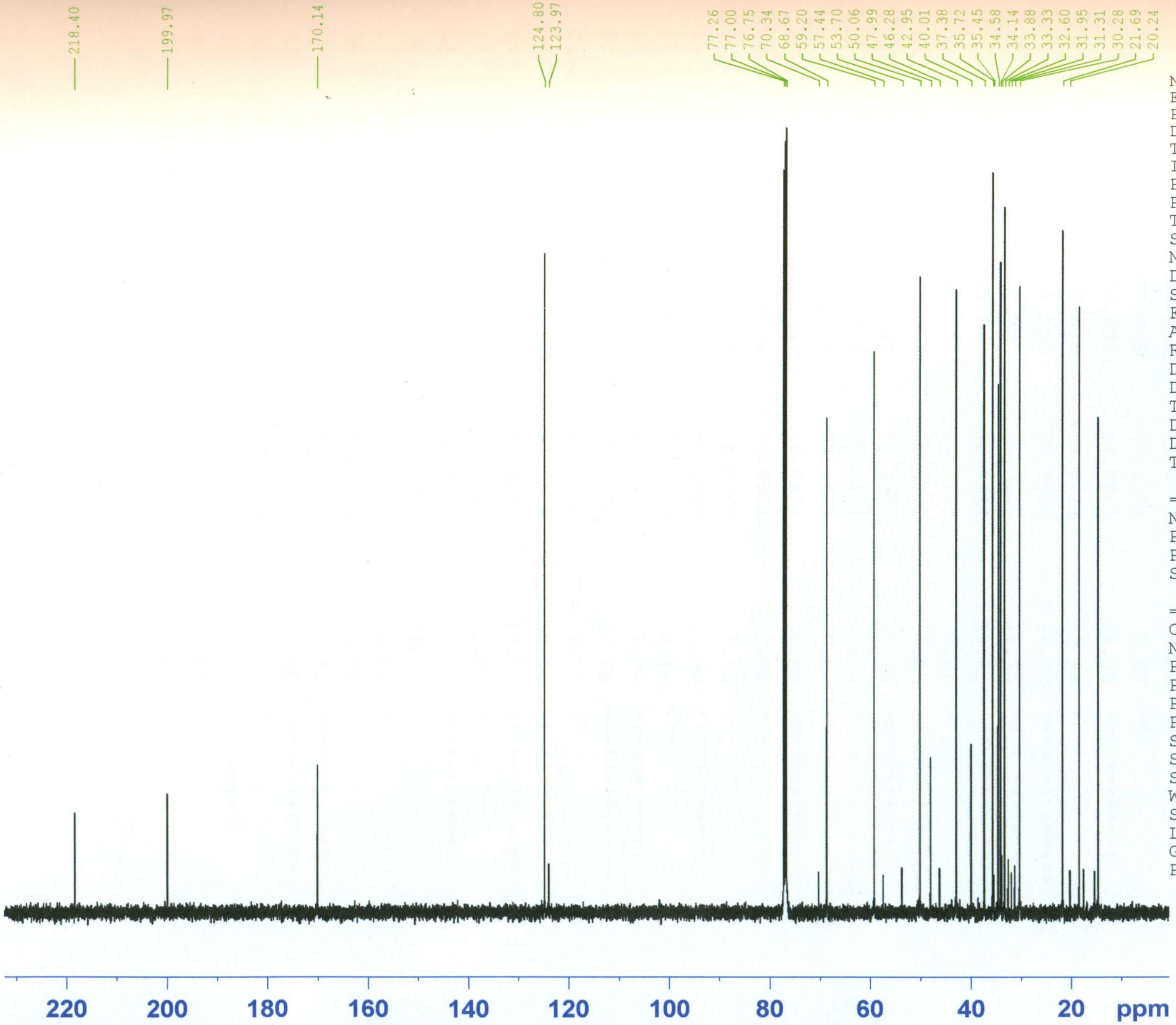

NAME	oct27	
EXPNO	13	
PROCNO	1	
Date_	20091028	
Time	5.42	
INSTRUM	spect	
PROBHD	5 mm BBI 1H-BB	
PULPROG	zgpg	
TD	65536	
SOLVENT	CDCl3	
NS	3860	
DS	0	
SWH	29498.525	Hz
FIDRES	0.450112	Hz
AQ	1.1109022	sec
RG	32768	
DW	16.950	usec
DE	6.50	usec
TE	303.5	K
D1	1.50000000	sec
D11	0.03000000	sec
TD0	10	
======== CHANNEL f1 ========		
NUC1	13C	
P1	11.10	usec
PL1	-3.00	dB
SFO1	125.7723529	MHz
======== CHANNEL f2 ========		
CPDPRG2	waltz16	
NUC2	1H	
PCPD2	100.00	usec
PL2	6.00	dB
PL12	29.48	dB
PL13	23.00	dB
SFO2	500.1325007	MHz
SI	32768	
SF	125.7577908	MHz
WDW	EM	
SSB	0	
LB	1.00	Hz
GB	0	
PC	1.40	

SALMAN ZAFAR/DR.IQBAL/TGF7-3-1/

AVANCE 500
LAB No. 109-B

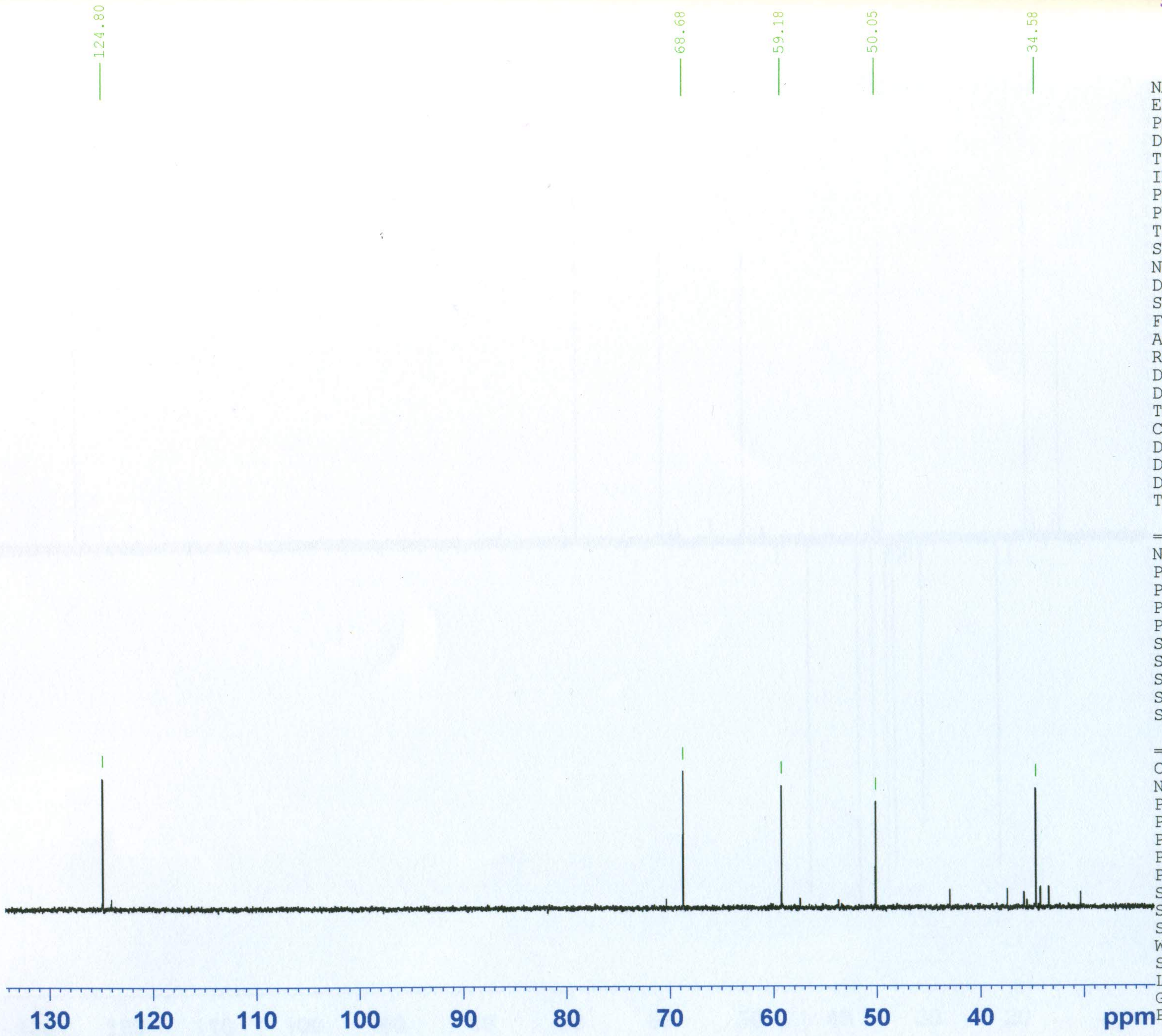

NAME	oct27	
EXPNO	15	
PROCNO	1	
Date_	20091028	
Time	9.33	
INSTRUM	spect	
PROBHD	5 mm BBI 1H-BB	
PULPROG	deptsp90	
TD	32768	
SOLVENT	CDCl3	
NS	889	
DS	2	
SWH	23809.523	Hz
FIDRES	0.726609	Hz
AQ	0.6881990	sec
RG	32768	
DW	21.000	usec
DE	6.50	usec
TE	304.3	K
CNST2	145.0000000	
D1	1.50000000	sec
D2	0.00344828	sec
D12	0.00002000	sec
TD0	5	
======== CHANNEL f1 ========		
NUC1	13C	
P1	11.10	usec
P12	2000.00	usec
PL0	120.00	dB
PL1	-3.00	dB
SFO1	125.7697360	MHz
SP2	1.99	dB
SPNAM2	Crp60comp.4	
SPOAL2	0.500	
SPOFFS2	0.00	Hz
======== CHANNEL f2 ========		
CPDPRG2	waltz16	
NUC2	1H	
P3	7.20	usec
P4	14.40	usec
PCPD2	100.00	usec
PL2	6.00	dB
PL12	29.50	dB
SFO2	500.1335009	MHz
SI	32768	
SF	125.7577908	MHz
WDW	EM	
SSB	0	
LB	1.00	Hz
GB	0	
PC	1.40	

SALMAN ZAFAR/DR.IQBAL/TGF7-3-1/

AVANCE 500
Lab No. 109-B

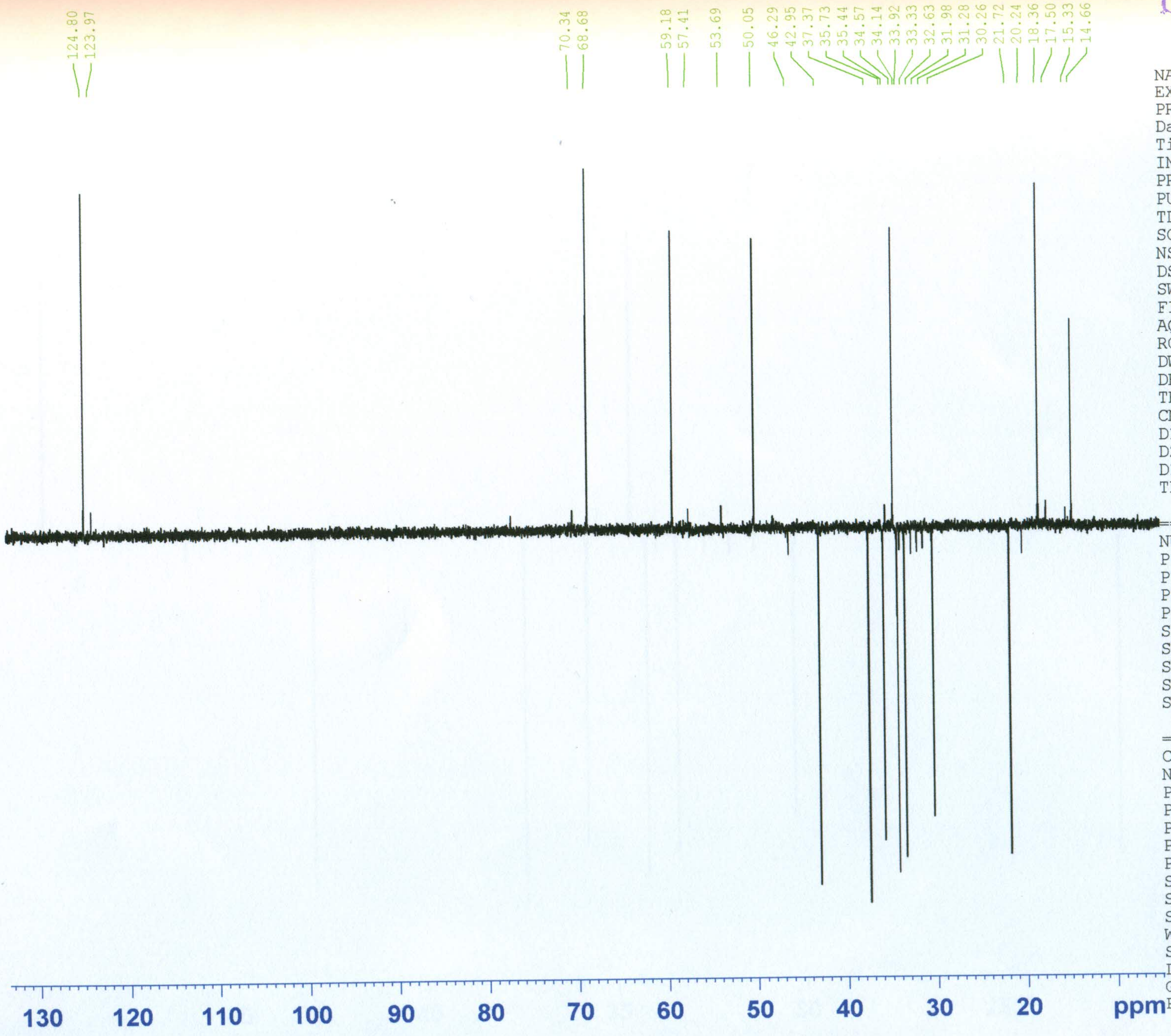

NAME	oct27
EXPNO	14
PROCNO	1
Date_	20091028
Time	8.27
INSTRUM	spect
PROBHD	5 mm BBI 1H-BB
PULPROG	deptsp135
TD	32768
SOLVENT	CDCl3
NS	2236
DS	2
SWH	23809.523 Hz
FIDRES	0.726609 Hz
AQ	0.6881990 sec
RG	32768
DW	21.000 usec
DE	6.50 usec
TE	303.9 K
CNST2	145.0000000
D1	1.50000000 sec
D2	0.00344828 sec
D12	0.00002000 sec
TD0	8

======== CHANNEL f1 ========

NUC1	13C
P1	11.10 usec
P12	2000.00 usec
PL0	120.00 dB
PL1	-3.00 dB
SFO1	125.7697360 MHz
SP2	1.99 dB
SPNAM2	Crp60comp.4
SPOAL2	0.500
SPOFFS2	0.00 Hz

======== CHANNEL f2 ========

CPDPRG2	waltz16
NUC2	1H
P3	7.20 usec
P4	14.40 usec
PCPD2	100.00 usec
PL2	6.00 dB
PL12	29.50 dB
SFO2	500.1335009 MHz
SI	32768
SF	125.7577908 MHz
WDW	EM
SSB	0
LB	1.00 Hz
GB	0
PC	1.40

SALMAN ZAFAR/DR.IQBAL/TGF7-3-1/

AVA[illegible] 500
LA[illegible] 109-B

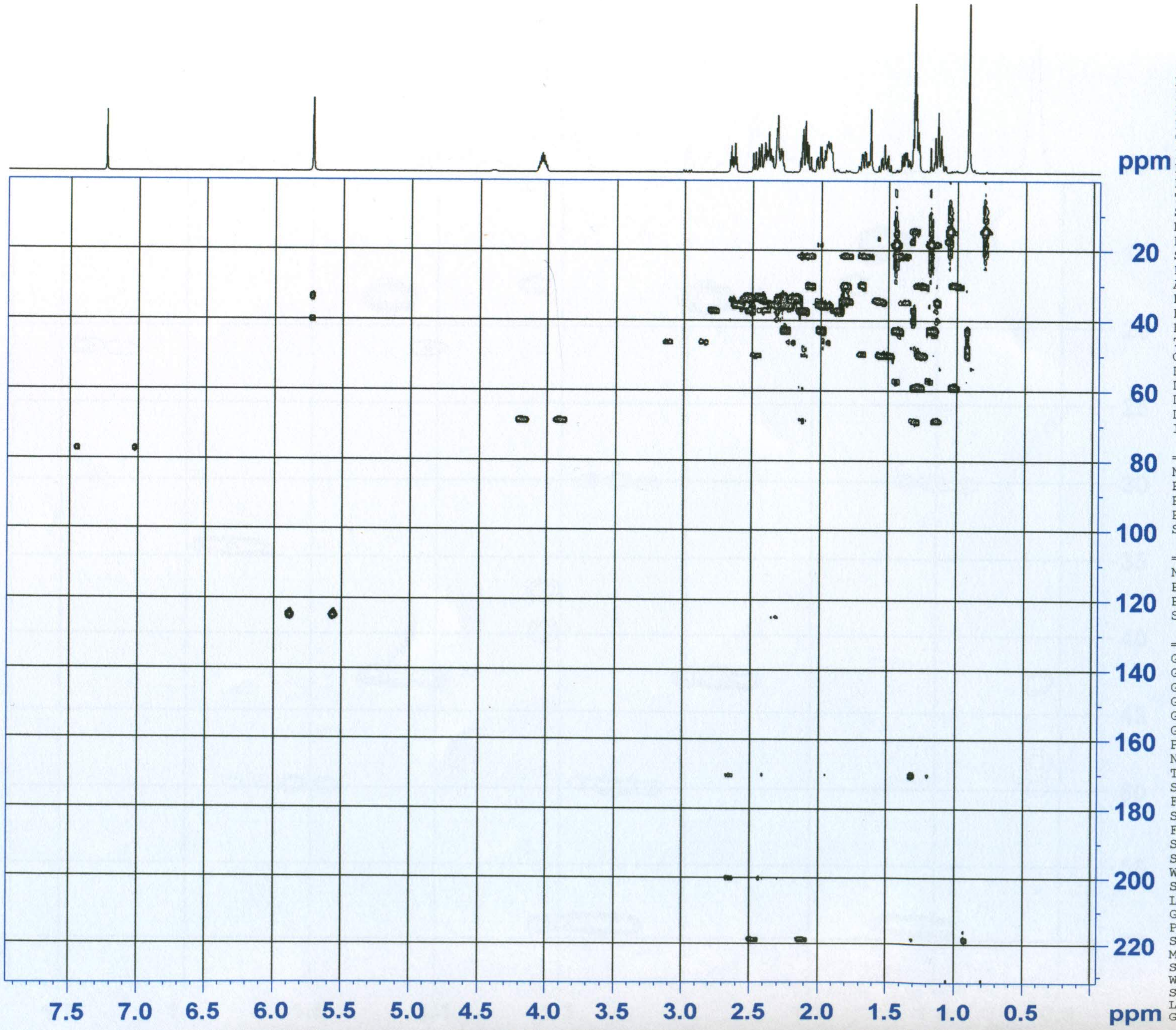

NAME	oct27
EXPNO	12
PROCNO	1
Date_	20091027
Time	19.45
INSTRUM	spect
PROBHD	5 mm BBI 1H-BB
PULPROG	hmbcgpndqf
TD	4096
SOLVENT	CDCl3
NS	64
DS	8
SWH	4006.410 Hz
FIDRES	0.978127 Hz
AQ	0.5113556 sec
RG	23170.5
DW	124.800 usec
DE	6.50 usec
TE	302.9 K
CNST13	145.0000000
D0	0.00000300 sec
D1	1.50000000 sec
D6	0.00344828 sec
D16	0.00020000 sec
IN0	0.00001715 sec

======== CHANNEL f1 ========

NUC1	1H
P1	6.70 usec
P2	13.40 usec
PL1	6.00 dB
SFO1	500.1320005 MHz

======== CHANNEL f2 ========

NUC2	13C
P3	11.10 usec
PL2	-3.00 dB
SFO2	125.7723769 MHz

====== GRADIENT CHANNEL =====

GPNAM1	SINE.100
GPNAM2	SINE.100
GPNAM3	SINE.100
GPZ1	50.00 %
GPZ2	30.00 %
GPZ3	40.10 %
P16	1000.00 usec
ND0	2
TD	256
SFO1	125.7724 MHz
FIDRES	113.981216 Hz
SW	232.000 ppm
FnMODE	QF
SI	1024
SF	500.1300232 MHz
WDW	QSINE
SSB	0
LB	0.00 Hz
GB	0
PC	4.00
SI	1024
MC2	QF
SF	125.7577908 MHz
WDW	QSINE
SSB	0
LB	0.00 Hz
GB	0

SALMAN ZAFAR/DR.IQBAL/TGF7-3-1/

AVANCE 500
LAB No. 109-B

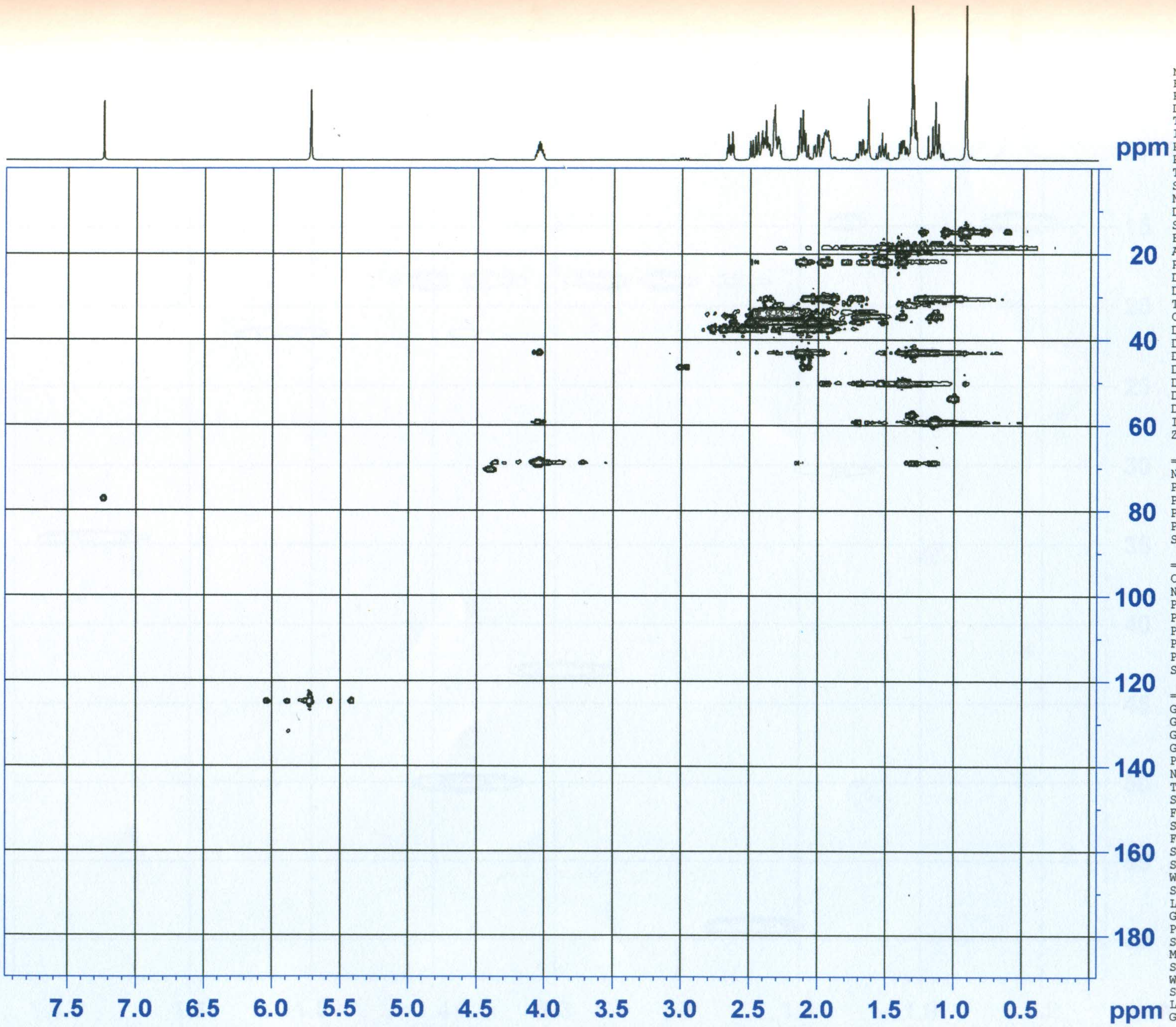

NAME	oct27
EXPNO	11
PROCNO	1
Date_	20091027
Time	15.58
INSTRUM	spect
PROBHD	5 mm BBI 1H-BB
PULPROG	hsqcetgpsi
TD	1024
SOLVENT	CDCl3
NS	32
DS	8
SWH	4006.410 Hz
FIDRES	3.912510 Hz
AQ	0.1279700 sec
RG	14596.5
DW	124.800 usec
DE	6.50 usec
TE	302.7 K
CNST2	145.0000000
D0	0.00000300 sec
D1	1.50000000 sec
D4	0.00172414 sec
D11	0.03000000 sec
D13	0.00000400 sec
D16	0.00015000 sec
D24	0.00110000 sec
IN0	0.00002090 sec
ZGOPTNS	
======== CHANNEL f1 ========	
NUC1	1H
P1	6.70 usec
P2	13.40 usec
P28	1000.00 usec
PL1	6.00 dB
SFO1	500.1320005 MHz
======== CHANNEL f2 ========	
CPDPRG2	garp
NUC2	13C
P3	11.10 usec
P4	22.20 usec
PCPD2	100.00 usec
PL2	-3.00 dB
PL12	18.00 dB
SFO2	125.7697360 MHz
====== GRADIENT CHANNEL =====	
GPNAM1	SINE.100
GPNAM2	SINE.100
GPZ1	80.00 %
GPZ2	20.10 %
P16	1500.00 usec
ND0	2
TD	256
SFO1	125.7697 MHz
FIDRES	93.344727 Hz
SW	190.000 ppm
FnMODE	Echo-Antiecho
SI	1024
SF	500.1300232 MHz
WDW	QSINE
SSB	2
LB	0.00 Hz
GB	0
PC	4.00
SI	1024
MC2	echo-antiecho
SF	125.7577908 MHz
WDW	QSINE
SSB	2
LB	0.00 Hz
GB	0

SALMAN ZAFAR/DR.IQBAL/TGF7-3-1/

LAB No. 109-B

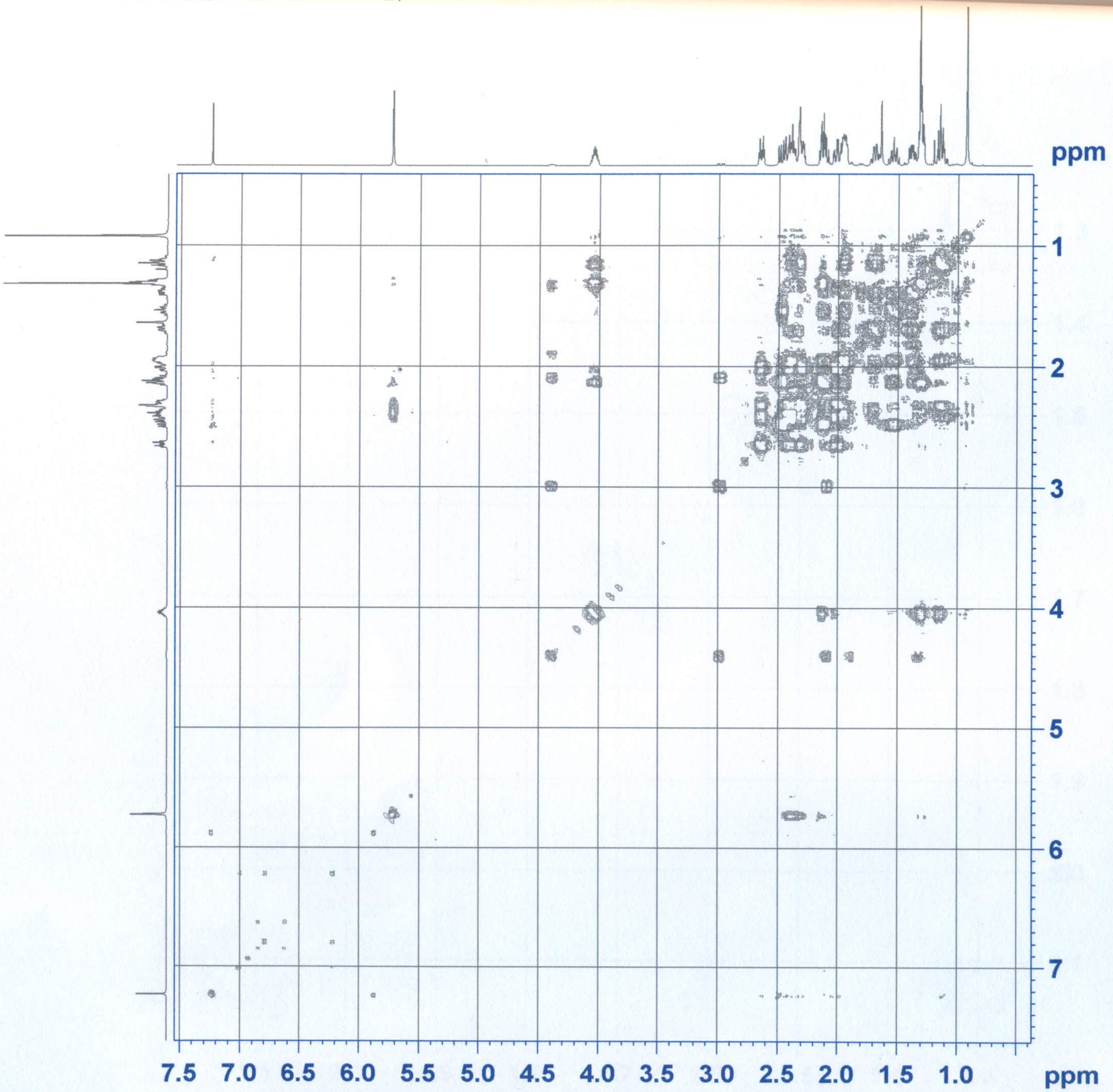

NAME	oct27
EXPNO	9
PROCNO	1
Date_	20091027
Time	12.00
INSTRUM	spect
PROBHD	5 mm BBI 1H-BB
PULPROG	cosydfqf
TD	2048
SOLVENT	CDCl3
NS	8
DS	8
SWH	4006.410 Hz
FIDRES	1.956255 Hz
AQ	0.2557652 sec
RG	71.8
DW	124.800 usec
DE	6.50 usec
TE	304.6 K
D0	0.00000300 sec
D1	1.50000000 sec
D13	0.00000400 sec
D20	0.00000200 sec
IN0	0.00024960 sec

======== CHANNEL f1 ========

NUC1	1H
P1	6.70 usec
PL1	6.00 dB
SFO1	500.1320005 MHz
ND0	1
TD	256
SFO1	500.132 MHz
FIDRES	15.650039 Hz
SW	8.011 ppm
FnMODE	QF
SI	1024
SF	500.1300232 MHz
WDW	SINE
SSB	0
LB	0.00 Hz
GB	0
PC	4.00
SI	1024
MC2	QF
SF	500.1300232 MHz
WDW	SINE
SSB	0
LB	0.00 Hz
GB	0

SALMAN ZAFAR/DR.IQBAL/TGF7-3-1/

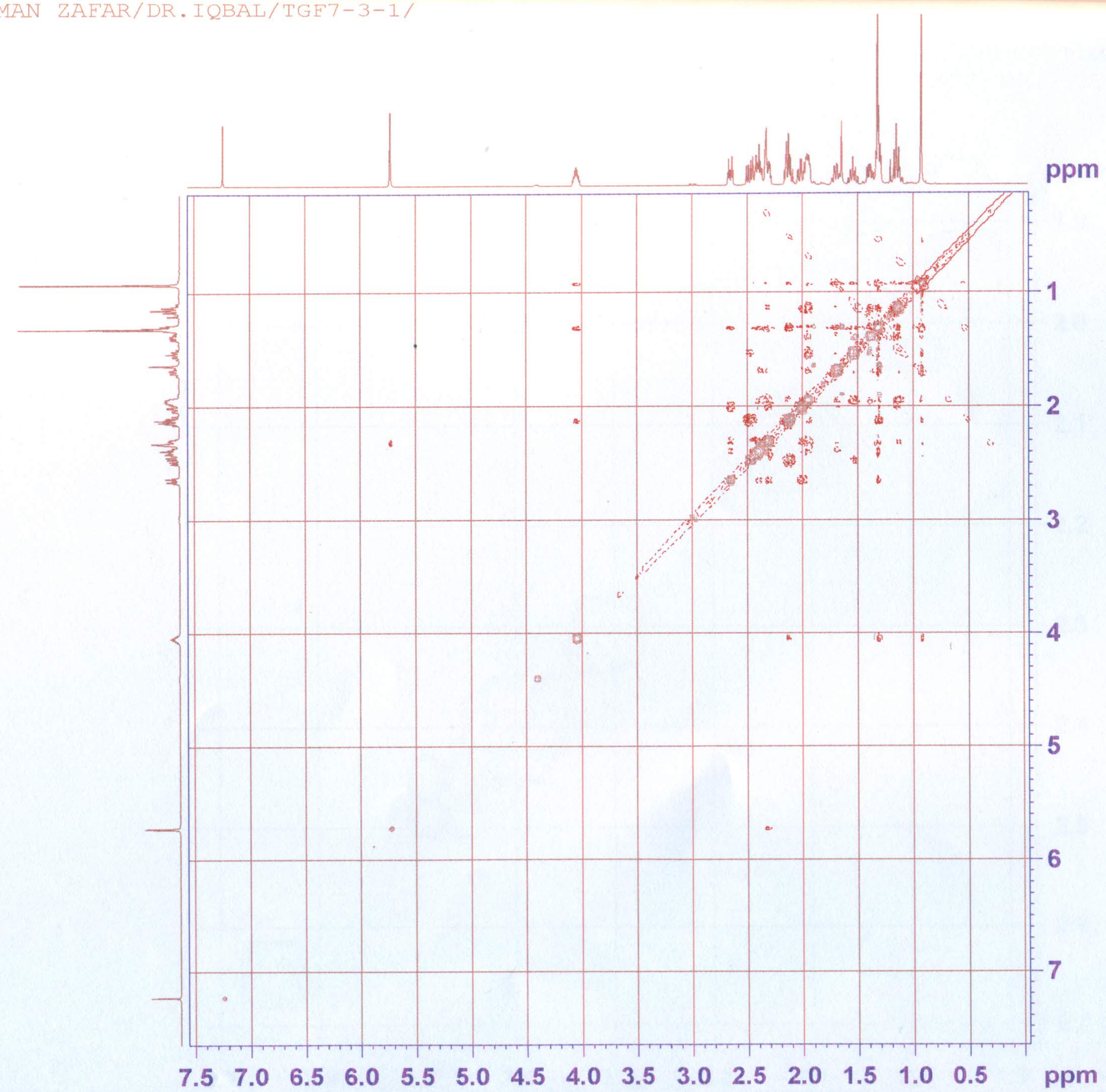

NAME	oct27	
EXPNO	10	
PROCNO	1	
Date_	20091027	
Time	13.02	
INSTRUM	spect	
PROBHD	5 mm BBI 1H-BB	
PULPROG	noesygpph	
TD	2048	
SOLVENT	CDCl3	
NS	16	
DS	2	
SWH	4006.410	Hz
FIDRES	1.956255	Hz
AQ	0.2557652	sec
RG	1625.5	
DW	124.800	usec
DE	6.50	usec
TE	304.1	K
D0	0.00011627	sec
D1	1.50000000	sec
D8	0.80000001	sec
D16	0.00020000	sec
IN0	0.00024960	sec

======== CHANNEL f1 ========

NUC1	1H	
P1	6.70	usec
P2	13.40	usec
PL1	6.00	dB
SFO1	500.1320005	MHz

====== GRADIENT CHANNEL =====

GPNAM1	SINE.100	
GPNAM2	SINE.100	
GPZ1	40.00	%
GPZ2	-40.00	%
P16	1000.00	usec
ND0	1	
TD	256	
SFO1	500.132	MHz
FIDRES	15.650039	Hz
SW	8.011	ppm
FnMODE	States-TPPI	
SI	1024	
SF	500.1300232	MHz
WDW	QSINE	
SSB	2	
LB	0.00	Hz
GB	0	
PC	4.00	
SI	1024	
MC2	States-TPPI	
SF	500.1300232	MHz
WDW	QSINE	
SSB	2	
LB	0.00	Hz
GB	0	
