# Supplementary material for: Molecular docking simulation studies on potent butyrylcholinesterase inhibitors obtained from microbial transformation of dihydrotestosterone
Source: Chem Cent J. 2013 Oct 8;7:164. doi: 10.1186/1752-153X-7-164 (PMC4126177; doi:10.1186/1752-153X-7-164)
Supplement: Additional file 6 — 1H-, 13C- and 2D-NMR spectra of compound 7. [file 1752-153X-7-164-S6.pdf]

Salman/Dr,Iqbal/Cdcl3/TGF-2/

AVANCE AV 300
LAB No. 11b

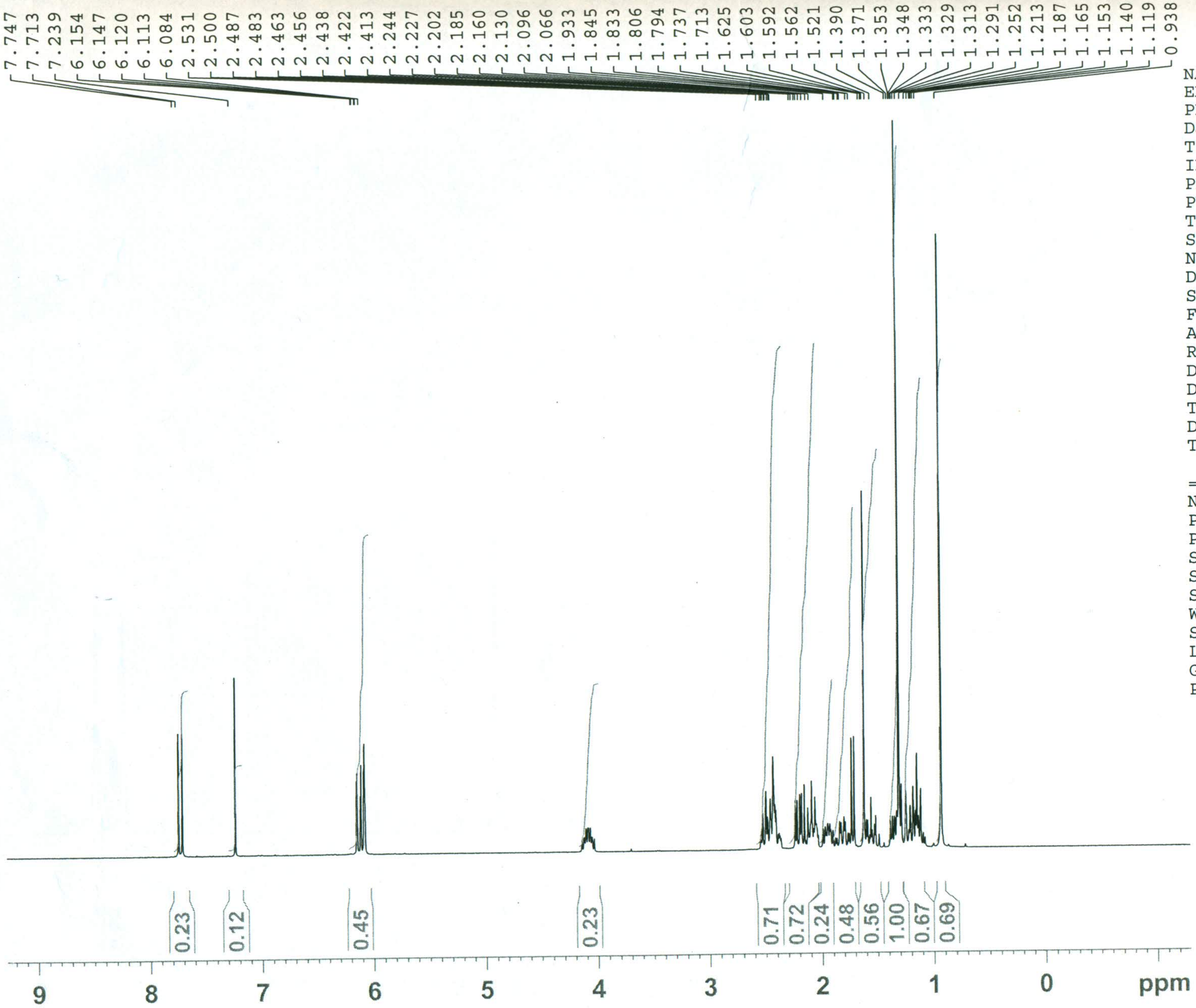

NAME	Aug18
EXPNO	2
PROCNO	1
Date_	20090818
Time	12.56
INSTRUM	spect
PROBHD	5 mm DUL 13C-1
PULPROG	zg30
TD	32768
SOLVENT	CDCl3
NS	128
DS	0
SWH	6172.839 Hz
FIDRES	0.188380 Hz
AQ	2.6542580 sec
RG	645.1
DW	81.000 usec
DE	6.50 usec
TE	300.0 K
D1	1.00000000 sec
TD0	1

======== CHANNEL f1 ========

NUC1	1H
P1	10.00 usec
PL1	-3.00 dB
SFO1	300.1321009 MHz
SI	16384
SF	300.1300122 MHz
WDW	EM
SSB	0
LB	0.30 Hz
GB	0
PC	1.00

Sample: IGF2

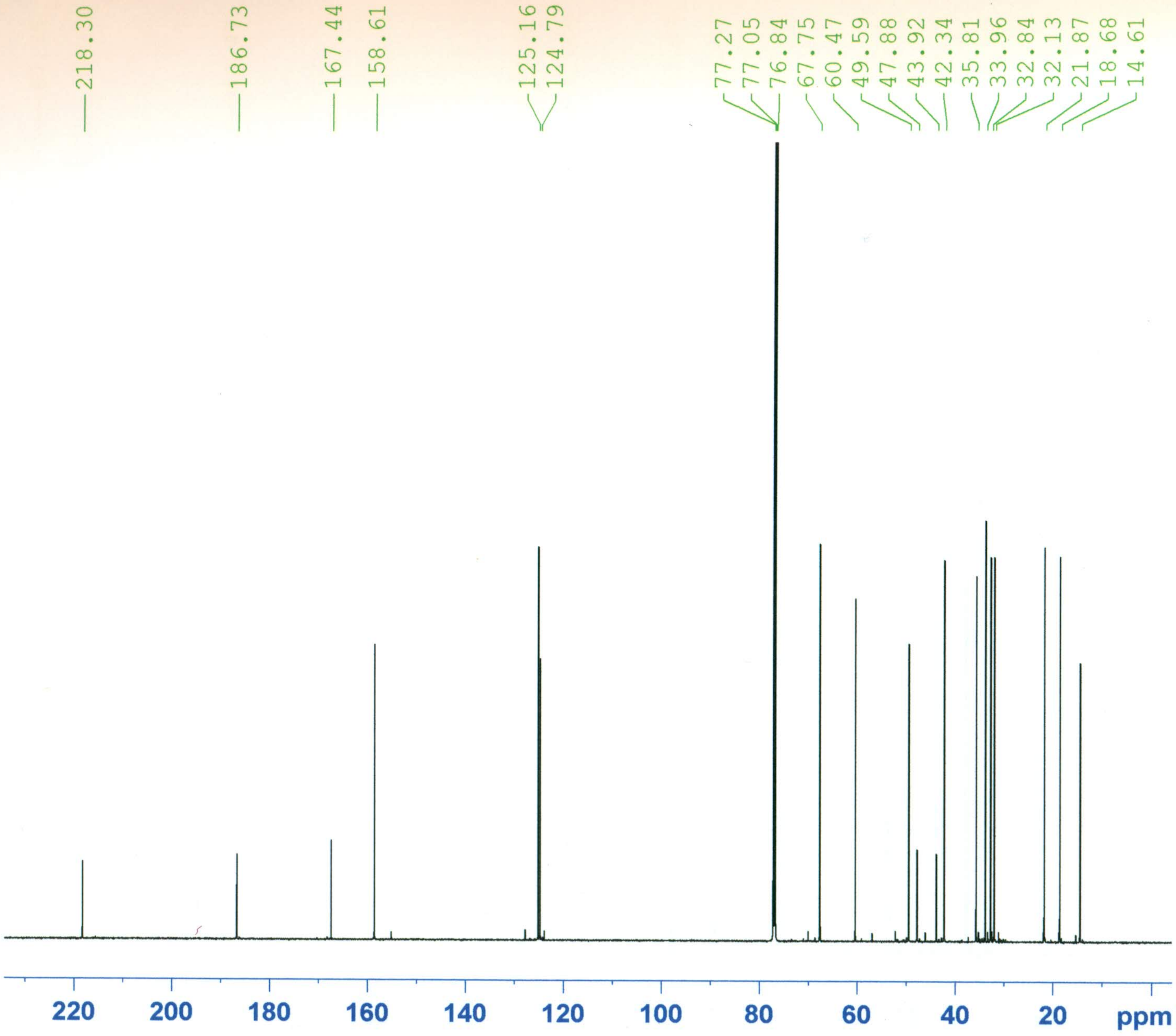

BRUKER

NAME	Oct 25	
EXPNO	6	
PROCNO	1	
Date_	20091026	
Time	4.55	
INSTRUM	spect	
PROBHD	5 mm CPTCI 1H-	
PULPROG	zgpg	
TD	65536	
SOLVENT	CDCl3	
NS	6168	
DS	2	
SWH	35971.223	Hz
FIDRES	0.548877	Hz
AQ	0.9110143	sec
RG	32768	
DW	13.900	usec
DE	6.50	usec
TE	293.7	K
D1	1.50000000	sec
D11	0.03000000	sec
TD0	12	

======== CHANNEL f1 ========

NUC1	13C	
P1	16.00	usec
PL1	2.00	dB
PL1W	66.40702820	W
SFO1	150.9453107	MHz

======== CHANNEL f2 ========

CPDPRG2	waltz16	
NUC2	1H	
PCPD2	65.00	usec
PL2	3.30	dB
PL12	22.06	dB
PL13	27.00	dB
PL2W	9.16420078	W
PL12W	0.12192553	W
PL13W	0.03909260	W
SFO2	600.2336014	MHz
SI	32768	
SF	150.9279540	MHz
WDW	EM	
SSB	0	
LB	1.00	Hz
GB	0	
PC	1.40	

VANCE AV 600
LAB. No. 108

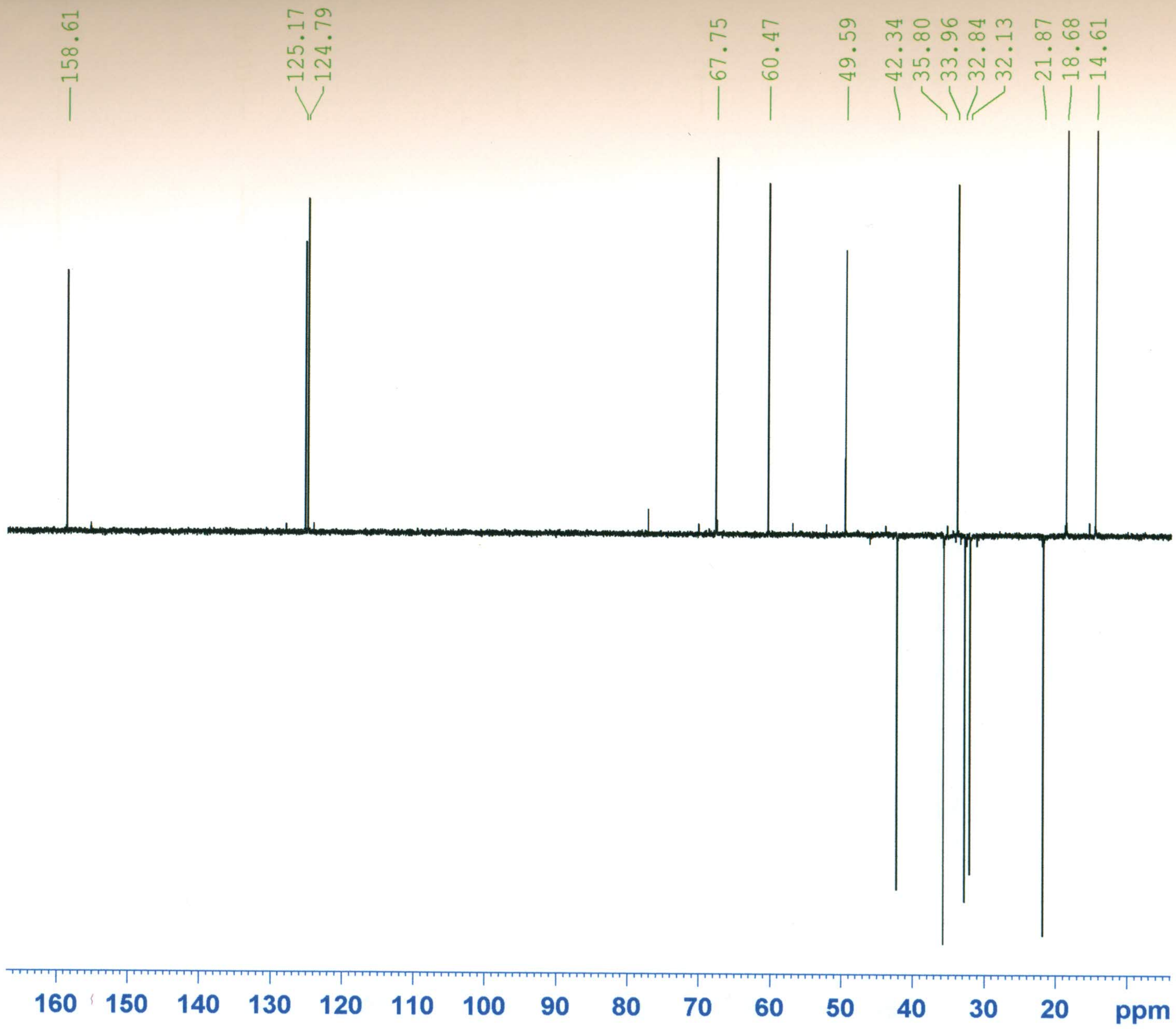

BRUKER

NAME	Oct 25	
EXPNO	7	
PROCNO	1	
Date_	20091026	
Time	8.47	
INSTRUM	spect	
PROBHD	5 mm CPTCI 1H-	
PULPROG	deptsp135	
TD	65536	
SOLVENT	CDCl3	
NS	444	
DS	2	
SWH	30303.031	Hz
FIDRES	0.462388	Hz
AQ	1.0814105	sec
RG	32768	
DW	16.500	usec
DE	6.50	usec
TE	294.1	K
CNST2	145.0000000	
D1	1.50000000	sec
D2	0.00344828	sec
D12	0.00002000	sec
TD0	6	
======== CHANNEL f1 ========		
NUC1	13C	
P1	16.00	usec
P12	2000.00	usec
PL0	120.00	dB
PL1	2.00	dB
PL0W	0.00000000	W
PL1W	66.40702820	W
SFO1	150.9430463	MHz
SP2	1.99	dB
SPNAM2	Crp60comp.4	
SPOAL2	0.500	
SPOFFS2	0.00	Hz
======== CHANNEL f2 ========		
CPDPRG2	waltz16	
NUC2	1H	
P3	7.50	usec
P4	15.00	usec
PCPD2	65.00	usec
PL2	3.30	dB
PL12	22.06	dB
PL2W	9.16420078	W
PL12W	0.12192553	W
SFO2	600.2324009	MHz
SI	32768	
SF	150.9279540	MHz
WDW	EM	
SSB	0	
LB	1.00	Hz
GB	0	
PC	1.40	

Sample: TGF2

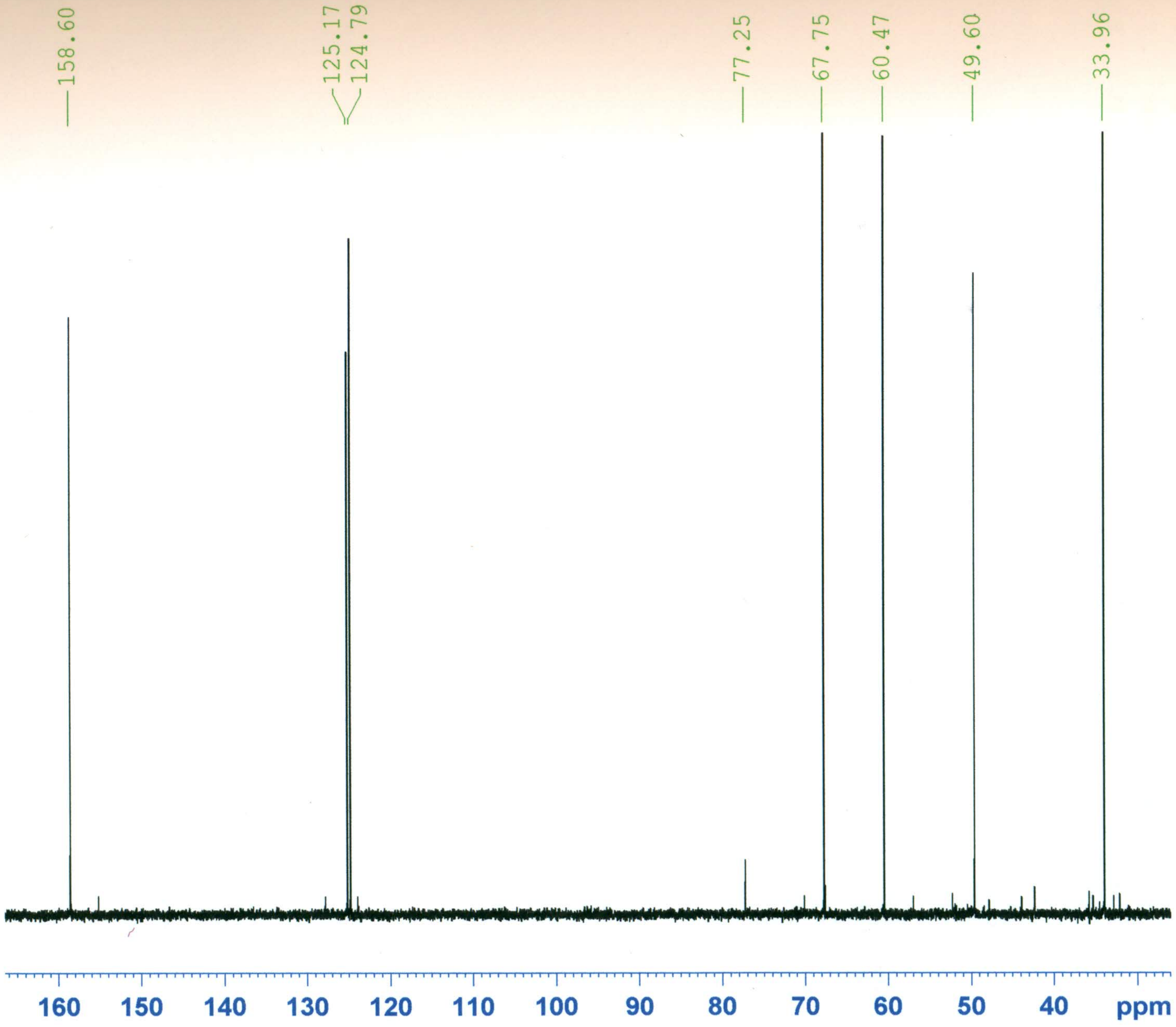

BRUKER

NAME Oct 25
EXPNO 8
PROCNO 1
Date_ 20091026
Time 8.58
INSTRUM spect
PROBHD 5 mm CPTCI 1H-
PULPROG deptsp90
TD 65536
SOLVENT CDCl3
NS 240
DS 2
SWH 30303.031 Hz
FIDRES 0.462388 Hz
AQ 1.0814105 sec
RG 32768
DW 16.500 usec
DE 6.50 usec
TE 294.2 K
CNST2 145.0000000
D1 1.50000000 sec
D2 0.00344828 sec
D12 0.00002000 sec
TD0 4

======== CHANNEL f1 ========
NUC1 13C
P1 16.00 usec
P12 2000.00 usec
PL0 120.00 dB
PL1 2.00 dB
PL0W 0.00000000 W
PL1W 66.40702820 W
SFO1 150.9430463 MHz
SP2 1.99 dB
SPNAM2 Crp60comp.4
SPOAL2 0.500
SPOFFS2 0.00 Hz

======== CHANNEL f2 ========
CPDPRG2 waltz16
NUC2 1H
P3 7.50 usec
P4 15.00 usec
PCPD2 65.00 usec
PL2 3.30 dB
PL12 22.06 dB
PL2W 9.16420078 W
PL12W 0.12192553 W
SFO2 600.2324009 MHz
SI 32768
SF 150.9279540 MHz
WDW EM
SSB 0
LB 1.00 Hz
GB 0
PC 1.40

AVANCE AV 600
LAB. No. 106

Sample; TGF2

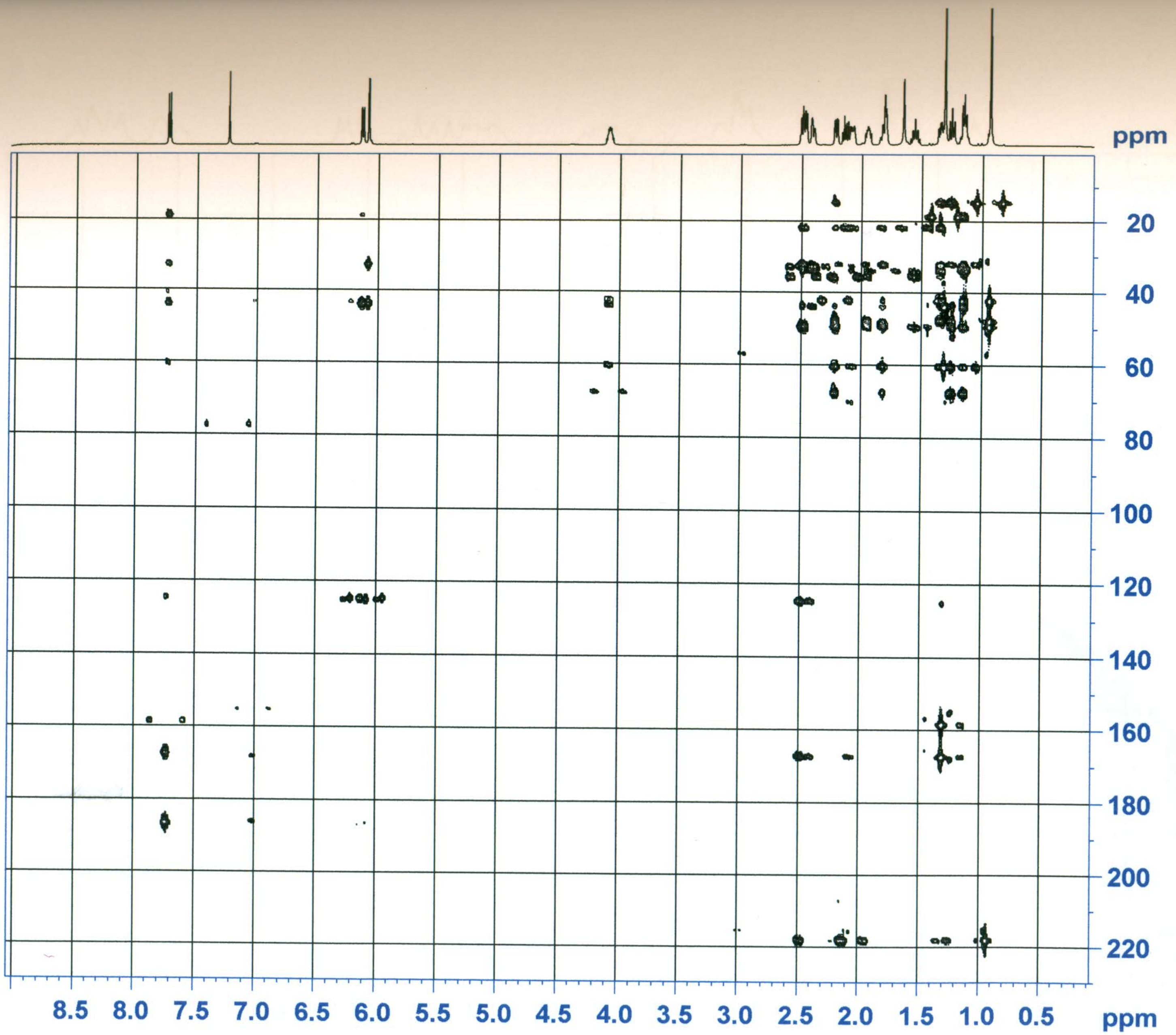

BRUKER

NAME	Oct 25	
EXPNO	5	
PROCNO	1	
Date_	20091025	
Time	23.50	
INSTRUM	spect	
PROBHD	5 mm CPTCI 1H-	
PULPROG	hmbcgplpndqf	
TD	4096	
SOLVENT	CDCl3	
NS	32	
DS	8	
SWH	5387.931	Hz
FIDRES	1.315413	Hz
AQ	0.3802516	sec
RG	32768	
DW	92.800	usec
DE	6.50	usec
TE	294.2	K
CNST2	145.0000000	
CNST13	13.0000000	
D0	0.00000300	sec
D1	1.50000000	sec
D2	0.00344828	sec
D6	0.03846154	sec
D16	0.00015000	sec
IN0	0.00001440	sec

======== CHANNEL f1 ========

NUC1	1H	
P1	7.40	usec
P2	14.80	usec
PL1	3.30	dB
PL1W	9.16420078	W
SFO1	600.2327611	MHz

======== CHANNEL f2 ========

NUC2	13C	
P3	10.00	usec
PL2	2.00	dB
PL2W	66.40702820	W
SFO2	150.9453107	MHz

====== GRADIENT CHANNEL =====

GPNAM1	SINE.100	
GPNAM2	SINE.100	
GPNAM3	SINE.100	
GPZ1	50.00	%
GPZ2	30.00	%
GPZ3	40.10	%
P16	2000.00	usec
ND0	2	
TD	256	
SFO1	150.9453	MHz
FIDRES	135.614929	Hz
SW	230.000	ppm
FnMODE	QF	
SI	1024	
SF	600.2300250	MHz
WDW	SINE	
SSB	0	
LB	0.00	Hz
GB	0	
PC	1.40	
SI	1024	
MC2	QF	
SF	150.9279540	MHz
WDW	SINE	
SSB	0	
LB	0.00	Hz
GB	0	

Sample: TGF2

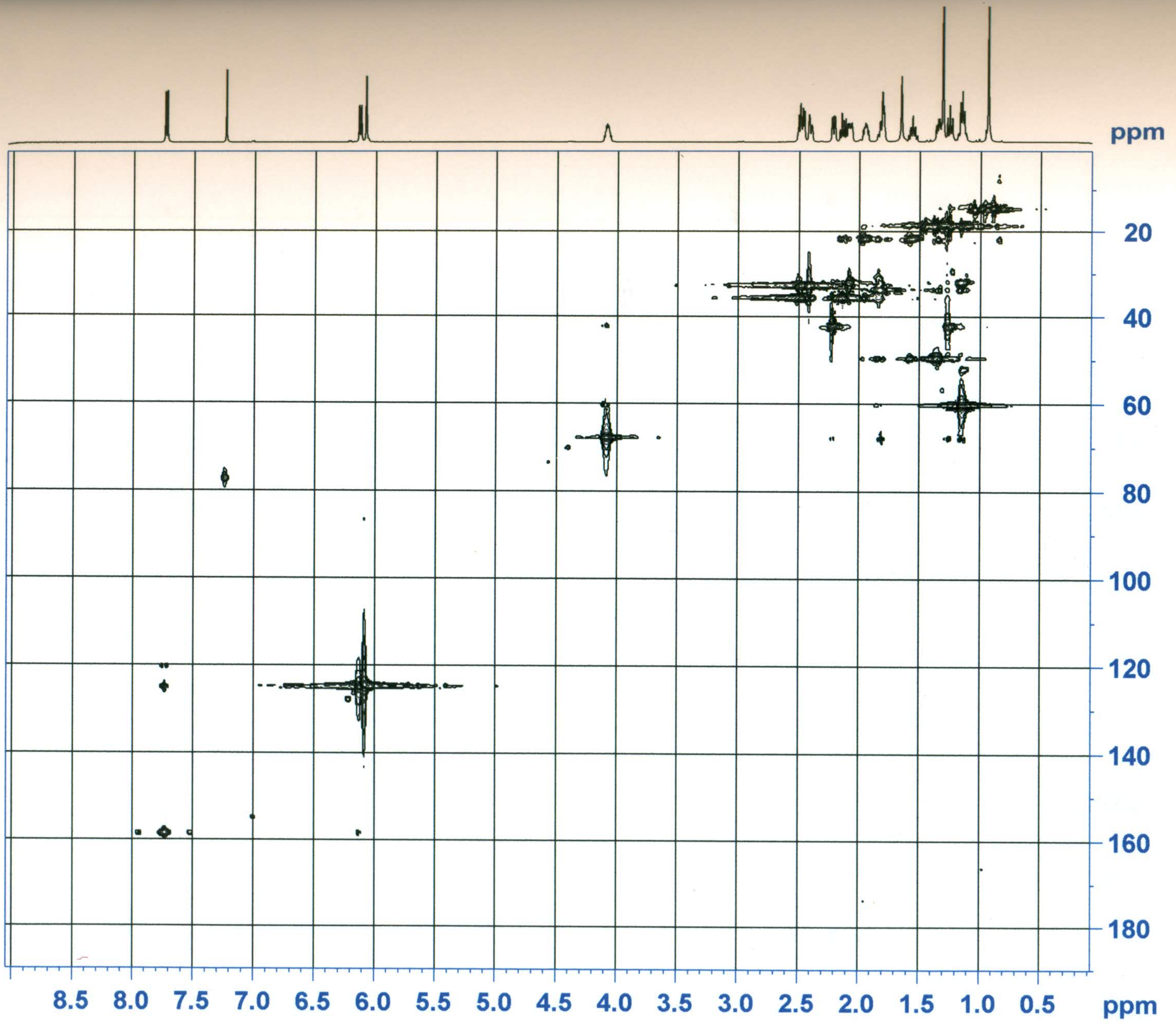

BRUKER

NAME	Oct 25	
EXPNO	4	
PROCNO	1	
Date_	20091025	
Time	20.08	
INSTRUM	spect	
PROBHD	5 mm CPTCI 1H-	
PULPROG	hsqcetgpsi	
TD	1024	
SOLVENT	CDCl3	
NS	32	
DS	8	
SWH	5387.931	Hz
FIDRES	5.261652	Hz
AQ	0.0951700	sec
RG	36780.8	
DW	92.800	usec
DE	6.50	usec
TE	294.5	K
CNST2	145.0000000	
D0	0.00000300	sec
D1	1.50000000	sec
D4	0.00172414	sec
D11	0.03000000	sec
D13	0.00000400	sec
D16	0.00015000	sec
D24	0.00110000	sec
IN0	0.00001745	sec
ZGOPTNS		
======== CHANNEL f1 ========		
NUC1	1H	
P1	7.40	usec
P2	14.80	usec
P28	0.50	usec
PL1	3.30	dB
PL1W	9.16420078	W
SFO1	600.2327611	MHz
======== CHANNEL f2 ========		
CPDPRG2	garp	
NUC2	13C	
P3	10.00	usec
P4	20.00	usec
PCPD2	60.00	usec
PL2	2.00	dB
PL12	17.56	dB
PL2W	66.40702820	W
PL12W	1.84592509	W
SFO2	150.9422922	MHz
====== GRADIENT CHANNEL =====		
GPNAM1	SINE.100	
GPNAM2	SINE.100	
GPZ1	80.00	%
GPZ2	20.10	%
P16	2000.00	usec
ND0	2	
TD	256	
SFO1	150.9423	MHz
FIDRES	112.027481	Hz
SW	190.000	ppm
FnMODE	Echo-Antiecho	
SI	1024	
SF	600.2300250	MHz
WDW	QSINE	
SSB	2	
LB	0.00	Hz
GB	0	
PC	4.00	
SI	1024	
MC2	echo-antiecho	
SF	150.9279540	MHz
WDW	QSINE	
SSB	2	
LB	0.00	Hz
GB	0	

AVANCE AV 600
LAB. NO. 108

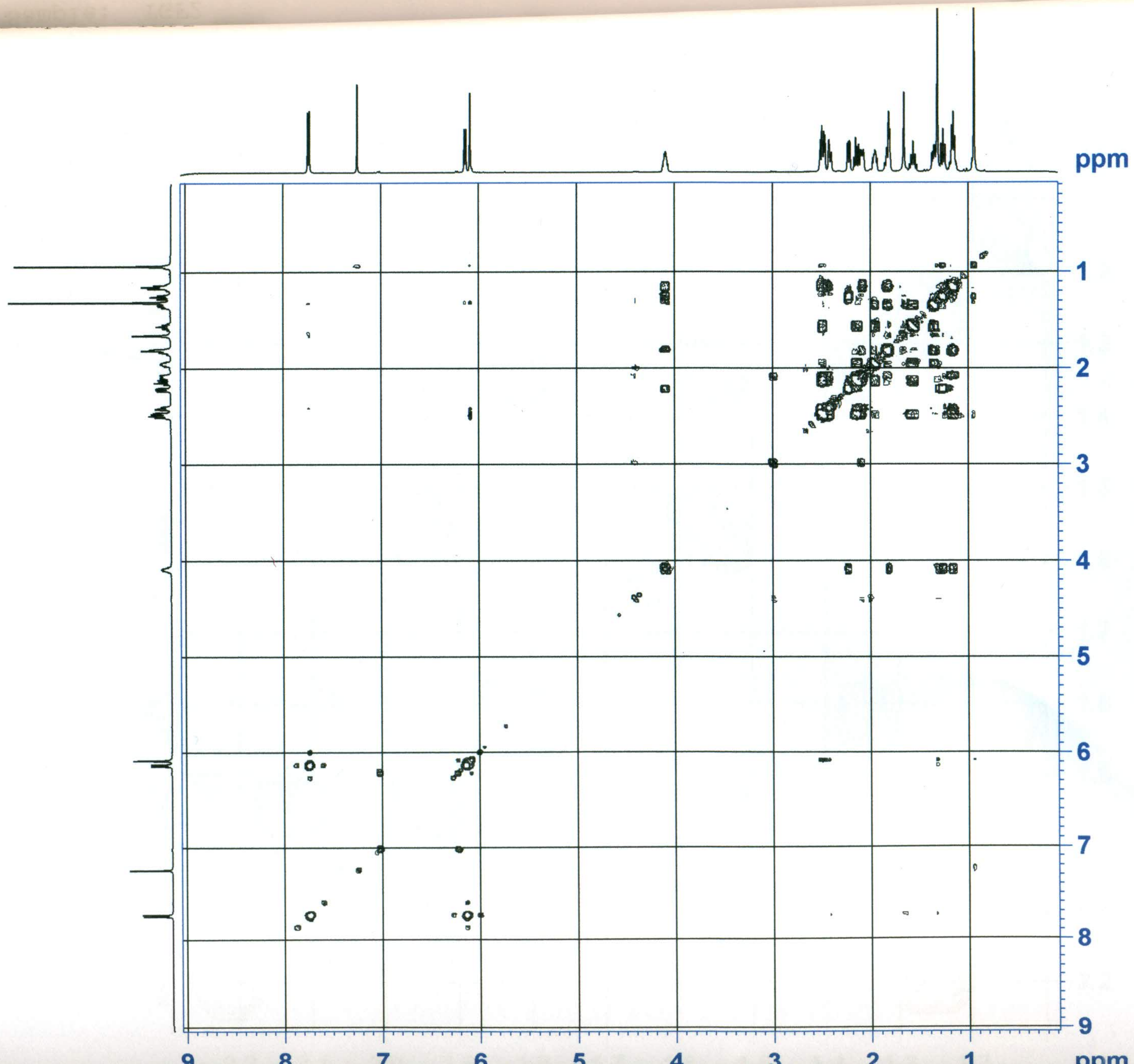

AVANCE AV 600
LAB. No. 106

NAME	Oct 25
EXPNO	2
PROCNO	1
Date_	20091025
Time	16.23
INSTRUM	spect
PROBHD	5 mm CPTCI 1H-
PULPROG	cosydfqf
TD	2048
SOLVENT	CDCl3
NS	8
DS	4
SWH	5387.931 Hz
FIDRES	2.630826 Hz
AQ	0.1901972 sec
RG	35.9
DW	92.800 usec
DE	6.50 usec
TE	294.8 K
D0	0.00000300 sec
D1	1.50000000 sec
D13	0.00000400 sec
D20	0.00000200 sec
IN0	0.00018560 sec

======== CHANNEL f1 ========

NUC1	1H
P1	7.40 usec
PL1	3.30 dB
PL1W	9.16420078 W
SFO1	600.2327611 MHz
ND0	1
TD	256
SFO1	600.2328 MHz
FIDRES	21.046606 Hz
SW	8.976 ppm
FnMODE	QF
SI	1024
SF	600.2300250 MHz
WDW	QSINE
SSB	0
LB	0.00 Hz
GB	0
PC	1.40
SI	1024
MC2	QF
SF	600.2300250 MHz
WDW	QSINE
SSB	0
LB	0.00 Hz
GB	0

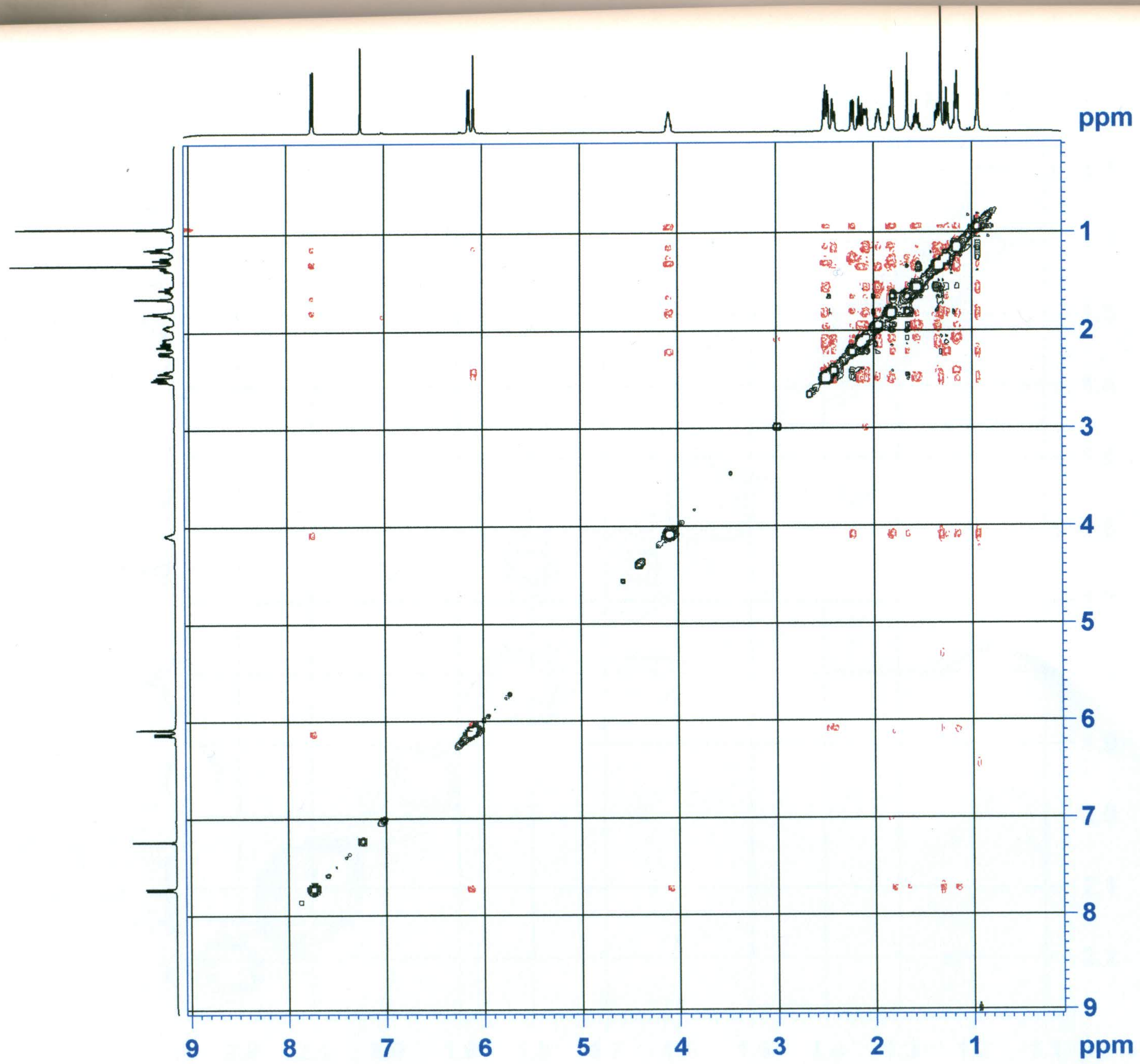

BRUKER

[illegible]VANCE AV 600
[illegible] No. 108

NAME	Oct 25
EXPNO	3
PROCNO	1
Date_	20091025
Time_	17.23
INSTRUM	spect
PROBHD	5 mm CPTCI 1H-
PULPROG	noesygpph
TD	1024
SOLVENT	CDCl3
NS	16
DS	4
SWH	5387.931 Hz
FIDRES	5.261652 Hz
AQ	0.0951700 sec
RG	57
DW	92.800 usec
DE	6.50 usec
TE	294.7 K
D0	0.00008338 sec
D1	1.50000000 sec
D8	0.80000001 sec
D16	0.00015000 sec
IN0	0.00018560 sec

======== CHANNEL f1 ========

NUC1	1H
P1	7.40 usec
P2	14.80 usec
PL1	3.30 dB
PL1W	9.16420078 W
SFO1	600.2327611 MHz

====== GRADIENT CHANNEL =====

GPNAM1	SINE.100
GPNAM2	SINE.100
GPZ1	40.00 %
GPZ2	-40.00 %
P16	2000.00 usec
ND0	1
TD	256
SFO1	600.2328 MHz
FIDRES	21.046606 Hz
SW	8.976 ppm
FnMODE	States-TPPI
SI	1024
SF	600.2300250 MHz
WDW	QSINE
SSB	2
LB	0.00 Hz
GB	0
PC	1.40
SI	1024
MC2	States-TPPI
SF	600.2300250 MHz
WDW	QSINE
SSB	2
LB	0.00 Hz
GB	0
